# Synthesis, Characterization, and Structural Analysis of **AM**[Al(NON^Dipp^)(H)(SiH_2_Ph)] (**AM** = Li, Na, K, Rb, Cs) Compounds, Made Via Oxidative Addition of Phenylsilane to Alkali Metal Aluminyls

**DOI:** 10.1021/acs.inorgchem.2c03010

**Published:** 2022-11-23

**Authors:** Gerd M. Ballmann, Matthew J. Evans, Thomas X. Gentner, Alan R. Kennedy, J. Robin Fulton, Martyn P. Coles, Robert E. Mulvey

**Affiliations:** † WestCHEM, Department of Pure and Applied Chemistry, University of Strathclyde, Glasgow G1 1XL, U.K.; ‡ School of Chemical and Physical Sciences, Victoria University of Wellington, P.O. Box 600, Wellington 6140, New Zealand

## Abstract

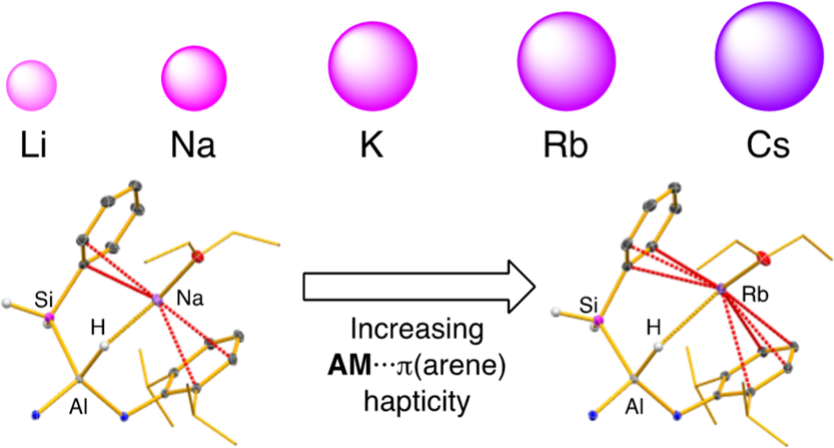

We report the oxidative addition of phenylsilane to the complete series of alkali metal (**AM**) aluminyls [**AM**{Al(NON^Dipp^)}]_2_ (**AM** = Li, Na, K, Rb, and Cs). Crystalline products (**1-AM**) have been isolated as ether or THF adducts, [**AM**(L)_*n*_][Al(NON^Dipp^)(H)(SiH_2_Ph)] (**AM** = Li, Na, K, Rb, L = Et_2_O, *n* = 1; **AM** = Cs, L = THF, *n* = 2). Further to this series, the novel rubidium rubidiate, [{Rb(THF)_4_}_2_(Rb{Al(NON^Dipp^)(H)(SiH_2_Ph)}_2_)]^+^ [Rb{Al(NON^Dipp^)(H)(SiH_2_Ph)}_2_]^−^, was isolated during an attempted recrystallization of Rb[Al(NON^Dipp^)(H)(SiH_2_Ph)] from a hexane/THF mixture. Structural and spectroscopic characterizations of the series **1-AM** confirm the presence of μ-hydrides that bridge the aluminum and alkali metals (**AM**), with multiple stabilizing **AM**···π(arene) interactions to either the Dipp- or Ph-substituents. These products form a complete series of soluble, alkali metal (hydrido) aluminates that present a platform for further reactivity studies.

## Abstract 2

Oxidative addition of phenylsilane to alkali metal (**AM**) aluminyls affords a full series of (silyl)(hydrido) aluminates for **AM** = Li, Na, K, Rb, and Cs, with an increased tendency for **AM**···π(arene) for the larger **AM** cations.

## Introduction

Since the isolation of the first stable molecular Al(I) complex in 1981,^1^ low-valent aluminum chemistry has been established as a fertile area of main group chemistry research.^2^ Early work largely focused on neutral species, with the chemistry of Al(BDI^Dipp^) (BDI^Dipp^ = [HC{C(Me)NDipp}_2_]^−^, Dipp = 2,6-*i*Pr_2_C_6_H_3_)^3^ and [Cp*Al]_4_^1^ dominating the area. Among the myriad of reactions that have been established with these species, the activation of chemical bonds via an oxidative addition to afford Al(III) derivatives has featured prominently.^4^

**Figure 1:**
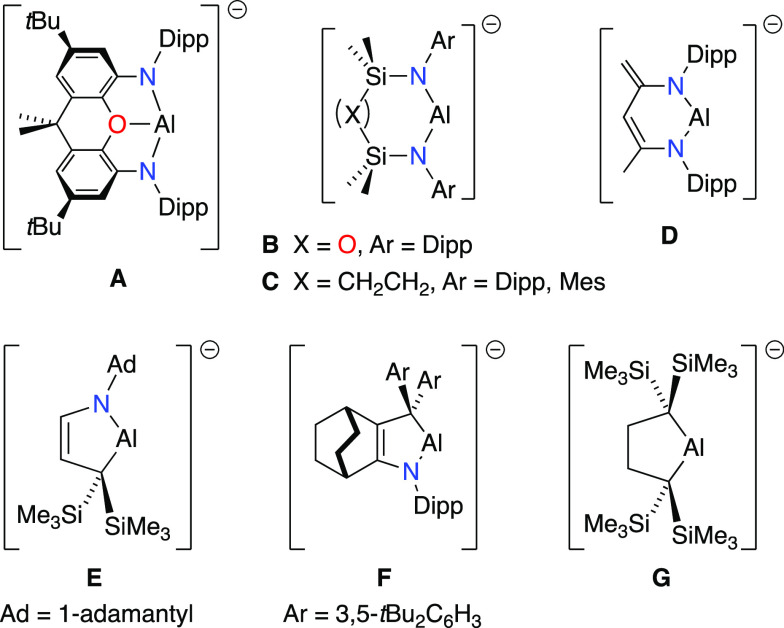
Current family of alkali metal aluminyl anions. **A** = [Al(_xanth_NON^Dipp^)]^−^, where _xanth_NON^Dipp^ = [4,5-(DippN)_2_-2,7-*t*Bu_2_-9,9-Me_2_-xanthene]^2–^ and Dipp = 2,6-*i*Pr_2_C_6_H_3_; **B** = [Al(NON^Dipp^)], where NON^Dipp^ = [O(SiMe_2_NDipp)_2_]^2–^; **C** = [Al(CH_2_SiMe_2_NAr)_2_]^−^, where Ar = Dipp, Mes; **D** = [Al(BDI-H)]^−^, where BDI-H = [DippNC(Me)=C(H)C(=CH_2_)NDipp]^2–^; **E** = [Al(AdN(CH)_2_C{SiMe_3_}_2_)]^−^, where Ad = 1-adamantyl; **F** = [Al(DippN{CCH(CH_2_)CH_2_}_2_CAr_2_)]^−^, where {CCH(CH_2_)CH_2_}_2_ = a substituted bicyclo[2.2.2]oct-2-ene group and Ar = 3,5-*t*Bu_2_C_6_H_3_; **G** = [Al(CH_2_C{SiMe_3_}_2_)_2_]^−^.

In 2018, a new class of anionic Al(I) complex entered the scene,^5^ prompting a surge in interest in this field.^6^ The initially reported compound consisted of a three-coordinate aluminum center supported by a diamido ligand set built around the xanthene scaffold [_xanth_NON^Dipp^]^2–^ (**A**), with a potassium cation to balance the charge.^5^ This report was rapidly followed by examples of two-coordinate diamido-(**B**-**D**),^7^ alkyl-amido-(**E**,**F**),^8^ and dialkyl-(**G**)^9^ aluminyl anions, all of which were isolated as their potassium salts (Figure 1). These compounds are able to activate strong and otherwise inert bonds in small molecule substrates, including H_2_,^5,10^ CO_2_,^11^ and CO.^12^

The concept of alkali metal mediation (AMM) has been presented to highlight the often underappreciated role of group 1 metals in enabling reactions often involving bimetallic systems, in which an alkali metal (**AM**) is partnered with a metal from the p- or d-block of the periodic table.^13^ It recognizes cooperative/synergistic interactions between the **AM** and other metallic elements, without which a specific chemical transformation may not occur (or may not proceed efficiently).^14^ The partnership between the alkali metal cation and the aluminum center of the aluminyl anion lends itself to the possibility of AMM during reactivity and offers an opportunity to examine the role of **AM** in this emerging area of chemistry. Despite this potential, aluminyl chemistry research has to date focused almost exclusively on potassium complexes, precluding the chance to study AMM in these systems. The synthesis of aluminyl compounds with a series of **AM** cations and the ability to control the nature and extent of the **AM**···Al interactions will be key to understanding the aluminyl chemistry and unlocking the potential of these systems.

It has recently been noted that it is not possible to access the [Al(_xanth_NON^Dipp^)]^−^ anion via reduction of the iodide precursor using Li or Na metal, leading instead to either decomposition (**AM** = Li) or formation of the Al(II) dimer (**AM** = Na).^15^ An alternative route to the lithium–aluminyl complex (_xanth_NON^Dipp^)Al–Li(Et_2_O)_2_ has been presented via metathesis of potassium aluminyl with lithium iodide, although this highly reactive product could only be isolated in low yield. These factors have thus far prevented systematic studies concerning the influence that the **AM** has on the chemical reactivity of [Al(_xanth_NON^Dipp^)]^−^ aluminyl. In contrast, we have reported the reduction of (NON^Dipp^)Al–I (**H**), with both lithium and sodium metal proceeding smoothly to afford the corresponding lithium and sodium aluminyls, [**AM**{Al(NON^Dipp^)}]_2_ (**AM** = Li, Na), that crystallize as slipped contacted dimeric pairs (CDPs) in which the aluminum engages in a single Al···**AM** interaction.^10^ Furthermore, the reaction of **H** with graphitic rubidium and cesium (RbC_8_ or CsC_8_)^16^ affords the corresponding heavier alkali metal aluminyls, [**AM**{Al(NON^Dipp^)}]_2_ (**AM** = Rb, Cs).^17^ These compounds, together with the initially prepared potassium aluminyl,^7a^ provide an opportunity to probe any AMM effects for the series of aluminyl anions incorporating the full complement of stable alkali metals, Li–Cs.

Harder and co-workers have recently expanded the series of aluminyls containing the [Al(BDI-H)]^−^ ([BDI-H]^2–^ = [DippNC(Me)=C(H)C(=CH_2_)NDipp]^2–^; **D**Figure 1) anion to encompass the full series [**AM**{Al(BDI-H)}]_2_ (**AM** = Li–Cs).^18^ Analogous to our results, the lithium and sodium derivatives have been isolated as the slipped CDPs and can be easily converted into the corresponding monomeric ion pairs (MIPs), (BDI-H)Al–Li(Et_2_O)_2_, and (BDI-H)Al–Na(Et_2_O) (TMEDA), when exposed to coordinating solvents. However, a difference is observed between the crystal structures of Rb and Cs within each series. We had noted previously that the monomeric units in [**AM**{Al(NON^Dipp^)}]_2_ (**AM** = Rb, Cs) CDPs were twisted relative to one another, rationalized as a requirement for the accommodation of the larger metal cations.^17^ However, the monomeric units in [**AM**{Al(BDI-H)}]_2_ (**AM** = Rb, Cs) CDPs are strictly coplanar due to a crystallographic center of inversion, indicating that a twist is not required in these structures. Despite these differences, the Al···Al distance is largely consistent (e.g., [Rb{Al(NON^Dipp^)}]_2_ = 5.548(1) Å, [Rb{Al(BDI-H)}]_2_ = 5.489(6) Å; [Cs{Al(NON^Dipp^)}]_2_ = 5.752(1) Å, [Cs{Al(BDI-H)}]_2_ = 5.7334(17) Å/6.1086(19) Å).

The relative dimerization energies calculated for 2 **AM**[Al(BDI-H)] → [**AM**{Al(BDI-H)}]_2_ show a regular decrease as the size of the alkali metal increases.^18^ However, when the opposing solvation effects [polarizable continuum model (PCM) = benzene, which favor the monomer due to solvent–metal interactions] and dispersion effects (which favor the dimer through attractive interactions between ligands) are incorporated in the calculations, the trend is less well defined with, for example, the Δ*G* value for **AM** = Na (−21.1 kcal mol^–1^) less than that for **AM** = K (−25.4 kcal mol^–1^). We have noted a similar trend with the 2 **AM**[Al(NON^Dipp^)] → [**AM**{Al(NON^Dipp^)}]_2_ system,^17^ wherein PCM = toluene with a Δ*G* value for **AM** = Na (−26.7 kcal mol^–1^) less than that for **AM** = K (−33.6 kcal mol^–1^). In both cases, the calculation indicates the dimerization enthalpies for heavier alkali metal derivatives Rb and Cs trend toward being less exothermic (−19.6 kcal mol^–1^ {Rb}, −14.3 kcal mol^–1^ {Cs} for [(BDI-H)]^2–^ system; −28.2 kcal mol^–1^ {Rb}, −24.9 kcal mol^–1^ {Cs} for [NON^Dipp^]^2–^ system). We consider these factors as important when examining the reactivity of these species, as we anticipate the monomeric alkali metal aluminyls to be considerably more reactive.

**Scheme 1:**
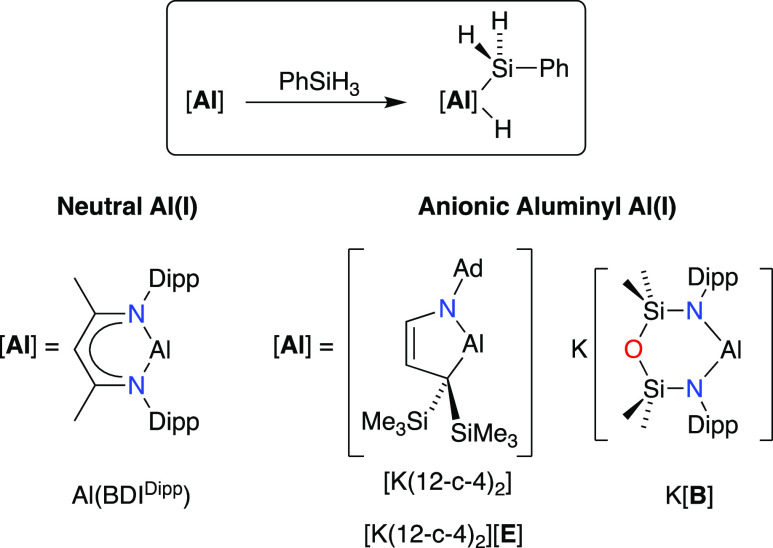
Oxidative Addition of Phenylsilane to Al(I) Complexes to Afford (silyl)(hydrido) Aluminum Products Al(BDI^Dipp^)(H)(SiH_2_Ph), [K(12-c-4)_2_][(AdN-CH=CHC{SiMe_3_}_2_)Al(H)(SiH_2_Ph)] ([K(12-c-4)_2_][**E**(H)(SiH_2_Ph)]), and K[Al(NON^Dipp^)(H)(SiH_2_Ph)] (K[**B**(H)(SiH_2_Ph)])

We have reported that the dimeric potassium aluminyl [K{Al(NON^Dipp^)}]_2_ is active toward the oxidative addition of polar and non-polar E–H bonds, including Si–H, P–H, O–H, N–H, and H–H bonds.^10,19^ The synthesis is a convenient route to soluble alkali metal (hydrido) aluminates, K[Al(NON^Dipp^)(E)(H)]^−^, which are in themselves an interesting class of molecular main group metal hydrides that offer the potential for further useful reactivity.^20^ To examine any AMM effects, we have selected the reaction of our aluminyl systems with phenylsilane (PhSiH_3_) as this can be benchmarked against other neutral and anionic Al(I) systems (Scheme 1).^8a,21^ We report herein the first examples of an oxidative addition reaction to a complete series of alkali metal aluminyls [**AM**{Al(NON^Dipp^)}]_2_ (**AM** = Li, Na, K, Rb, Cs), thereby exploiting a rare opportunity to systematically examine the influence of the alkali metal counter ion on this fundamental reaction type as well as on the structure of the products formed.

## Results and Discussion

**Scheme 2:**
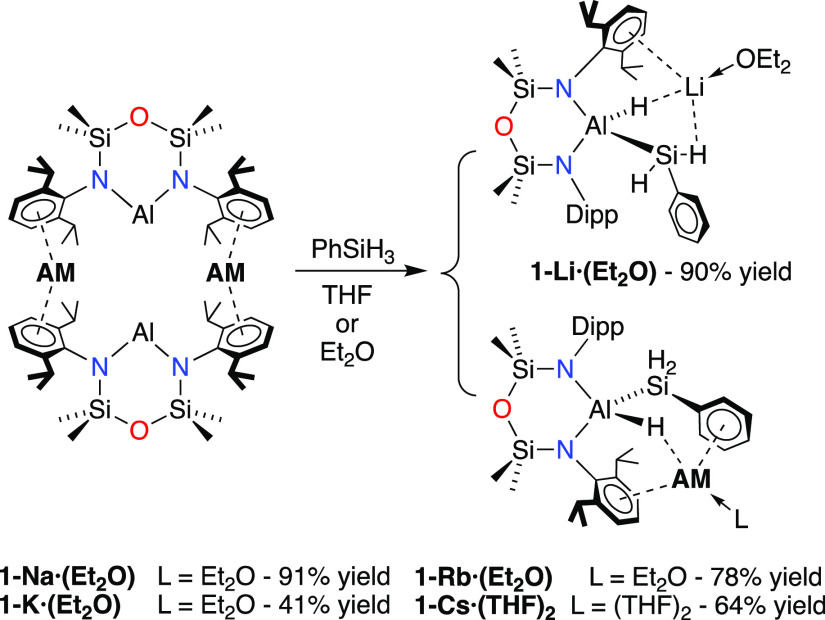
Synthesis of **1-AM**, Isolated as **1-AM·(Et**_**2**_**O)** (**AM** = Li, Na, K Rb) and **1-AM·(THF)**_**2**_ for **AM** = Cs

The addition of phenylsilane to a diethyl ether (**AM** = Li) or toluene (**AM** = Na, K^a^) solution of [**AM**{Al(NON^Dipp^)}]_2_ yielded the corresponding aluminum(III) (silyl)(hydrido) complexes **AM**[Al(NON^Dipp^)(H)(SiH_2_Ph)] (**1-AM**, Scheme 2). For the heavier congeners with **AM** = Rb and Cs, the reaction was carried out in hexane, resulting in precipitation of the crude product as a colorless solid. Purification was achieved by crystallization from Et_2_O (**AM** = Li, Na, K, Rb) or THF (**AM** = Cs), with the product isolated as the solvated species **1-AM·(solvent)**_***n***_, (**AM** = Li, Na, K and Rb: **solvent** = Et_2_O, *n* = 1; **AM** = Cs: **solvent** = THF, *n* = 2).

The ^1^H NMR spectra of the isolated products show two sharp singlets that each integrate as 6H for the Si*Me*_*2*_ groups of the NON^Dipp^-ligand backbone, indicating a reduction in the molecular symmetry and consistent with a [Al(NON^Dipp^)(X)(X′)]^−^ (where X ≠ X′) substituted center. Distinct resonances for the Si*H*_*2*_ protons were observed as either a resolved doublet (**AM** = Li, Rb, Cs) or a broad singlet (**AM** = Na, K). We note a solvent-dependent chemical shift for this resonance, with a higher field signal observed when the ^1^H NMR spectra are acquired in THF-*d*_8_ compared with those obtained in C_6_D_6_ (or a C_6_D_6_ dominant solvent mixture). This is best illustrated for **1-Li·(Et**_**2**_**O)** (δ_H_ 3.67 in C_6_D_6_, 3.18 in THF-*d*_8_) and **1-Cs·(THF)**_**2**_ (δ_H_ 3.62 in C_6_D_6_/THF-*d*_8_ (4:1), 3.37 in THF-*d*_8_). Although it is not possible to unequivocally ascribe this shift to the presence of Si*H*_*2*_···**AM** interactions in the solution, it does suggest that changes in the solvation of the **AM** affect the environment of the [Al(NON^Dipp^)(H)(SiH_2_Ph)]^−^ component of these complexes.

In contrast to the previously reported products Al(BDI^Dipp^)(H)(SiH_2_Ph) and [K(12-c-4)_2_][(AdN-CH=CH–C{SiMe_3_}_2_)Al(H)(SiH_2_Ph)] ([K(12-c-4)_2_][**E**(H)(SiH_2_Ph)]) (Scheme 1), for which a ^29^Si resonance was observed for the silyl ligand at δ_Si_ = −74.0,^21^ and δ_Si_ = −72.0,^8a^ respectively, the corresponding ^29^Si NMR signals for **1-AM** were not observed. Furthermore, the ^1^H NMR resonances for the Al*H* ligands could not be detected for any of the products, consistent with observations for related systems^19,21^ and attributed to the proximity of the quadrupolar ^27^Al nucleus (*I* = 5/2). The existence of both Al–H- and Si–H-containing groups was, however, verified from characteristic stretches in the IR spectra, where distinct absorptions were observed in the regions 1648–1686 and 2052–2084 cm^–1^, respectively.^b^

Despite the use of several types and combinations of donor groups in the supporting ligands at the aluminum center (vide supra), the ^27^Al NMR signal has not been reported for any of the parent aluminyl complexes, suggesting an inherent difficulty in observing a signal for these low-coordinate anionic species.^5,7−9^ That said, we have previously noted that the products of oxidative addition that contain aluminum in the +3 oxidation state are no longer silent in ^27^Al NMR experiments,^10,19^ and therefore, the appearance of a signal in the ^27^Al NMR spectra offers further supporting evidence for the formation of the desired products of oxidative addition. Each of the compounds **1-AM** shows broad resonances in the ^27^Al NMR spectrum for the [Al(NON^Dipp^)(H)(SiH_2_Ph)]^−^ anion in the range δ_Al_ 121–127. In addition, a signal in the ^7^Li NMR spectrum at δ_Li_ 2.72 confirmed the presence of lithium in **1-Li·(Et**_**2**_**O)**.

**Figure 2:**
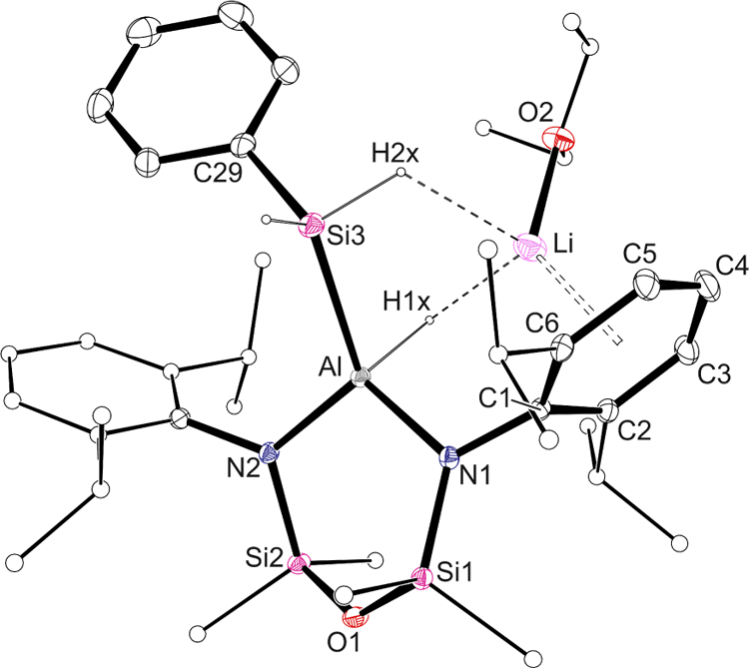
Displacement ellipsoid plot (30% probability; selected atoms represented as sticks; H atoms except those of Al*H* and Si*H*_*2*_ omitted; Li···C1–C6 centroid included; dashed lines representing Li···H interactions) of **1-Li·(Et**_**2**_**O)**.

**Figure 3:**
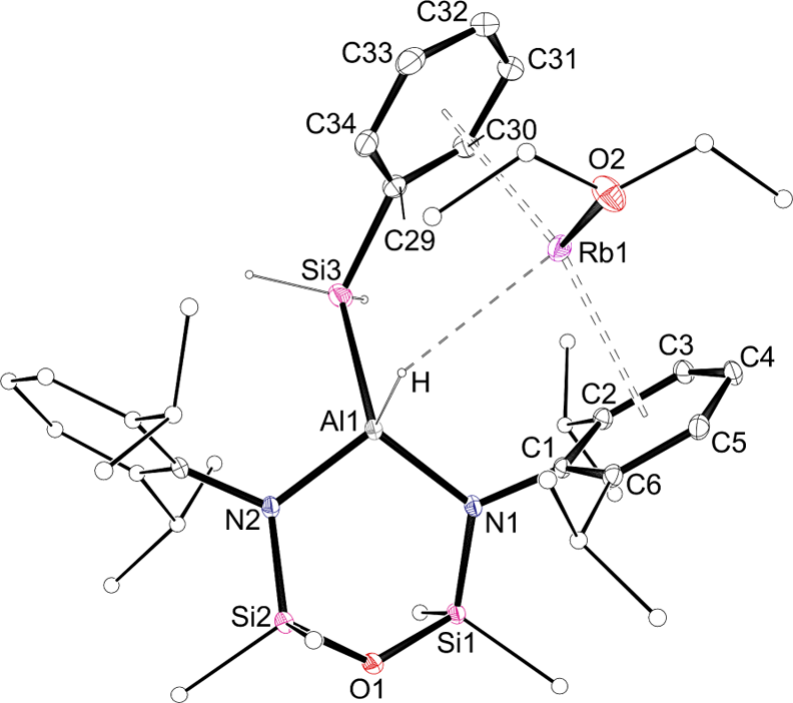
Displacement ellipsoid plot (30% probability; selected atoms represented as sticks; H atoms except those of Al*H* and Si*H*_*2*_ omitted; Rb···C1–C6 and Rb···C29–C34 centroids included; dashed line representing Rb···H interaction) of **1-Rb·(Et**_**2**_**O)**.

**Figure 4:**
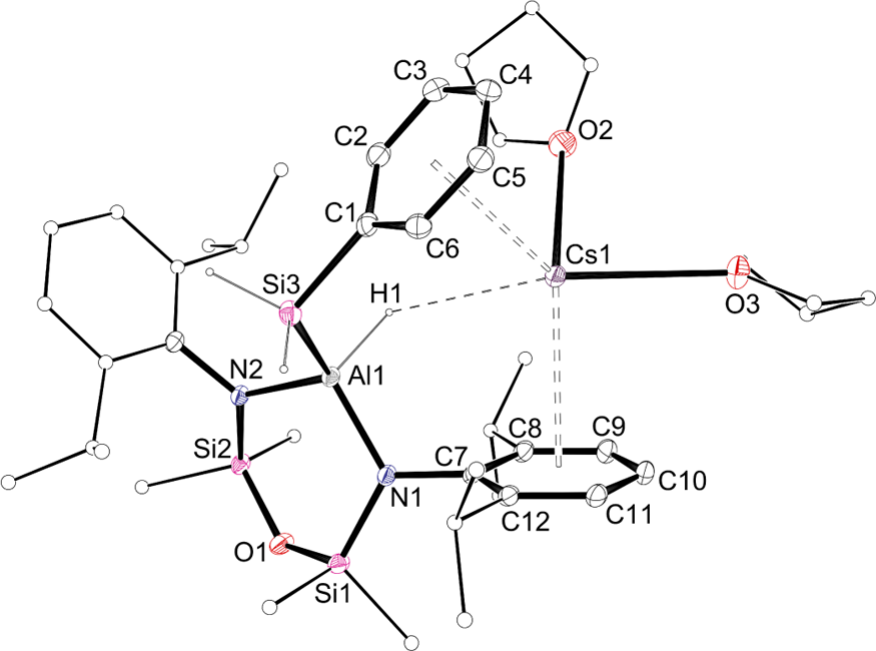
Displacement ellipsoid plot (30% probability; selected atoms represented as sticks; H atoms except those of Al*H* and Si*H*_*2*_ omitted; Cs···C1–C6 and Cs···C7–C12 centroids included; dashed line representing Cs···H interaction) of **1-Cs·(THF)**_**2**_.

X-ray diffraction data were collected on crystals of **1-AM·(Et**_**2**_**O)** (**AM** = Li, Na, K, Rb) and **1-Cs·(THF)**_**2**_. Collectively, these data offer a unique opportunity to examine the influence of the alkali metal on structural parameters for a series of (silyl) (hydrido) aluminate anions. Selected bond lengths and angles are collected in Table 1, with representative structures **1-Li·(Et**_**2**_**O)**, **1-Rb·(Et**_**2**_**O),** and **1-Cs·(THF)**_**2**_ shown in Figures 2–4, respectively.

**Table 1:** Selected Bond Lengths (Å) and Angles (°) for **1-AM·(Et**_**2**_**O)** (**AM** = Li, Na, K, Rb) and **1-Cs·(THF)**_**2**_

	AM = Li	AM = Na	AM = K	AM = Rb^a^	AM = Cs^b^
Al–Si3	2.4670(5)	2.5088(4)	2.5187(5)	2.5275(6)	2.5183(6)
Al–N1	1.8752(10)	1.8542(10)	1.8633(9)	1.8808(11)	1.8747(13)
Al–N2	1.8451(10)	1.8772(9)	1.8804(9)	1.8693(11)	1.8750(13)
Al–H1x	1.62(2)	1.58(2)	1.60(2)	1.58(2)	1.56(2)
AM···H1x	2.01(2)	2.24(2)	2.26(2)	2.83(2)	3.23(2)
AM···H2x	2.43(2)				
AM–O2	1.896(3)	2.2691(10)	2.6923(10)	2.8277(12)	3.0171(14)
AM–O3					3.0501(13)
Al···AM	3.004(3)	3.3261(6)	3.7198(7)	3.8464(4)	4.1469(4)
N1–Al–N2	107.57(4)	106.89(4)	106.23(4)	105.98(5)	104.70(6)
N1–Al–Si3	116.64(3)	114.12(3)	112.29(3)	120.05(4)	117.91(5)
N2–Al–Si3	118.70(3)	118.67(3)	119.60(3)	111.42(4)	110.47(4)
Al–Si3–C*	C29: 113.46(4)	C29: 110.13(4)	C29: 110.17(4)	C29: 110.58(5)	C1: 112.71(5)

a Al = Al1, **AM** = Rb1, H1x = H1.

b **AM** = Cs1, H1x = H1.

All of the compounds in the series were isolated as molecular complexes consisting of the (silyl)(hydrido) aluminate anion [Al(NON^Dipp^)(H)(SiH_2_Ph)]^−^, which was intimately involved with ether solvated alkali metal cations, “**AM**(Et_2_O)” (**AM** = Li, Na, K, Rb) or “**AM**(THF)_2_” (**AM** = Cs). The **1-AM·(Et**_**2**_**O)** crystal structures are isomorphous (monoclinic, *P*2_1_/*c*) for **AM** = Na, K, and Rb, with the outermost members **1-Li·(Et**_**2**_**O)** and **1-Cs(THF)**_**2**_ crystallizing in the monoclinic crystal system with space groups *P*2_1_/*n* and *I*2/*a*, respectively. While we acknowledge that the precise location of hydrogen atoms cannot be determined due to the limitations of X-ray diffraction experiments, electron density consistent with the expected positions of the Al*H* and Si*H*_*2*_ hydrogen atoms were located in the electron difference map, assigned as hydrogen atoms, and freely refined. The resulting positions of these hydrogen atoms imply a combination of Al*H*···**AM**, Si*H*···**AM** interactions that support **AM**···π(arene) contacts in each structure that link the aluminate anion and alkali metal cation within each structure (vide infra).

The geometry at the aluminum atoms is best described as distorted tetrahedral defined by the chelated NON^Dipp^-ligand and the newly installed silyl and hydrido ligands. The Al–N bond lengths in **1-AM·(solvent)**_***n***_ (range: 1.8451(10) Å–1.8808(11) Å) are not significantly (within 3σ) different to those in the parent aluminyls [**AM**{Al(NON^Dipp^)}]_2_ (range: 1.8649(14) Å–1.905(3) Å) and do not allow a specific trend to be identified. We attribute this to the expected decrease in the radius upon oxidation from Al(I) to Al(III) being offset with the anticipated increase in the Al–N bond length when the coordination number rises from two to four.^19^ The N–Al–N angles reflect these similarities, with the values for **1-AM·(solvent)**_***n***_ [range: 104.70(6)–107.57(4)°] close to those observed in the starting aluminyls [range: 103.30(12)–108.13(4)°]. The Al–Si bond lengths increase regularly from **AM** = Li [Al–Si3 = 2.4670(5) Å] to **AM** = Rb [Al1–Si3 = 2.5275(6) Å], with a slight reduction for the cesium analogue **1-Cs·(THF)**_**2**_ (Al1–Si3 = 2.5183(6) Å), possibly reflecting the increased coordination number at the **AM** due to the presence of two THF ligands. These values all fall within the range noted previously for four-coordinate neutral [Al(BDI^Dipp^)(H)(SiH_2_Ph) = 2.4551(8) Å]^21^ and anionic ([**E**(H)(SiH_2_Ph)]^−^ = 2.546(3) Å),^8a^ compounds containing the Al–SiH_2_Ph ligand.

All of the variants have close contacts between the aluminum hydrido ligand and the alkali metal (Table 1), with distances less than the sum of the van der Waals radii [Σ_VdW_(Li,H) = 2.91 Å, Σ_VdW_(Na,H) = 3.37 Å, Σ_VdW_(K,H) = 3.85 Å; Σ_VdW_(Rb,H) = 4.13 Å; Σ_VdW_(Cs,H) = 4.53 Å].^22^ For the lighter alkali metals, these distances compare well with those in the dihydrido aluminate complexes (NON^Dipp^)Al(μ-H)_2_Li(Et_2_O)_2_ [Al*H*···Li = 1.95(2) Å and 1.98(2) Å] and the dimeric [**AM**{Al(NON^Dipp^)(H)_2_}]_2_ compounds [range Al*H*···Na: 2.30(2) Å–2.34(2) Å; range Al*H*···K: 2.72(2) Å–3.02(2) Å].^10^ To the best of our knowledge, however, **1-Rb·(Et**_**2**_**O)** and **1-Cs·(THF)**_**2**_ are the first structurally characterized examples of rubidium or cesium salts of (hydrido) aluminate anions, precluding meaningful comparisons.

The most prominent differences in the series of structures **1-AM·(solvent)**_***n***_ manifest in the nature and extent of the additional interactions between the **AM** cation and the anionic [Al(NON^Dipp^)(H)(SiH_2_Ph)]^−^ components. For the lightest member of the series **1-Li·(Et**_**2**_**O)** (Figure 2), the ether-solvated lithium atom is located within the π-bonding range of the aryl ring of one of the Dipp-substituents (C1–C6), with an apparent additional Si*H*···Li interaction [H2x···Li = 2.43(2) Å]. In contrast, the silyl substituent is rotated about the Al–Si bond in the sodium, potassium, and rubidium congeners (e.g., **1-Rb·(Et**_**2**_**O)** (Figure 3)) such that the Et_2_O solvated **AM** is located between two C_6_-aryl rings, one from a Dipp substituent (C1–C6) and the other belonging to the Si*Ph* group (C29–C34). A similar orientation of the silyl substituent in **1-Cs·(THF)**_**2**_ (Figure 4) also locates the solvated **AM** between two six-membered rings belonging to a Dipp substituent (C7–C12) and the Si*Ph* group (C1–C6). This likely reflects a decrease in the tendency of the lithium to engage in Li···π(arene) interactions when compared with the larger (softer) alkali metals.

**Figure 5:**
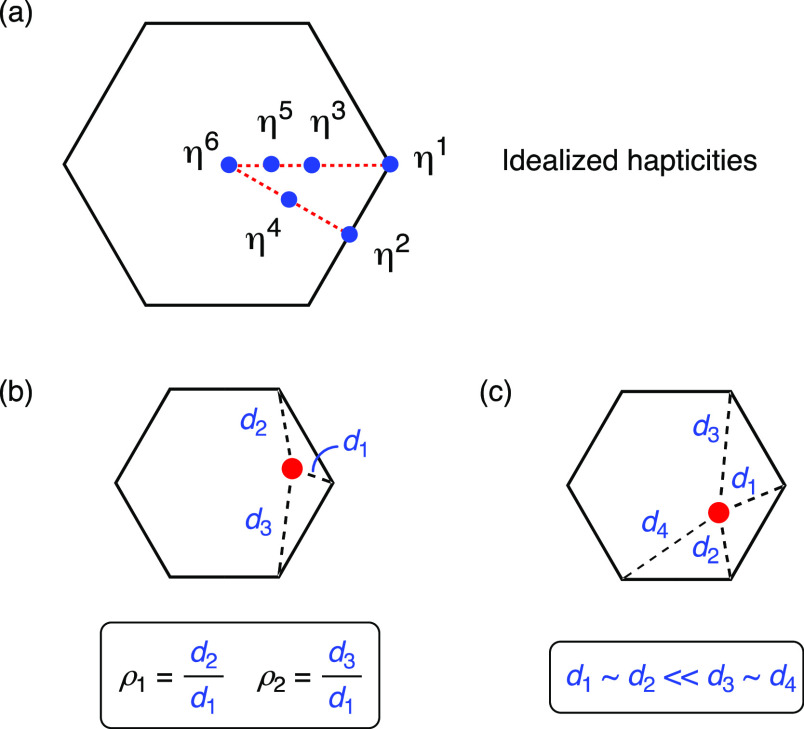
(a) Plan projection of the idealized positions according to η^*n*^-hapticities for *n* = 1–6; (b) definition of ρ_1_ and ρ_2_ according to the three shortest metal···C distances in **AM**···π(arene) complexes; (c) idealized η^4^-hapticity, introducing Δ*d*_4–3_ (= *d*_4_–*d*_3_).

We have examined the extent to which the **AM** interacts with the C_6_ rings for each of the complexes described in this study. In addition to basic evaluations involving distance and angle measurements to the centroid (Ct) of the C_6_-ring, we have used the criteria established by Alvarez and co-workers to quantify low hapticity (η^1^–η^3^) aryl interactions (Figure 5a).^23^ We are mindful of the difference between the transition metal systems examined previously that involved arguments concerning d-orbital occupancy and the current s-block metal complexes and have therefore used the established principles for geometric analysis and not an explanation of *why* these various hapticities are present. The Alvarez criteria define ρ_1_ as the ratio of *d*_2_/*d*_1_ and ρ_2_ as the ratio of *d*_3_/*d*_1_, where *d*_*n*_ is the distance from the carbon to the **AM**, and *d*_1_ < *d*_2_ < *d*_3_ < *d*_4_ (Figure 5b). Using these relationships, an η^1^-hapticity is consistent with ρ_1_ ∼ ρ_2_ ≫ 1; η^2^-hapticity best fits the situation when *d*_1_ ∼ *d*_2_ < *d*_3_ and ρ_2_ > ρ_1_ ∼ 1, and for η^3^-hapticity, *d*_1_ ∼ *d*_2_ ∼ *d*_3_ and ρ_2_ ∼ ρ_2_ ∼ 1. Extending these definitions to higher hapticities according to the idealized positions, we include a simple measure of Δ*d*_4–3_ = *d*_4_–*d*_3_, which would approach zero for idealized η^4^-hapticity (Figure 5c). We have attempted to quantify the **AM**···Dipp and **AM**···Ph interactions in the series **1-AM·(solvent)**_***n***_ compounds, using these criteria to map any trends related to the identity of the **AM**.

**Figure 6:**
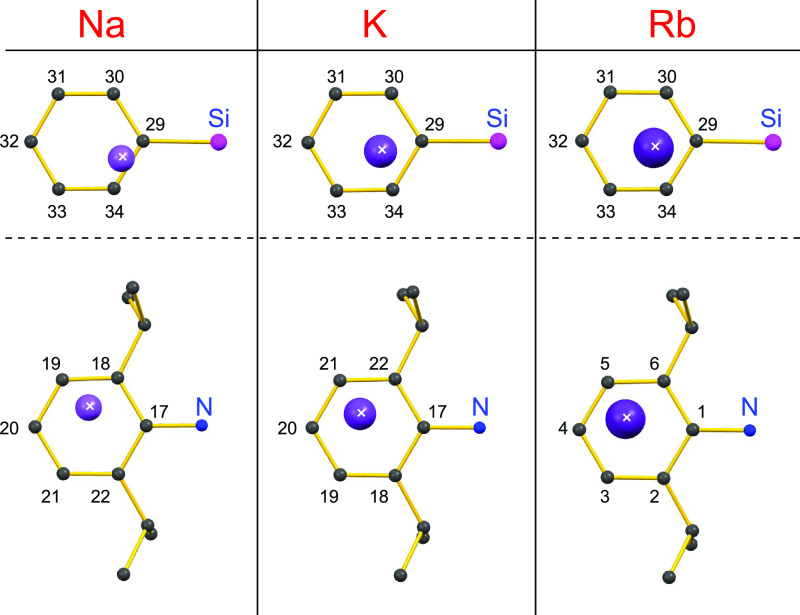
Plan view of the position of the alkali metals above the C_6_-rings of the Si*Ph* and Dipp substituents for the isomorphous compounds **1-AM·(Et**_**2**_**O)** (**AM** = Na, K, Rb). Numbers correspond to the C-atom labels from the X-ray diffraction data.

For all members of the series, we observe an increase in **AM**···Ct distance as the size of the cation increases from Li to Cs (Table 2), which coincides with an increase in the Ct···Ct distance. We also note that the plane normal-to-plane normal angle for the mean square planes defined by C_6_-rings and the Ct···**AM**···Ct angle also increase. These changes are expected as the size of the **AM** cation increases and are also consistent with what is predicted on the basis of a proposed increase in the hapticity of the arene rings as larger (softer) cations are present.^24^ To test this theory in more detail, we present the plan view of the **AM** above the C_6_-rings of the Si*Ph* and Dipp-substituents for the isomorphous members of the series, **1-AM·(Et**_**2**_**O)** (**AM** = Na, K, Rb) in Figure 6.

**Table 2:** Selected Bond Lengths (Å) and Angles (°) for **AM** Interactions with the C_6_-Rings in **1-AM·(Et**_**2**_**O)** (**AM** = Li, Na, K, Rb) and **1-Cs·(THF)**_**2**_

	AM = Li	AM = Na	AM = K	AM = Rb	AM = Cs
Ct_(Ph)_		2.9477(8)	2.9722(7)	3.0416(8)	3.356(1)
Ct_(Dipp)_	2.197(3)	2.7144(7)	2.9135(6)	3.0078(7)	3.2852(9)
Ct···Ct (distance)		5.0335(9)	5.3067(8)	5.5037(9)	6.0513(9)
C_6_···C_6_ (angle)^a^		43.46(5)	45.21(4)	45.50(5)	51.18(6)
Ct···AM···Ct (angle)		125.44(2)	128.74(2)	130.995(2)	131.35(2)

a Plane normal-to-plane normal angle for mean square planes defined by C_6_-rings.

Visual inspection of the sodium derivative clearly indicates an η^2^-coordination to C29 and C34 of the phenyl group, supported by the calculated ρ_2_ (1.13) > ρ_1_ (1.00) (Table 3). However, this coordination mode is not as clearly defined in the heavier K and Rb derivatives, where the reduced ρ_2_ values (K = 1.06, Rb = 1.07) and relative decrease in the **AM**···C30 distance indicate a shift toward an η^3^-coordination. Indeed, it may be argued that the coordination is better described as η^4^- for **1-K·(Et**_**2**_**O)** and **1-Rb·(Et**_**2**_**O)** (Figure 5c), including the Δ*d*_4–3_ values (K = 0.02, Rb = 0.05), which show that the **AM**···C33 distances are only marginally greater than the corresponding **AM**···C30 values.

**Table 3:** Selected Bond Lengths (Å) and Angles (°) for **AM** Interactions with the C_6_-Rings in **1-AM·(Et**_**2**_**O)** (**AM** = Li, Na, K, Rb) and **1-Cs·(THF)**_**2**_

AM = Li	AM = Na	AM = K	AM = Rb	AM = Cs
Ph				
	C29: 2.861(1) ***d***_**1**_	C29: 3.101(1) ***d***_**2**_	C29: 3.199(2) ***d***_**1**_	C7: 3.602(2)
	C30: 3.232(2) ***d***_**3**_	C30: 3.271(1) ***d***_**3**_	C30: 3.325(2) ***d***_**3**_	C8: 3.538(2)
	C31: 3.599(2)	C31: 3.447(2)	C31: 3.472(2)	C9: 3.508(2)
	C32: 3.626(2)	C32: 3.461(2)	C32: 3.493(2)	C10: 3.556(2)
	C33: 3.284(2)	C33: 3.300(1) (***d***_**4**_)	C33: 3.373(2) (***d***_**4**_)	C11: 3.602(2)
	C34: 2.871(1) ***d***_**2**_	C34: 3.097(1) ***d***_**1**_	C34: 3.206(2) ***d***_**2**_	C12: 3.620(2)
	ρ_1_ = 1.00	ρ_1_ = 1.00	ρ_1_ = 1.00	
	ρ_2_ = 1.13	ρ_2_ = 1.06	ρ_2_ = 1.07	
Δ*d*_4–3_		0.02	0.05	
Dipp				
C1: 2.483(3)	C17: 3.018(1) ***d***_**3**_	C17: 3.292(1) (***d***_**4**_)	C1: 3.394(2)	C1: 3.491(2)
C2: 2.538(3)	C18: 2.887(1) ***d***_**2**_	C18: 3.428(1)	C2: 3.482(2)	C2: 3.502(2)
C3: 2.625(3)	C19: 2.882(1) ***d***_**1**_	C19: 3.313(1)	C3: 3.358(1) (***d***_**4**_)	C3: 3.663(2)
C4: 2.688(3)	C20: 3.035(1) (***d***_**4**_)	C20: 3.128(1) ***d***_**2**_	C4: 3.200(1) ***d***_**2**_	C4: 3.767(2)
C5: 2.687(3)	C21: 3.229(1)	C21: 3.068(1) ***d***_**1**_	C5: 3.180(1) ***d***_**1**_	C5: 3.751(2)
C6: 2.613(3)	C22: 3.259(2)	C22: 3.158(1) ***d***_**3**_	C6: 3.288(2) ***d***_**3**_	C6: 3.617(2)
	ρ_1_ = 1.00	ρ_1_ = 1.02	ρ_1_ = 1.01	
	ρ_2_ = 1.05	ρ_2_ = 1.03	ρ_2_ = 1.02	
Δ*d*_4–3_		0.13	0.07	

Similar arguments may be presented for the hapticity of the C_6_-ring of the Dipp substituent, where a larger difference in the ρ_2_ versus ρ_1_ value for **1-Na·(Et**_**2**_**O)** agrees with a shift to higher hapticities for the heavier congeners (Table 3). The larger Δ*d*_4–3_ value for **1-K·(Et**_**2**_**O)** 0.13 best fits the η^3^-coordination, whereas the Δ*d*_4–3_ value of 0.07 for **1-Rb·(Et**_**2**_**O)** indicates a shift toward η^4^-hapticity. These data therefore support the hypothesis that within an isomorphous series of compounds, an increase in hapticity is observed as the size of the alkali metal increases.

**Figure 7:**
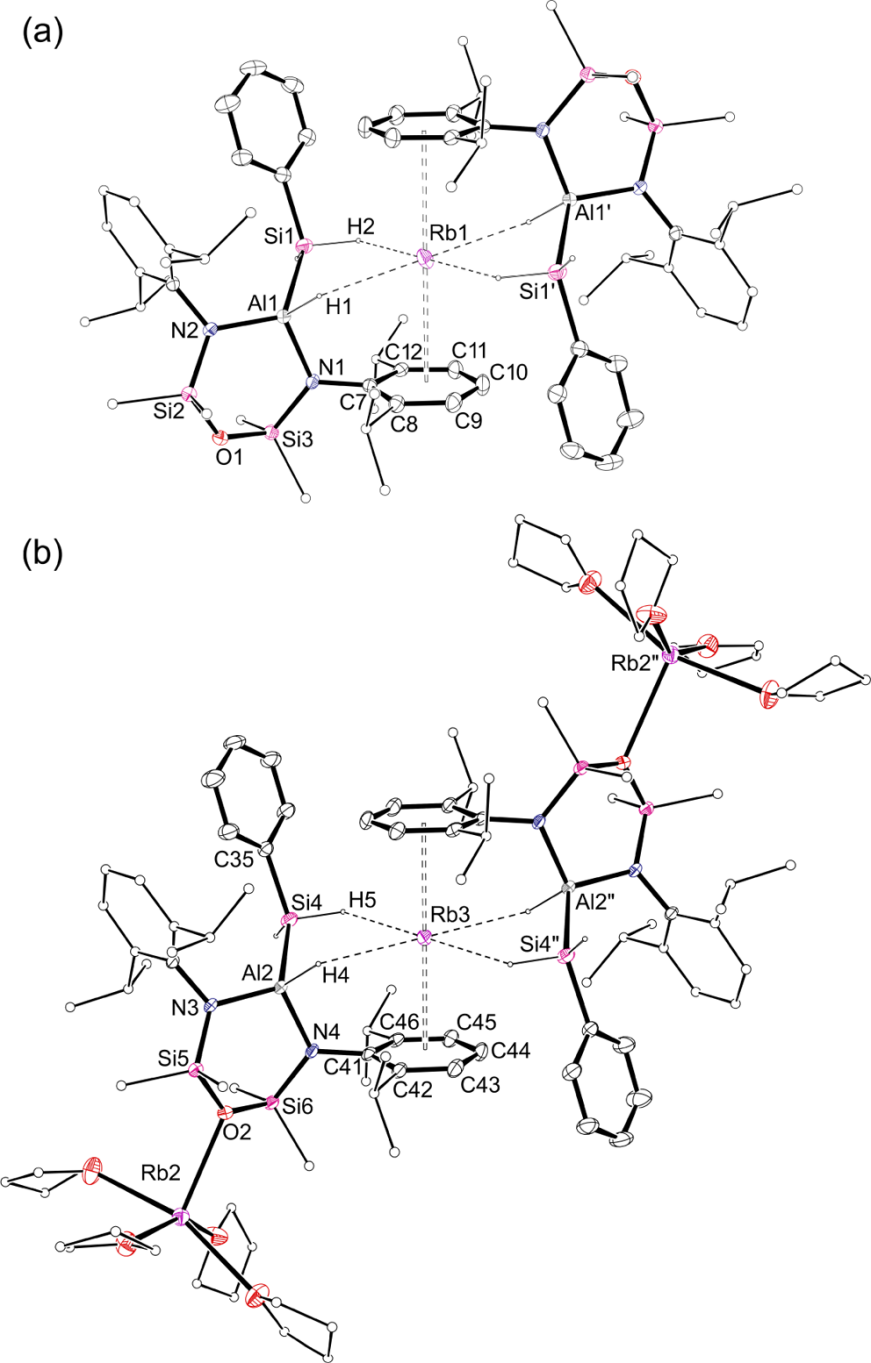
Displacement ellipsoid plot (30% probability; selected atoms represented as sticks; H atoms except those of Al*H* and Si*H*_*2*_ omitted; Rb1···C7–C12 and Rb2···C41–C46 centroids included; dashed lines representing Rb···H interactions) of compound **2** (′ 2 – *x*, −*y*, 1 – *z*; ″ = 1 – *x*, 2 – *y*, −*z*). (a) [Rb{Al(NON^Dipp^)(H)(SiH_2_Ph)}_2_]^−^ anion; (b) [{Rb(THF)_4_}_2_(Rb{Al(NON^Dipp^)(H)(SiH_2_Ph)}_2_)]^+^ cation.

**Scheme 3:**
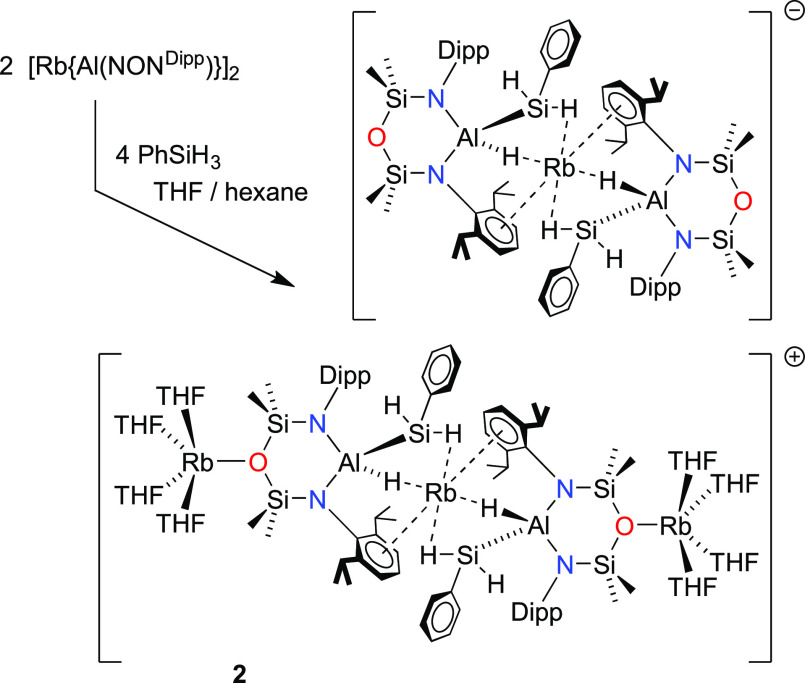
Fortuitous Synthesis of Compound **2**

When a crude reaction mixture containing Rb[Al(NON^Dipp^)(H)(SiH_2_Ph)] was purified by crystallization from a layered hexane/THF mixture, an unexpected product was obtained (Scheme 3). Analytical data suggested the formation of the rubidium analogue of **1-Cs·(THF)**_**2**_, with NMR and IR spectroscopic data consistent with the [Al(NON^Dipp^)(H)(SiH_2_Ph)]^−^ anion and elemental analysis consistent with [Rb(THF)_2_][Al(NON^Dipp^)(H)(SiH_2_Ph)]. However, analysis by single crystal X-ray diffraction revealed a remarkably different structure: a polymer consisting of the heteroleptic tris-rubidium bis-aluminum cation [{Rb(THF)_4_}_2_(Rb{Al(NON^Dipp^)(H)(SiH_2_Ph)}_2_)]^+^ and the non-solvated heteroleptic [Rb{Al(NON^Dipp^)(H)(SiH_2_Ph)}_2_]^−^ anion (**2**, Table 4, Figure 7). Compound **2** therefore represents a unique example of a rubidium rubidiate, where rubidium is present in both the cationic and anionic moieties, extending the series of known lithium lithiates,^25^ sodium sodiates,^26^ and potassium potassiates^27^ to the next member of the Group 1 metals.

**Table 4:** Selected Bond Lengths (Å) and Angles (°) for **2**^b^

	anion^a^	cation^a^
Al–Si	2.5071(10)	2.5032(9)
Al–N^c^	1.876(2)	1.872(2)
Al–N^d^	1.867(2)	1.885(2)
Al–H^e^	1.56(2)	1.57(3)
Rb···H^e^	2.78(2)	2.87(3)
Rb···H^f^	2.98(3)	3.08(3)
Rb2–O2		2.9386(16)
Al···Rb	3.8173(7)	3.7918(7)
N–Al–N^c^^,^^d^	107.30(9)	106.14(9)
N–Al–Si^c^	109.15(7)	117.95(7)
N–Al–Si^d^	118.81(7)	108.13(7)
Al–Si–C^g^	122.56(8)	124.37(8)

a Al = Al1; Si = Si1; Rb = Rb1.

b Al = Al2; Si = Si4; Rb = Rb3.

c anion, N = N1; cation, N = N3.

d anion, N = N2; cation, N = N4.

e anion, H = H1; cation, H = H4.

f anion, H = H2; cation, H = H5.

g anion, C = C1; cation, C = C35.

In contrast to **1–Rb·(Et**_**2**_**O)**, the orientation of the silyl ligand in both the cationic and anionic moieties of **2** most closely resembles that noted in the lithium complex **1-Li·(Et**_**2**_**O)**, precluding any Rb···Ph interactions. In both the cation and anion, the Rb atoms are therefore stabilized by Al*H*···Rb [2.78(2) Å and 2.87(3) Å] and Si*H*···Rb [2.98(3) Å and 3.08(3) Å] contacts, with additional Rb···π(arene) contacts to one of the Dipp substituents. As both Rb1 and Rb3 are located on an inversion center, these atoms each engage in a total of 4 × Rb···H and 2 × Rb···π(arene) interactions, with no direct bonding to the THF. However, in the cationic moiety, the outer THF-solvated Rb cations also bond to the O-atom of the adjacent NON^Dipp^-ligands, to propagate the polymeric cation–anion arrangement of **2**. The interaction of the oxygen atom with additional cations is not common for the NON^R^ligand,^28^ with the oxygen typically adopting a purely structural role in the ligand backbone.

## Conclusions

In summary, we have been able to examine the oxidative addition of phenylsilane to a complete series of dimeric aluminyls, [**AM**{Al(NON^Dipp^)}]_2_ for **AM** = Li, Na, K, Rb, and Cs. Although we did not observe any obvious alkali metal effects in these reactions (since they all proceeded smoothly under ambient conditions), the structurally similar products allowed a systematic study of the role of **AM** in stabilizing the resulting (silyl)(hydrido) aluminate complexes. The products were crystallized from Et_2_O (Li, Na, K, Rb) or THF (Cs) to give monomeric [**AM**(Et_2_O)][Al(NON^Dipp^)(H)(SiH_2_Ph)] and [Cs(THF)_2_][Al(NON^Dipp^)(H)(SiH_2_Ph)], respectively. Crystallographic analysis shows that the solvated **AM** atoms are each supported by an Al*H*···**AM** contact, in addition to Si*H*···**AM** (**AM** = Li and Rb) and **AM**···π(arene) interactions with a Dipp substituent (Li–Cs) and the phenyl group (Na–Cs). Detailed analysis of the hapticity in an isomorphous series of Na, K, and Rb complexes indicates a tendency for the larger alkali metals to bond via an increased hapticity, a feature that can contribute toward variations in reactivity down the group. In addition, we discovered that when the Rb analogue was crystallized from a mixed hexane/THF solvent system, a novel rubidium rubidiate salt was isolated, extending the series of lithium lithiates, sodium sodiates, and potassium potassiates to the next heaviest Group 1 metal member and leaving only the cesium cesiate to be discovered.

## Notes

Data used within this manuscript can be accessed at https://doi.org/10.15129/477fd1d6-12b7-441b-a1d1-931f08ce47b8.

## Supporting Information Available

Experimental methods, crystallographic information, NMR spectra, IR data, and melting point data (PDF)

## Footnotes

a Data pertaining to the previously reported addition of phenylsilane to the potassium aluminyl [K{Al(NON^Dipp^)}]_2_ have been included in this discussion to facilitate the mapping of trends within the full series of aluminyl compounds.

b Unfortunately, the sensitivity of **1-Li·(Et**_**2**_**O)** towards air and moisture precluded the collection of any meaningful IR data due to rapid decomposition. A representative spectrum is included in the Supporting Information (Figure S74).
