# Supplementary material for: Synthesis, Characterization, and Structural Analysis of AM[Al(NONDipp)(H)(SiH2Ph)] (AM = Li, Na, K, Rb, Cs) Compounds, Made Via Oxidative Addition of Phenylsilane to Alkali Metal Aluminyls
Source: Inorg Chem. 2022 Nov 23;61(49):19838–46. doi: 10.1021/acs.inorgchem.2c03010 (PMC9749022; doi:10.1021/acs.inorgchem.2c03010)
Supplement: Supplementary file 1 — ic2c03010_si_001.pdf [file ic2c03010_si_001.pdf]

SUPPORTING INFORMATION

Synthesis, Characterisation and Structural Analysis of **AM**[Al(NON^{Dipp})(H)(SiH$_2$Ph)] (**AM** = Li, Na, K, Rb, Cs) Compounds, Made via Oxidative Addition of Phenylsilane to Alkali Metal Aluminyls

Gerd M. Ballmann,[a] Matthew J. Evans,[b] Thomas X. Gentner,[a] Alan R. Kennedy,[a] J. Robin Fulton,[b] Martyn P. Coles,*[b] Robert E. Mulvey*[a]

[a] *Department of Pure and Applied Chemistry, University of Strathclyde, 295 Cathedral St., G1 1XL Glasgow, UK. E-mail: r.e.mulvey@strath.ac.uk*

[b] *School of Chemical and Physical Sciences, Victoria University of Wellington, P.O. Box 600, Wellington, New Zealand. E-mail: martyn.coles@vuw.ac.nz*

Contents

1) General experimental procedures and complex syntheses S2

2) Crystal structure data S9

3) Selected ^{1}H-, ^{13}C-, DEPT-135-, DEPT-90-, ^{1}H-^{1}H COSY-, HSQC-,

HMBC-, ^{29}Si- and ^{27}Al- NMR spectra S19

4) Selected infrared spectra S53

5) References S55

1) General experimental procedures and complex syntheses

General experimental procedures

All manipulations were performed under dry argon or nitrogen using standard Schlenk-line techniques, or in a conventional nitrogen-filled glovebox. Starting materials and research chemicals were obtained from commercial suppliers where appropriate and used without further purification. Al(NON^{Dipp})I,[S1] [Li{Al(NON^{Dipp})}]$_2$,[S2] [Na{Al(NON^{Dipp})}]$_2$,[S2] RbC_8[S3] and CsC_8[S3] were synthesized according to literature procedures. The literature known methodology for the synthesis of [Rb{Al(NON^{Dipp})}]$_2$[S4] and [Cs{Al(NON^{Dipp})}]$_2$.[S4] was followed but instead of aromatic solvents the reaction was conducted in methylcyclohexane. The preparation and analytical data for compound **1-K·(Et$_2$O)** has been reported previously.[S5]

<u>For compounds prepared at Victoria University of Wellington: **1-Li·(Et$_2$O)** and **1-Na·(Et$_2$O)**</u>

Hexane, toluene, diethyl ether (Et_2O), and tetrahydrofuran (THF) were obtained from a PureSolv MD 5 system and stored over activated 5Å molecular sieves for 24 hours prior to use. NMR spectra were recorded using a Jeol JNM-ECZ500S 500 MHz (11.747 Tesla) spectrometer equipped with a ROYAL digital auto tune probe S, operating at 500.1 (^{1}H), 194.4 (^{7}Li), 202.4 (^{31}P), 130.3 (^{27}Al), 125.8 (^{13}C) and 99.3 (^{29}Si) MHz. Spectra were recorded at 294 K (unless stated otherwise) and proton and carbon chemical shifts were referenced internally to residual solvent resonances. Coupling constants are quoted in Hz. Elemental analyses were performed by the Elemental Analysis Service at London Metropolitan University. Fourier transform infrared (FT-IR) spectra were recorded on solid samples, using a Bruker Tensor 27 FT-IR spectrometer. Melting points of selected products and starting materials were determined by loading a small sample into a glass capillary in the glove box, sealing the tube with Blu Tack®, and then heating the sample up to 260 ℃ in an SRS digimelt (MPA160) apparatus. Crystallographic data for complexes **1-Li·(Et$_2$O)** and **1-Na·(Et$_2$O)** were collected an Agilent SuperNova diffractometer fitted with an EOS S2 detector. Crystals were covered in inert oil and mounted. Data were collected at the temperature indicated (120 K or 150 K)[S6] using focused micro-source Cu Kα radiation at 1.54184 Å. Intensities were corrected for Lorentz and polarisation effects and for absorption using multi-scan methods.[S7] Space groups were determined from systematic absences and checked for higher symmetry. All structures were solved using direct methods with SHELXS,[S8] refined on F^2 using all data by full matrix least-squares procedures with SHELXL-97,[S9,S10] within the WinGX13 program. Non-hydrogen atoms were refined with anisotropic displacement parameters. Hydrogen atoms were placed in calculated positions or manually assigned from residual electron density where appropriate, unless otherwise stated. The functions minimized were $\Sigma w(F^2_{obs} - F^2_{calc})$, with $w = [\sigma^2(F^2_{obs})$

+ aP^2 + bP]-1, where P = [max(F_{obs})^2 + 2F^2_{calc}]/3. The isotropic displacement parameters are 1.2 or 1.5 times the isotropic equivalent of their carrier atoms.

For compounds prepared at the University of Strathclyde: **1-Rb·(Et_2O)**, **1-Cs·(THF)$_2$** and **2**

Hexane, THF and diethyl ether were dried by heating to reflux over sodium benzophenone ketyl and then distilled under nitrogen prior to use. Pentane, benzene, and toluene were degassed with nitrogen, dried over activated aluminium oxide (Innovative Technology, Pure Solv 400-4-MD, Solvent Purification System), and then stored under inert atmosphere over activated 4 Å molecular sieves. Benzene-*d$_6$*, toluene-*d$_8$* and THF-*d$_8$* were degassed by freeze-pump-thaw methods and stored over activated 4 Å molecular sieves. NMR spectra were recorded on a Bruker AV3 or AV 400 MHz spectrometer operating at 400.13 MHz for ^{1}H, 100.62 MHz for ^{13}C. All ^{13}C spectra were proton decoupled. ^{1}H and ^{13}C{^{1}H} chemical shifts are expressed in parts per million (δ, ppm) and referenced to residual solvent peaks. Coupling constants (*J*) are given in Hertz (Hz). For describing signal multiplicities common abbreviations were used: s (singlet), d (doublet), t (triplet), q (quartet), hept (heptet), m (multiplet) and br (broad). Infrared spectra of starting materials and selected products were obtained as Nujol mulls on NaCl plates. Mulls were prepared in the glove box using anhydrous Nujol, which was dried and stored over activated 4 Å molecular sieves under argon, and then transferred to the spectrometer in a desiccator. Spectra were recorded on a Nicolet 360 FTIR spectrometer spanning 4000-400 cm^{-1}. The melting points of selected products and starting materials were determined as follows. A small sample of crystalline/powdered material was loaded into a melting point tube in the glove box. This tube was then sealed with Plasticine® before removal from the glove box. The melting point was then determined in the usual manner using a Buchi Melting Point B-545 apparatus. Elemental analysis was conducted by the Elemental Analysis Service at London Metropolitan University. Crystallographic data for complexes **1-Rb·(Et_2O)** and **1-Cs·(THF)$_2$** were collected on an Oxford Diffraction Gemini S instrument with graphite-monochromated Mo−Kα (λ 0.71073 Å) radiation or on Rigaku XtaLAB Synergy-S with monochromated Cu−Kα (λ 1.54184 Å) radiation. The measured data was processed with the CrysAlisPro[S11] software package. Using Olex2,[S12] the structure was solved with the ShelXT[S8] structure solution program using Intrinsic Phasing and refined with the ShelXL[S13] refinement package using Least Squares minimization or by the full-matrix least-squares method using SHELXL-2018 implemented within WINGX.[S8] All non-hydrogen atoms were refined using anisotropic thermal parameters unless noted otherwise.

Complex syntheses

Preparation of [Li(Et_2O)][Al(NON^{Dipp})(H)(SiH_2Ph)]: 1-Li·(Et_2O)

A solution of [Li{Al(NON^{Dipp})}]$_2$ (66 mg, 0.06 mmol) in diethyl ether was added to a solution of phenylsilane (14 mg, 0.06 mmol) in diethyl ether and stirred for *ca.* 1 hour to give a colourless solution. Crystallisation was achieved at room temperature via. slow evaporation from a diethyl ether solution. Yield 80 mg, 90 %.

^{1}H NMR (500 MHz, THF-d_8): δ 6.88 – 6.83 (m, 5H, Ar-C*H*), 6.73 (d, *J* = 7.6 Hz, 2H, Ar-C*H*), 6.72 – 6.69 (m, 2H, Ar-C*H*), 6.68 – 6.64 (m, 2H, Ar-C*H*), 4.17 (hept, *J* = 6.8 Hz, 2H, C*H*(CH_3)$_2$), 4.09 (hept, *J* = 6.8 Hz, 2H, C*H*(CH_3)$_2$), 3.38 (q, *J* = 7.0 Hz, 4H, Et_2O), 3.18 (d, *J* = 3.0 Hz, 2H, SiH_2), 1.16 (d, *J* = 6.8 Hz, 6H, CH(CH_3)$_2$), 1.14 – 1.09 (m, 24H, CH(CH_3)$_2$, Et_2O), 0.11 (s, 6H, Si(CH_3)$_2$), 0.04 (s, 6H, Si(CH_3)$_2$).

^{13}C{^{1}H} NMR (126 MHz, THF-d_8): δ 148.1, 147.4, 138.0, 126.6, 125.5, 123.8, 123.2, 121.4 (C_6H_3, C_6H_5), 66.4 (Et_2O), 27.8, 27.7 (*C*H(CH_3)$_2$), 26.8, 26.7, 26.3, 26.2 (CH(*C*H_3)$_2$), 15.8 (Et_2O), 3.3, 2.8 (Si(*C*H_3)$_2$).

^{1}H NMR (500 MHz, C_6D_6): δ 7.08 – 6.99 (m, 4H, Ar-C*H*), 6.96 – 6.87 (m, 5H, Ar-C*H*), 6.85 – 6.80 (m, 2H, Ar-C*H*), 4.10 (hept, *J* = 6.8 Hz, 2H, C*H*(CH_3)$_2$), 4.04 (hept, *J* = 6.8 Hz, 2H, C*H*(CH_3)$_2$), 3.67 (d, *J* = 3.6 Hz, 2H, SiH_2), 3.18 (q, *J* = 7.0 Hz, 32H, Et_2O),* 1.38 (d, *J* = 6.8 Hz, 7H, CH(CH_3)$_2$), 1.26 (d, *J* = 6.8 Hz, 6H, CH(CH_3)$_2$), 1.25 (d, *J* = 6.8 Hz, 6H, CH(CH_3)$_2$), 1.20 (d, *J* = 6.8 Hz, 6H, CH(CH_3)$_2$), 0.98 (t, *J* = 7.0 Hz, 42H, Et_2O),* 0.49 (s, 6H, Si(CH_3)$_2$), 0.32 (s, 6H, Si(CH_3)$_2$).

^{13}C{^{1}H} NMR (126 MHz, C_6D_6): δ 148.3, 147.6, 145.9, 137.6, 127.4, 127.3, 124.5, 124.4, 124.2, 123.2 (C_6H_3, C_6H_5), 65.8 (Et_2O), 28.1, 27.9 (*C*H(CH_3)$_2$), 26.2, 25.7, 25.7, 25.2 (CH(*C*H_3)$_2$), 15.3 (Et_2O), 3.1, 2.6 (Si(*C*H_3)$_2$).

^{29}Si{^{1}H} NMR (99 MHz, THF-d_8): δ –11.98 (*Si*(CH_3)$_2$). ^{29}Si{^{1}H} NMR (99 MHz, C_6D_6): δ –7.62 (*Si*(CH_3)$_2$). Signal for Al-*Si*H_2Ph not detected.

^{7}Li{^{1}H} NMR (194 MHz, THF-d_8): δ 2.72. ^{7}Li{^{1}H} NMR (194 MHz, C_6D_6): δ 2.41.

^{27}Al (130 MHz, THF-d_8): δ 127. ^{27}Al (130 MHz, C_6D_6): δ 123.

^{27}Al{^{1}H} (130 MHz, THF-d_8): δ 125. ^{27}Al{^{1}H} (130 MHz, C_6D_6): δ 122.

m.p. > 260 °C.

*Upon addition of C_6D_6 to **1-Li·(Et_2O)**, a large quantity of insoluble white precipitate was formed in the NMR tube. This suggested that some of the material underwent desolvation, while a small quantity of the etherate remained in solution. The C_6D_6 NMR sample was heated to 343 K to acquire a meaningful dataset.

Despite attempts to acquire a suitable elemental analyses and IR spectrum, the sensitivity of the compound towards air and moisture precluded the collection of any meaningful data (an example spectrum is provided).

Preparation of [Na(Et$_2$O)][Al(NON^{Dipp})(H)(SiH$_2$Ph)]: 1-Na·(Et$_2$O)

A solution of [Na{Al(NON^{Dipp})}]$_2$ (64 mg, 0.06 mmol) in diethyl ether was added to a solution of phenylsilane (13 mg, 0.06 mmol) in diethyl ether and stirred for *ca.* 1 hour to give a colourless solution. Crystallisation was achieved at room temperature *via.* slow evaporation from a diethyl ether solution. Yield 78 mg, 91 %.

^{1}H NMR (500 MHz, C$_6$D$_6$): δ 7.08 – 6.93 (m, 1H, Ar-C*H*), 6.97 – 6.88 (m, 1H, Ar-C*H*), 6.85 (t, *J* = 7.6 Hz, 2H, Ar-C*H*), 4.18 (hept, J = 6.8 Hz, 2H, C*H*(CH$_3$)$_2$), 4.11 (hept, *J* = 6.8 Hz, 2H, C*H*(CH$_3$)$_2$), 3.44 (s, 2H, Si*H$_2$*), 3.24 (q, *J* = 7.0 Hz, 4H, Et$_2$O), 1.40 (d, *J* = 6.8 Hz, 6H, CH(C*H$_3$*)$_2$), 1.33 (d, *J* = 6.8 Hz, 6H, CH(C*H$_3$*)$_2$), 1.27 (d, *J* = 6.8 Hz, 6H, CH(C*H$_3$*)$_2$), 1.27 (d, *J* = 6.8 Hz, 6H, CH(C*H$_3$*)$_2$), 1.24 (br t, 6H, Et$_2$O), 0.56 (s, 6H, Si(C*H$_3$*)$_2$), 0.43 (s, 6H, Si(C*H$_3$*)$_2$).

^{13}C{^{1}H} NMR (126 MHz, C$_6$D$_6$): δ 148.6, 148.3, 146.8, 137.5, 127.5, 123.9, 123.7, 122.4 (C$_6$H$_3$, *C$_6$*H$_5$), 65.8 (Et$_2$O), 28.0, 27.5 (*C*H(CH$_3$)$_2$), 26.3, 25.9, 25.7, 24.9 (CH(*C*H$_3$)$_2$), 15.4 (Et$_2$O), 2.8, 2.7 (Si(*C*H$_3$)$_2$).

^{29}Si{^{1}H} NMR (99 MHz, C$_6$D$_6$): δ –9.14 (Si(*C*H$_3$)$_2$). Signal for Al-*Si*H$_2$Ph not detected.

^{27}Al (130 MHz, C$_6$D$_6$): δ 125.

^{27}Al{^{1}H} (130 MHz, C$_6$D$_6$): δ 124.

m.p. 240 – 245 °C (dec.).

Elemental analysis: Calculated values for C$_{38}$H$_{64}$AlN$_2$NaO$_2$Si$_3$ (717.17 g/mol): C 63.82, H 9.02, N 3.92; Found: C 63.27, H 9.19, N 3.56.

IR (solid, cm^{-1}): 1\λ 2962 (m), 2869 (w), 2084 (w), 2056 (w), 1648 (w), 1461 (w), 1432 (w), 1421 (w), 1380 (w), 1360 (w), 1314 (w), 1248 (m), 1238 (s), 1190 (m), 1106 (w), 1052 (w), 1041 (w), 1004 (w), 961 (m), 937 (m), 918 (m).

Preparation of [Rb(Et$_2$O)][Al(NON^{Dipp})(H)(SiH$_2$Ph)]: 1-Rb·(Et$_2$O)

In a 25 mL Schlenk flask [Rb{Al(NON^{Dipp})}]$_2$ (574 mg, 0.964 mmol) was dissolved in hexane (18 mL) to give a yellow solution. PhSiH$_3$ (131 µL, 1.062 mmol, 1.10 equiv.) was added resulting in the precipitation of a white solid within one hour of stirring at room temperature. After stirring the reaction mixture for additional 16 hours at room temperature the precipitate was collected via filtration and subsequently

washed with *n*-hexane (3 x 4 mL). The crude product was dissolved in Et_2O (6 mL) and slow evaporation at room temperature yielded colourless crystals suitable for single X-ray diffraction. Isolation of the crystalline material and drying at high vacuum the target compound was obtained as white solid in 78 % (587 mg, 0.755 mmol) yield.

^{1}H NMR (400 MHz, C_6D_6:THF-d_8 (10:1), 25 °C): δ 7.11 – 7.03 (m, 4H, Ar-C*H*), 7.02 – 6.99 (m, 2H, Ar-C*H*), 6.96 – 6.87 (m, 5H, Ar-C*H*), 4.38 (hept, *J* = 6.8 Hz, 2H, C*H*($CH_3)_2$), 4.26 (hept, *J* = 6.6 Hz, 2H, C*H*($CH_3)_2$), 3.72 (d, *J* = 3.1 Hz, 2H, SiH_2Ph), 3.26 (q, *J* = 6.8 Hz, ($H_3CCH_2)_2O$), 1.45 – 1.24 (m, 24H, CH($CH_3)_2$), 1.08 (t, *J* = 7.0 Hz, ($H_3CCH_2)_2O$), 0.54 (s, 6H, Si($CH_3)_2$), 0.41 (s, 6H, Si($CH_3)_2$).

$^{13}C\{^{1}H\}$ NMR (101 MHz, C_6D_6:THF-d_8 (10:1), 25 °C): δ 148.3 (C_{arom}), 148.2 (C_{arom}), 148.0 (C_{arom}), 139.9 (C_{arom}), 137.6 (C_{arom}H), 127.2 (C_{arom}H), 126.5 (C_{arom}H), 124.0 (C_{arom}H), 123.9 (C_{arom}H), 122.1 (C_{arom}H), 65.9 ($C_{ether}H_2$), 27.7 (C_{aliph}H), 27.6 (C_{aliph}H), 26.4 ($C_{aliph}H_3$), 26.1 ($C_{aliph}H_3$), 25.8 ($C_{aliph}H_3$), 25.5 ($C_{aliph}H_3$), 15.5 ($C_{ether}H_2$), 3.1 (Si($CH_3)_2$), 2.7 (Si($CH_3)_2$).

^{27}Al NMR (104 MHz, C_6D_6:THF-d_8 (10:1), 25 °C): δ 121.

^{29}Si NMR (80 MHz, C_6D_6:THF-d_8 (10:1), 25 °C): δ –10.2 (*Si*($CH_3)_2$). Signal for Al-*Si*H_2Ph not detected.

m.p.: 179 °C – 180 °C (dec.)

A satisfactory elemental analysis for the bulk material of **1-Rb·(Et_2O)** was not obtained, which may be attributed to decomposition during shipping and/or sample preparation. Obtained values are given, nevertheless. Elemental analysis: Calculated values for $C_{38}H_{64}AlN_2O_2RbSi_3$ (777.65 g/mol): C 58.69, H 8.30, N 3.60; Found: C 52.62, H 8.22, N 2.78.

IR (Nujol, cm^{-1}): 1\λ 2072 (w), 2056 (w), 1678 (w), 1424 (w), 1310 (w), 1247 (m), 1189 (m), 1100 (w), 1041 (w), 1007 (m), 938 (m), 923 (m), 846 (w), 796 (w), 772 (w), 723 (w), 671 (w).

Preparation of [Cs(THF)$_2$][Al(NON^{Dipp})(H)(SiH$_2$Ph)]: 1-Cs·(THF)$_2$

In a 25 mL Schlenk flask [Cs{Al(NON^{Dipp})}]$_2$ (378 mg, 0.588 mmol) was dissolved in hexane (15 mL) to give a yellow solution. $PhSiH_3$ (90 µL, 0.729 mmol, 1.24 equiv.) was added resulting in the precipitation of a white solid within 10 minutes of stirring at room temperature. After stirring the reaction mixture for additional 16 hours at room temperature the precipitate was collected via filtration and subsequently washed with *n*-hexane (4 x 2 mL). The crude product was then dissolved in THF (4 mL) and layered with n-hexane. Storing this solution at -20 °C for two days yielded colourless crystals suitable for single X-ray diffraction. Isolation of the crystalline material and drying at high vacuum the target compound was obtained as white solid in 64 % (336 mg, 0.375 mmol) yield.

^{1}H NMR (400 MHz, THF-d_8, 25 °C): δ 7.00 – 6.92 (m, 4H, Ar-C*H*), 6.89 – 6.75 (m, 7H, Ar-C*H*), 4.15 (hept, *J* = 6.8 Hz, 2H, C*H*(CH_3)$_2$), 4.03 (hept, *J* = 6.8 Hz, 2H, C*H*(CH_3)$_2$), 3.64 – 3.59 (m, THF), 3.37 (d, *J* = 3.1 Hz, 2H, SiH_2Ph), 1.80 – 1.75 (m, THF), 1.24 – 1.09 (m, 24H, CH(CH_3)$_2$), 0.16 (s, 6H, Si(CH_3)$_2$), 0.06 (s, 6H, Si(CH_3)$_2$).

^{1}H NMR (400 MHz, C_6D_6:THF-d_8 (4:1), 25 °C): δ 7.06 – 6.93 (m, 6H, Ar-C*H*), 6.91 – 6.81 (m, 5H, Ar-C*H*), 4.29 (hept, *J* = 6.7 Hz, 2H, C*H*(CH_3)$_2$), 4.17 (hept, *J* = 6.7 Hz, 2H, C*H*(CH_3)$_2$), 3.62 (s, 2H, SiH_2Ph), 3.56 – 3.49 (m, THF), 1.55 – 1.48 (m, THF), 1.37 – 1.22 (m, 24H, CH(CH_3)$_2$), 0.41 (s, 6H, Si(CH_3)$_2$), 0.27 (s, 6H, Si(CH_3)$_2$).

$^{13}C\{^{1}H\}$ NMR (101 MHz, THF-d_8, 25 °C): δ 148.6 (C_{arom}), 148.6 (C_{arom}), 148.3 (C_{arom}), 141.3 (C_{arom}), 138.1 (C_{arom}H), 127.4 (C_{arom}H), 126.6 (C_{arom}H), 124.5 (C_{arom}H), 124.1 (C_{arom}H), 122.4 (C_{arom}H), 68.4 ($C_{THF}H_2$), 28.1 (C_{aliph}H), 28.0 (C_{aliph}H), 26.7 ($C_{aliph}H_3$), 26.6 ($C_{aliph}H_3$), 26.4 ($C_{aliph}H_3$), 26.3 ($C_{aliph}H_3$), 26.1 ($C_{THF}H_2$), 3.2 (Si(CH_3)$_2$), 2.8 (Si(CH_3)$_2$).

$^{13}C\{^{1}H\}$ NMR (101 MHz, C_6D_6:THF-d_8 (4:1), 25 °C): δ 148.2 (C_{arom}), 148.2 (C_{arom}), 140.9 (C_{arom}), 137.6 (C_{arom}H), 127.2 (C_{arom}H), 126.4 (C_{arom}H), 124.2 (C_{arom}H), 123.9 (C_{arom}H), 122.2 (C_{arom}H), 67.8 ($C_{THF}H_2$), 27.7 (C_{aliph}H), 27.5 (C_{aliph}H), 26.2 ($C_{aliph}H_3$), 26.1 ($C_{aliph}H_3$), 25.9 ($C_{THF}H_2$), 25.7 ($C_{aliph}H_3$), 25.6 ($C_{aliph}H_3$), 3.0 (Si(CH_3)$_2$), 2.5 (Si(CH_3)$_2$).

^{27}Al NMR (104 MHz, THF-d_8, 25 °C): δ 121.

^{27}Al NMR (104 MHz, C_6D_6:THF-d_8 (4:1), 25 °C): δ 121.

^{29}Si NMR (80 MHz, THF-d_8, 25 °C): δ –11.2 (*Si*(CH_3)$_2$). Signal for Al-*Si*H_2Ph not detected.

^{29}Si NMR (80 MHz, C_6D_6:THF-d_8 (4:1), 25 °C): δ –10.4 (*Si*(CH_3)$_2$). Signal for Al-*Si*H_2Ph not detected.

m.p.: 168 °C – 171 °C (dec.).

Elemental analysis: Calculated values for $C_{42}H_{70}AlCsN_2O_3Si_3$ (895.17 g/mol): C 56.35, H 7.88, N 3.13; Found: C 56.54, H 7.77, N 2.95.

IR (Nujol, cm^{-1}): 1\λ 2058 (w), 1686 (w), 1669 (w), 1429 (w), 1314 (w), 1244 (w), 1189 (w), 1107 (w), 1053 (w), 1042 (w), 1001 (m), 944 (w), 935 (w), 924 (w), 848 (w), 802 (w), 794 (w), 774 (w), 719 (w), 673 (w), 653 (w).

Preparation of [{Rb(THF)$_4$}$_2$(Rb{Al(NON^{Dipp})(H)(SiH$_2$Ph)}$_2$)][Rb{Al(NON^{Dipp})(H)(SiH$_2$Ph)}$_2$] (2)

In a 25 mL Schlenk flask [Rb{Al(NON^{Dipp})}]$_2$ (245 mg, 0.412 mmol) was dissolved in hexane (14 mL) to give a yellow solution. $PhSiH_3$ (56 µL, 0.454 mmol, 1.10 equiv.) was added resulting in the precipitation of a white solid within one hour of stirring at room temperature. After stirring the reaction mixture for

additional 16 hours at room temperature the precipitate was collected via filtration and subsequently washed with *n*-hexane (4 x 2 mL). The crude product was then dissolved in THF (2 mL) and layered with n-hexane. Storing this solution at -20 °C for one day yielded colourless crystals suitable for single X-ray diffraction. Isolation of the crystalline material and drying at high vacuum the target compound was obtained as white solid in 69 % (239 mg, 0.141 mmol) yield.

^{1}H NMR (400 MHz, THF-d_8, 25 °C): δ 6.95 – 6.91 (m, 4H, Ar-C*H*), 6.82 – 6.79 (m, 3H, Ar-C*H*), 6.77 – 6.67 (m, 4H, Ar-C*H*), 4.15 (hept, *J* = 6.9 Hz, 2H, C*H*(CH_3)$_2$), 4.04 (hept, *J* = 6.9 Hz, 2H, C*H*(CH_3)$_2$), 3.65 – 3.59 (m, THF), 3.30 (d, *J* = 3.1 Hz, 2H, SiH_2Ph), 1.80 – 1.76 (m, THF), 1.22 – 1.11 (m, 24H, CH(CH_3)$_2$), 0.14 (s, 6H, Si(CH_3)$_2$), 0.06 (s, 6H, Si(CH_3)$_2$).

^{1}H NMR (400 MHz, C_6D_6:THF-d_8 (10:1), 25 °C): δ 7.11 – 7.02 (m, 6H, Ar-C*H*), 6.95 – 6.88 (m, 5H, Ar-C*H*), 4.39 (hept, *J* = 6.7 Hz, 2H, C*H*(CH_3)$_2$), 4.28 (hept, *J* = 6.8 Hz, 2H, C*H*(CH_3)$_2$), 3.74 (d, *J* = 3.2 Hz, 2H, SiH_2Ph), 3.55 – 3.52 (m, THF), 1.47 – 1.45 (m, THF), 1.45 – 1.31 (m, 24H, CH(CH_3)$_2$), 0.57 (s, 6H, Si(CH_3)$_2$), 0.43 (s, 6H, Si(CH_3)$_2$)

^{13}C{^{1}H} NMR (101 MHz, THF-d_8, 25 °C): δ 148.5 (C_{arom}), 148.3 (C_{arom}), 148.0 (C_{arom}), 141.0 (C_{arom}), 138.2 (C_{arom}H), 127.1 (C_{arom}H), 126.3 (C_{arom}H), 124.3 (C_{arom}H), 123.8 (C_{arom}H), 122.1 (C_{arom}), 68.4 ($C_{THF}H_2$), 28.0 (C_{aliph}H), 27.9 (C_{aliph}H), 26.7 ($C_{aliph}H_3$), 26.5 ($C_{aliph}H_3$), 26.5 ($C_{THF}H_2$), 26.3 ($C_{aliph}H_3$), 26.0 ($C_{aliph}H_3$), 3.3 (Si(*C*H_3)$_2$), 2.8 (Si(*C*H_3)$_2$)

^{13}C{^{1}H} NMR (101 MHz, C_6D_6:THF-d_8 (10:1), 25 °C): δ 148.3 (C_{arom}), 148.3 (C_{arom}), 148.0 (C_{arom}), 140.1 (C_{arom}), 137.6 (C_{arom}H), 127.2 (C_{arom}H), 126.5 (C_{arom}H), 124.0 (C_{arom}H), 123.9 (C_{arom}H), 122.2 (C_{arom}H), 67.8 ($C_{THF}H_2$), 27.7 (C_{aliph}H), 27.6 (C_{aliph}H), 26.4 ($C_{aliph}H_3$), 26.1 ($C_{aliph}H_3$), 25.8 ($C_{aliph}H_3$), 25.8 ($C_{THF}H_2$), 25.6 ($C_{aliph}H_3$), 3.1 (Si(*C*H_3)$_2$), 2.7 (Si(*C*H_3)$_2$)

^{27}Al NMR (104 MHz, THF-d_8, 25 °C): δ 122.

^{27}Al NMR (104 MHz, C_6D_6:THF-d_8 (10:1), 25 °C): δ 121.

^{29}Si NMR (80 MHz, THF-d_8, 25 °C): δ –11.7 (*Si*(CH_3)$_2$). Signal for Al-*Si*H_2Ph not detected.

^{29}Si NMR (80 MHz, C_6D_6:THF-d_8 (10:1), 25 °C): δ –10.1 (*Si*(CH_3)$_2$). Signal for Al-*Si*H_2Ph not detected.

m.p.: 161 °C – 162 °C (dec.).

Elemental analysis: Calculated values for $C_{84}H_{140}Al_2N_4O_6Rb_2Si_6$ (1695.47 g/mol): C 59.51, H 8.32, N 3.30; Found: C 59.07, H 8.29, N 3.18.

IR (Nujol, cm^{-1}): 1\λ 2052 (w), 1682 (w), 1430 (w), 1312 (w), 1242 (w), 1189 (w), 1108 (w), 1054 (w), 1001 (m), 949 (w), 937 (w), 924 (w), 848 (w), 802 (w), 794 (w), 775 (w), 719 (w), 675 (w), 655 (w).

2) Crystal structure data

[Li(Et_2O)][Al(NON^{Dipp})(H)(SiH_2Ph)]: 1-Li·(Et_2O)

A colorless crystal of [Li(Et_2O)][Al(NON^{Dipp})(H)(SiH_2Ph)] was covered in inert oil and mounted. The crystal was then flash cooled to 120 K in a nitrogen gas stream and kept at this temperature during the experiment. Data were collected using focused micro-source Cu Kα radiation at 1.54184 Å. The measured data was processed with the CrysAlisPro software package. The structure was solved using direct methods with SHELXS and refined to convergence against F^2 for all independent reflections. All non-hydrogen atoms were refined using anisotropic thermal parameters.

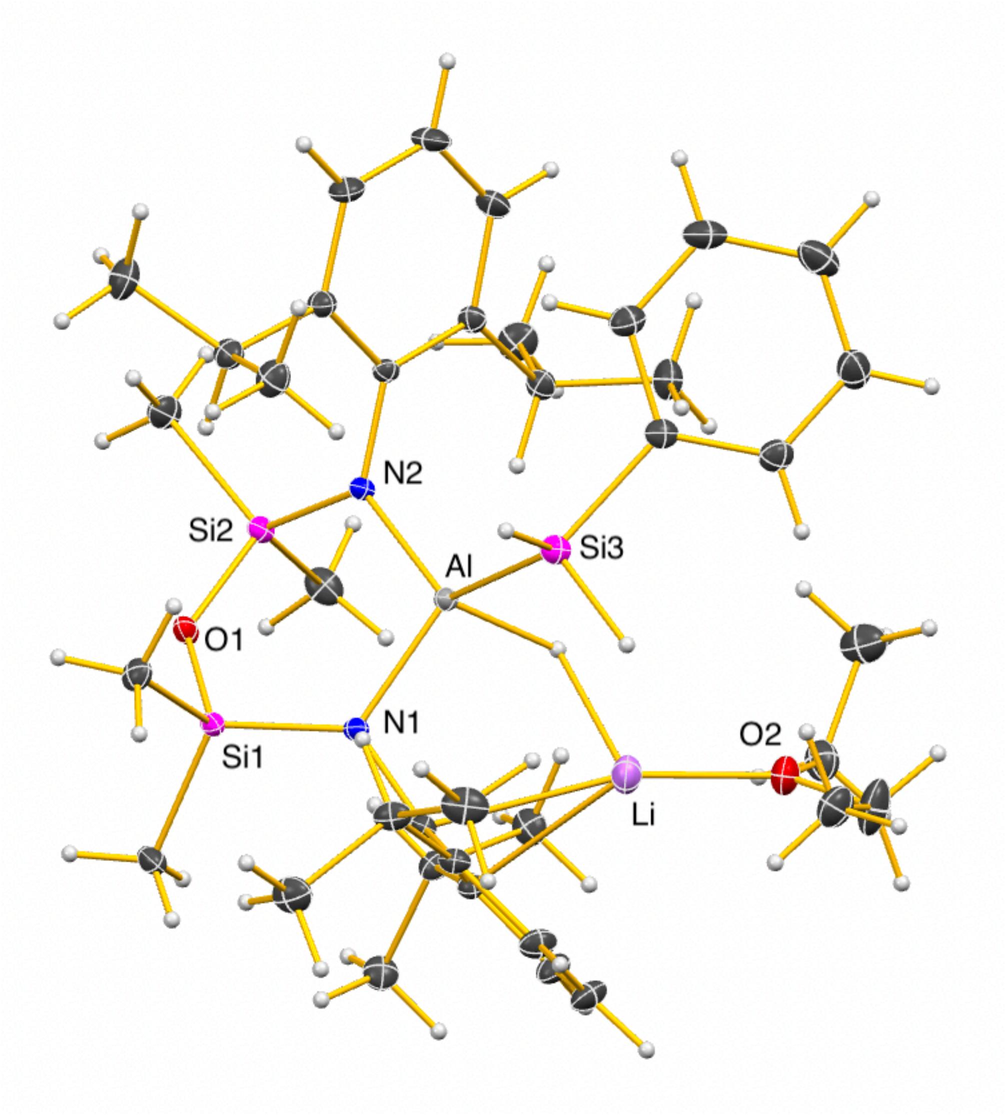

Figure S1. ORTEP plot of the asymmetric unit of **1-Li·(Et_2O)** (ellipsoids drawn at 30% probability).

Table S1. Crystal data and structure refinement $[Li(Et_2O)][Al(NON^{Dipp})(H)(SiH_2Ph)]$	
Identification code	**1-Li·(Et₂O)**
Empirical formula	$C_{38}H_{64}AlLiN_2O_2Si_2$
Formula weight	699.1
Temperature/K	120.0(1)
Crystal system	Monoclinic
Space group	$P2_1/n$
a/Å	12.31742(10)
b/Å	16.73140(11)
c/Å	20.45195(16)
α/°	90
β/°	91.8578(7)
γ/°	90
Volume/Å^3	4212.68(5)
Z	4
ρ_{calc}g/cm^3	1.102
μ/mm^{-1}	1.476
F(000)	1520
Crystal size/mm^3	0.359 × 0.230 × 0.107
Crystal colour	Colorless
Radiation	Cu Kα (λ = 1.54184)
2Θ range for data collection/°	6.827 to 146.527
Index ranges	-14 ≤ h ≤ 15, -20 ≤ k ≤ 20, -25 ≤ l ≤ 25
Reflections collected	30688
Independent reflections	8405 [R_{int} = 0.0227, R_{sigma} = 0.0345]
Data/restraints/parameters	8405/0/450
Goodness-of-fit on F^2	1.04
Final R indexes [I>=2σ (I)]	R_1 = 0.0314, wR_2 = 0.0826
Final R indexes [all data]	R_1 = 0.0341, wR_2 = 0.0847
Largest diff. peak/hole / e Å^{-3}	0.29/-0.35

[Na(Et$_2$O)][Al(NON^{Dipp})(H)(SiH$_2$Ph)]: 1-Na·(Et$_2$O)

A colorless crystal of [Na(Et$_2$O)][Al(NON^{Dipp})(H)(SiH$_2$Ph)] was covered in inert oil and mounted. The crystal was then flash cooled to 120 K in a nitrogen gas stream and kept at this temperature during the experiment. Data were collected using focused micro-source Cu Kα radiation at 1.54184 Å. The measured data was processed with the CrysAlisPro software package. The structure was solved using direct methods with SHELXS and refined to convergence against F^2 for all independent reflections. All non-hydrogen atoms were refined using anisotropic thermal parameters.

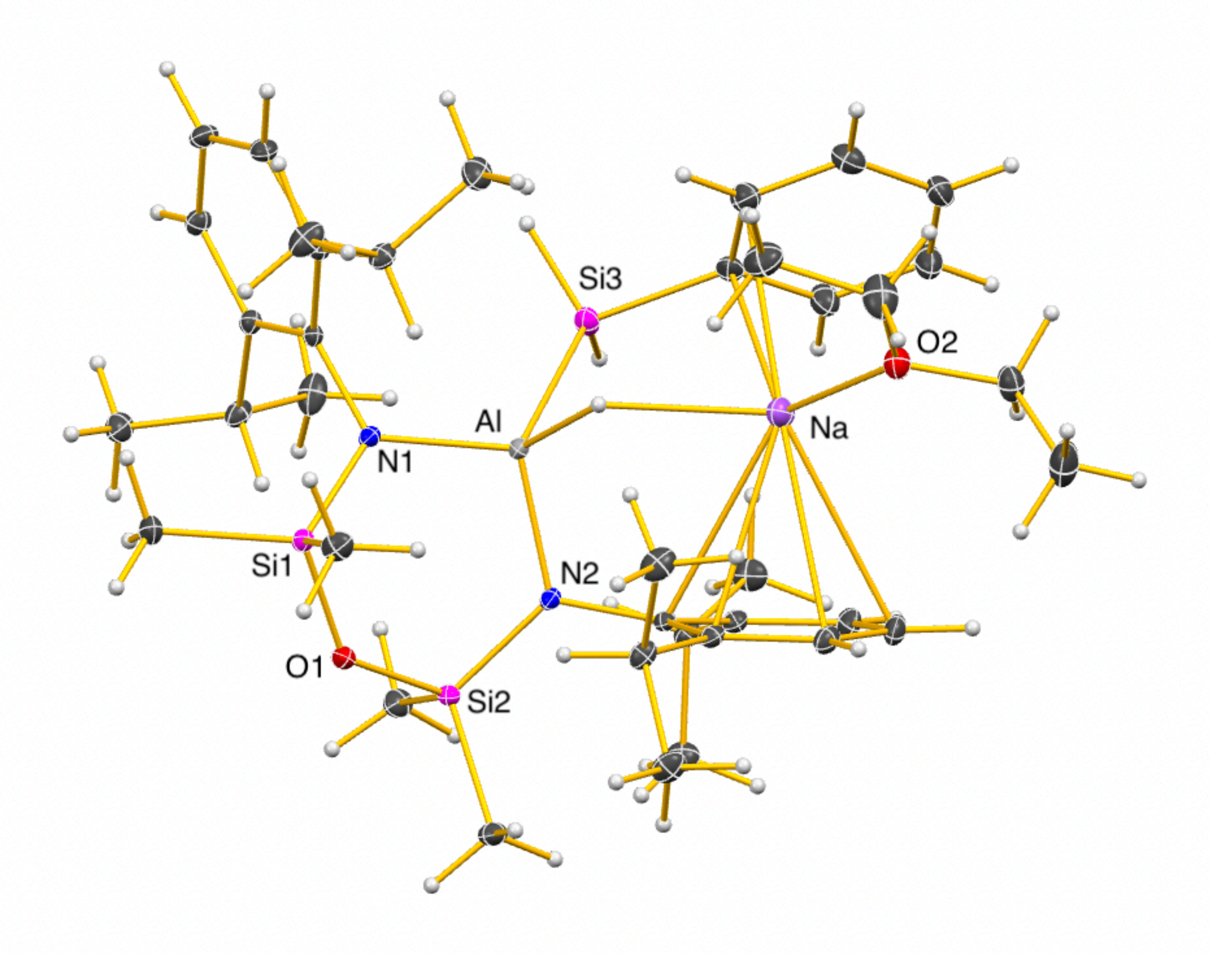

Figure S2. ORTEP plot of the asymmetric unit of **1-Na·(Et$_2$O)** (ellipsoids drawn at 30% probability).

**Table S2.** Crystal data and structure refinement $[Na(Et_2O)][Al(NON^{Dipp})(H)(SiH_2Ph)]$	
Identification code	**1-Na·(Et_2O)**
Empirical formula	$C_{38}H_{64}AlN_2NaO_2Si_2$
Formula weight	715.15
Temperature/K	120.0(1)
Crystal system	Monoclinic
Space group	$P2_1/c$
a/Å	18.1846(2)
b/Å	9.9726(1)
c/Å	24.7217(3)
α/°	90
β/°	112.955(1)
γ/°	90
Volume/Å^3	4128.20(8)
Z	4
ρ_{calc}g/cm^3	1.151
μ/mm^{-1}	1.616
F(000)	1552
Crystal size/mm^3	0.301 × 0.139 × 0.097
Crystal colour	Colorless
Radiation	Cu Kα (λ = 1.54184)
2Θ range for data collection/°	7.498 to 146.494
Index ranges	-21 ≤ h ≤ 22, -12 ≤ k ≤ 12, -29 ≤ l ≤ 30
Reflections collected	29782
Independent reflections	8236 [R_{int} = 0.0243, R_{sigma} = 0.0241]
Data/restraints/parameters	8236/0/450
Goodness-of-fit on F^2	1.026
Final R indexes [I>=2σ (I)]	R_1 = 0.0293, wR_2 = 0.0738
Final R indexes [all data]	R_1 = 0.0320, wR_2 = 0.0759
Largest diff. peak/hole / e Å^{-3}	0.34/-0.24

[Rb(Et_2O)][Al(NON^{Dipp})(H)(SiH_2Ph)]: 1-Rb·(Et_2O)

A colorless crystal of [Rb(Et_2O)][Al(NON^{Dipp})(H)(SiH_2Ph)] was embedded in inert perfluoropolyalkylether (viscosity 1800 cSt; ABCR GmbH) and mounted using a glass fiber. The crystal was then flash cooled to 100 K in a nitrogen gas stream and kept at this temperature during the experiment. The crystal structure was measured with a Rigaku XtaLAB Synergy-i with monochromated Cu−Kα (λ = 1.54184 Å) radiation. The measured data was processed with the CrysAlisPro software package. Using Olex2, the structure was solved with the ShelXT structure solution program and refined to convergence against F^2 for all independent reflections. All non-hydrogen atoms were refined using anisotropic thermal parameters.

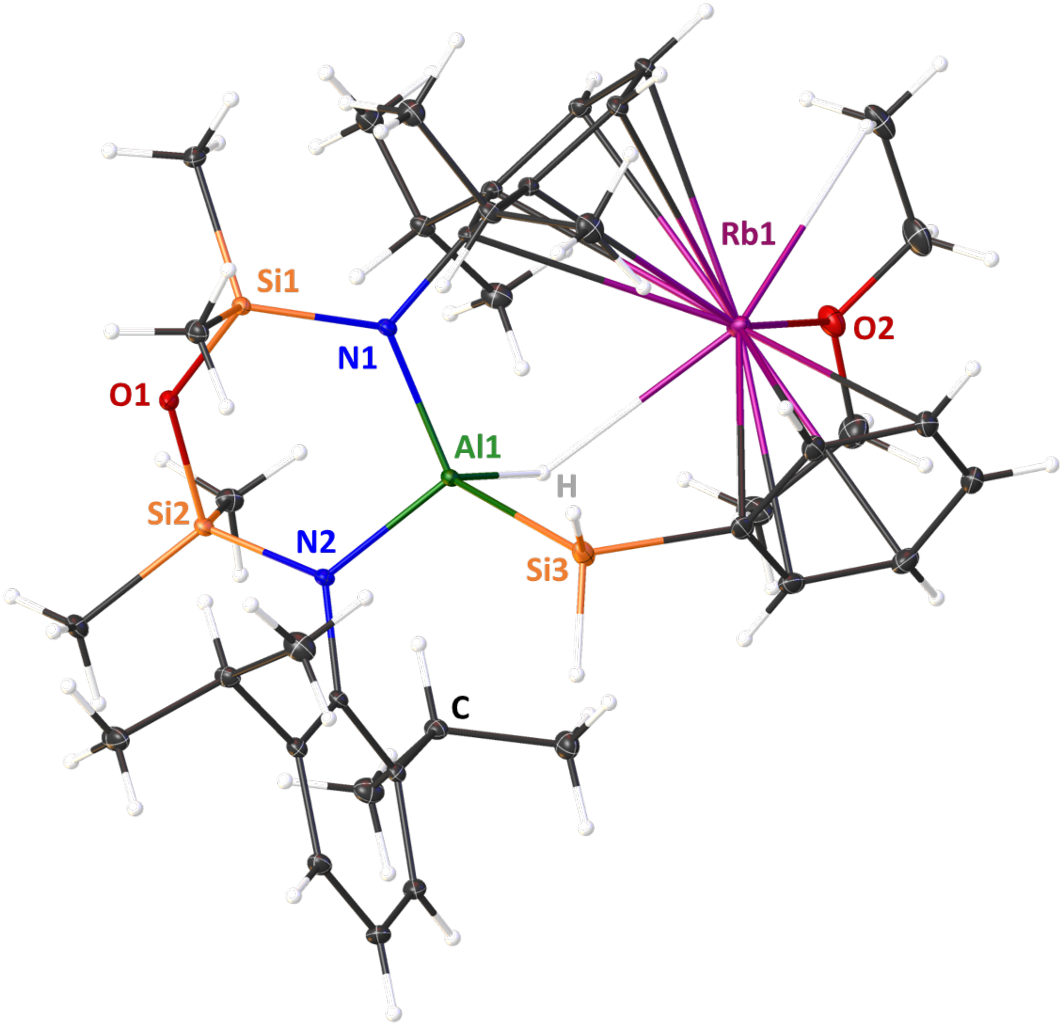

Figure S3. ORTEP plot of the asymmetric unit of **1-Rb·(Et_2O)** (ellipsoids drawn at 30% probability).

**Table S3.** Crystal data and structure refinement for [Rb{NON^{Dipp})Al(H)SiH$_2$Ph}(Et$_2$O)] **1-Rb·(Et$_2$O)**	
Identification code	[Rb{(NONDipp)Al(H)SiH2Ph}(Et2O)]
Empirical formula	$AlRbSi_3O_2N_2C_{38}H_{64}$
Formula weight	777.63
Temperature/K	99.9(2)
Crystal system	Monoclinic
Space group	$P2_1/c$
a/Å	18.52230(10)
b/Å	10.13970(10)
c/Å	24.16980(10)
α/°	90
β/°	111.4380(10)
γ/°	90
Volume/Å^3	4225.28(6)
Z	4
ρ_{calc}g/cm^3	1.222
μ/mm^{-1}	2.872
F(000)	1656.0
Crystal size/mm^3	0.304 × 0.25 × 0.148
Crystal colour	colourless
Radiation	Cu Kα (λ = 1.54184)
2Θ range for data collection/°	5.126 to 146.008
Index ranges	-22 ≤ h ≤ 21, -12 ≤ k ≤ 12, -29 ≤ l ≤ 29
Reflections collected	85365
Independent reflections	8389 [R_{int} = 0.0219, R_{sigma} = 0.0091]
Data/restraints/parameters	8389/0/482
Goodness-of-fit on F^2	1.065
Final R indexes [I>=2σ (I)]	R_1 = 0.0251, wR_2 = 0.0624
Final R indexes [all data]	R_1 = 0.0253, wR_2 = 0.0625
Largest diff. peak/hole / e Å^{-3}	0.50/-0.53

[Cs(THF)$_2$][Al(NON^{Dipp})(H)(SiH$_2$Ph)]: 1-Cs·(THF)$_2$

A colorless crystal of [Cs(THF)$_2$][Al(NON^{Dipp})(H)(SiH$_2$Ph)] was embedded in inert perfluoropolyalkylether (viscosity 1800 cSt; ABCR GmbH) and mounted using a glass fiber. The crystal was then flash cooled to 100 K in a nitrogen gas stream and kept at this temperature during the experiment. The crystal structure was measured with an Oxford Diffraction Gemini E instrument with graphite-monochromated Mo−Kα (λ = 0.71073 Å) radiation. The measured data was processed with the CrysAlisPro software package. Using Olex2, the structure was solved with the ShelXT structure solution program and refined to convergence against F^2 for all independent reflections. All non-hydrogen atoms were refined using anisotropic thermal parameters. One of the THF ligands was modelled as disordered over two sites. Restraints and constraints were added to this disordered group to ensure that geometry and displacement ellipsoids approximated normal behavior. The occupancies of the disordered sites refined to 66.4(6):33.6(6).

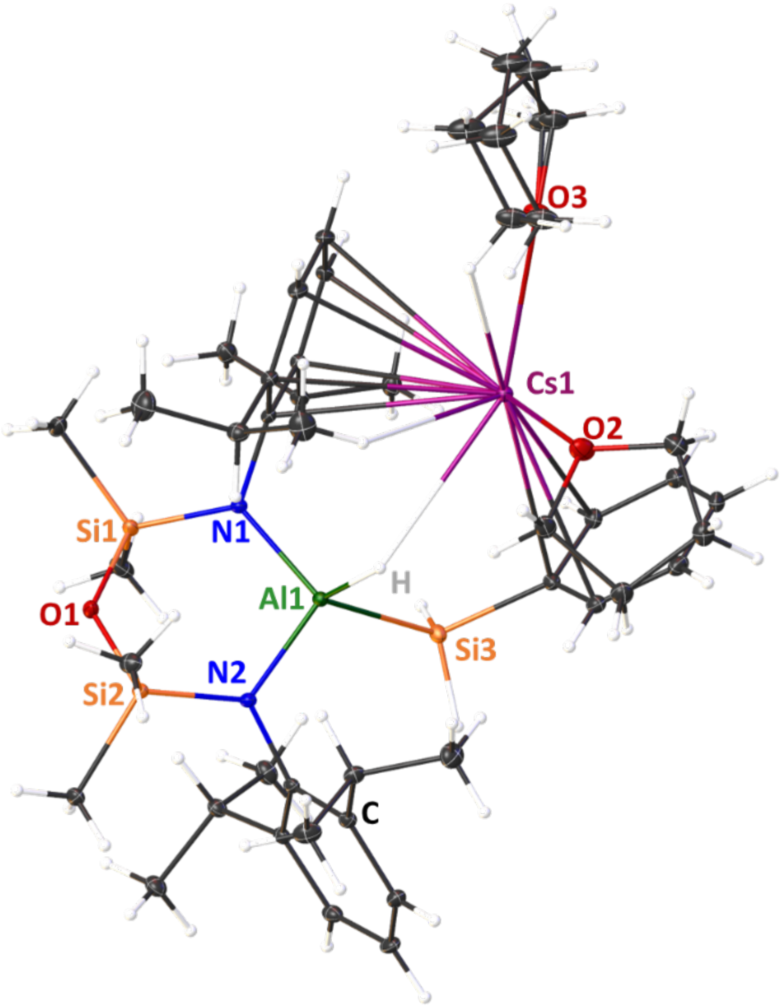

Figure S4. ORTEP plot of **1-Cs·(THF)$_2$** (ellipsoids drawn at 30% probability).

**Table S4.** Crystal data and structure refinement for [Cs{(NON^{Dipp})Al(H)SiH$_2$Ph}(THF)$_2$] (**1-Cs·(THF)$_2$**)	
Identification code	[Cs{(NONDipp)Al(H)SiH2Ph}(THF)2]
Empirical formula	$AlCsSi_3O_3N_2C_{42}H_{70}$
Formula weight	895.16
Temperature/K	100.0(3)
Crystal system	Monoclinic
Space group	I2/a
a/Å	23.19630(10)
b/Å	10.60780(10)
c/Å	38.8489(2)
α/°	90
β/°	100.9930(10)
γ/°	90
Volume/Å^3	9383.82(11)
Z	8
ρ_{calc}g/cm^3	1.267
μ/mm^{-1}	7.350
F(000)	3760.0
Crystal size/mm^3	0.16 × 0.12 × 0.05
Crystal colour	colourless
Radiation	Cu Kα (λ = 1.54184)
2Θ range for data collection/°	4.634 to 145.674
Index ranges	-28 ≤ h ≤ 28, -13 ≤ k ≤ 13, -48 ≤ l ≤ 48
Reflections collected	115639
Independent reflections	9313 [R_{int} = 0.0372, R_{sigma} = 0.0148]
Data/restraints/parameters	9313/50/517
Goodness-of-fit on F^2	1.046
Final R indexes [I>=2σ (I)]	R_1 = 0.0217, wR_2 = 0.0572
Final R indexes [all data]	R_1 = 0.0222, wR_2 = 0.0574
Largest diff. peak/hole / e Å^{-3}	0.52/-0.42

[{Rb(THF)$_4$}$_2$(Rb{Al(NON^{Dipp})(H)(SiH$_2$Ph)}$_2$)][Rb{Al(NON^{Dipp})(H)(SiH$_2$Ph)}$_2$] (2)

A colorless crystal of [{Rb(THF)$_4$}$_2$(Rb{Al(NON^{Dipp})(H)(SiH$_2$Ph)}$_2$)][Rb{Al(NON^{Dipp})(H)(SiH$_2$Ph)}$_2$] was embedded in inert perfluoropolyalkylether (viscosity 1800 cSt; ABCR GmbH) and mounted using a glass fiber. The crystal was then flash cooled to 100 K in a nitrogen gas stream and kept at this temperature during the experiment. The crystal structure was measured with an Oxford Diffraction Gemini S instrument with graphite-monochromated Mo−Kα (λ = 0.71073 Å) radiation. The measured data was processed with the CrysAlisPro software package. Using Olex2, the structure was solved with the ShelXT structure solution program and refined to convergence against F^2 for all independent reflections. All non-hydrogen atoms were refined using anisotropic thermal parameters. One of the THF ligands was modelled as disordered over two sites. Restraints and constraints were added to this disordered group to ensure that geometry and displacement ellipsoids approximated normal behavior. The occupancies of the disordered sites refined to 72.1(6):27.9(6).

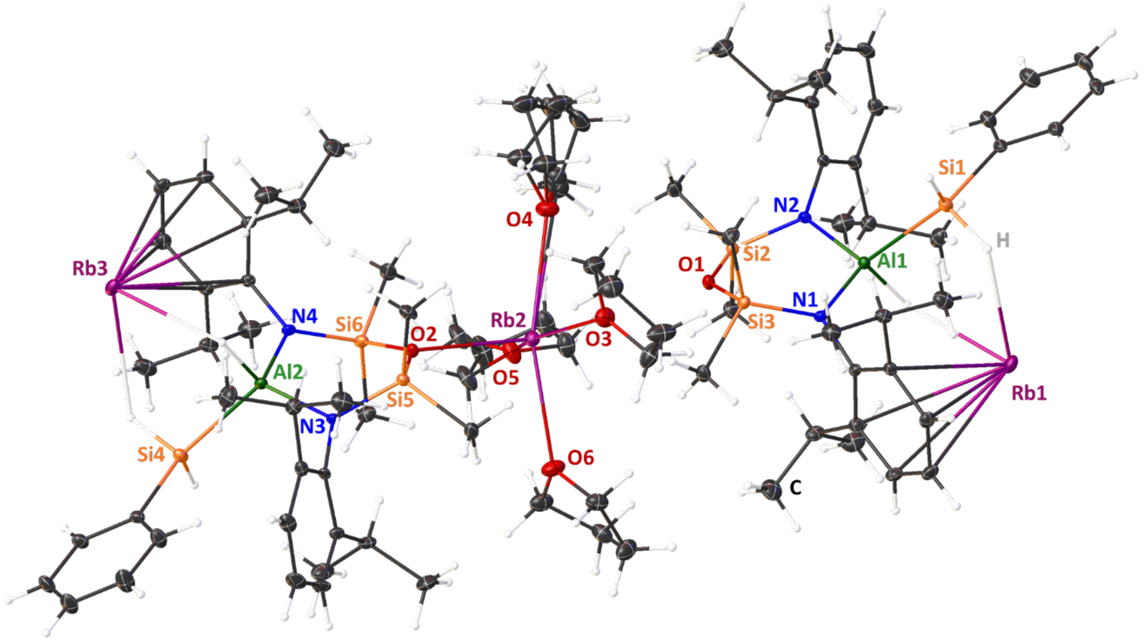

Figure S5. ORTEP plot of the asymmetric unit of (**2**) (ellipsoids drawn at 30% probability).

**Table S5.** Crystal data and structure refinement for $[Rb(THF)_4][Rb\{(NON^{Dipp})Al(H)SiH_2Ph\}_2]$ (**2**)	
Identification code	[Rb(THF)4][Rb{(NONDipp)Al(H)SiH2Ph}2]
Empirical formula	$Al_4Rb_4Si_{12}O_{12}N_8C_{168}H_{280}$
Formula weight	3390.86
Temperature/K	150.05(10)
Crystal system	Triclinic
Space group	P-1
a/Å	12.7686(3)
b/Å	16.1348(4)
c/Å	23.1013(3)
α/°	87.659(2)
β/°	88.426(2)
γ/°	82.309(2)
Volume/Å^3	4711.39(17)
Z	1
ρ_{calc}g/cm^3	1.195
μ/mm^{-1}	1.182
F(000)	1808.0
Crystal size/mm^3	0.29 × 0.24 × 0.2
Crystal colour	colourless
Radiation	Mo Kα (λ = 0.71073)
2Θ range for data collection/°	6.592 to 56
Index ranges	-16 ≤ h ≤ 16, -21 ≤ k ≤ 20, -30 ≤ l ≤ 30
Reflections collected	90525
Independent reflections	21745 [R_{int} = 0.0587, R_{sigma} = 0.0624]
Data/restraints/parameters	21745/14/1005
Goodness-of-fit on F^2	1.026
Final R indexes [I>=2σ (I)]	R_1 = 0.0460, wR_2 = 0.0924
Final R indexes [all data]	R_1 = 0.0830, wR_2 = 0.1077
Largest diff. peak/hole / e Å^{-3}	0.43/-0.45

3) Selected ^{1}H, ^{13}C, DEPT-135, DEPT-90, ^{1}H^{1}H-COSY, ^{1}H^{13}C-HSQC, ^{1}H^{13}C-HMBC, ^{29}Si and ^{27}Al NMR spectra

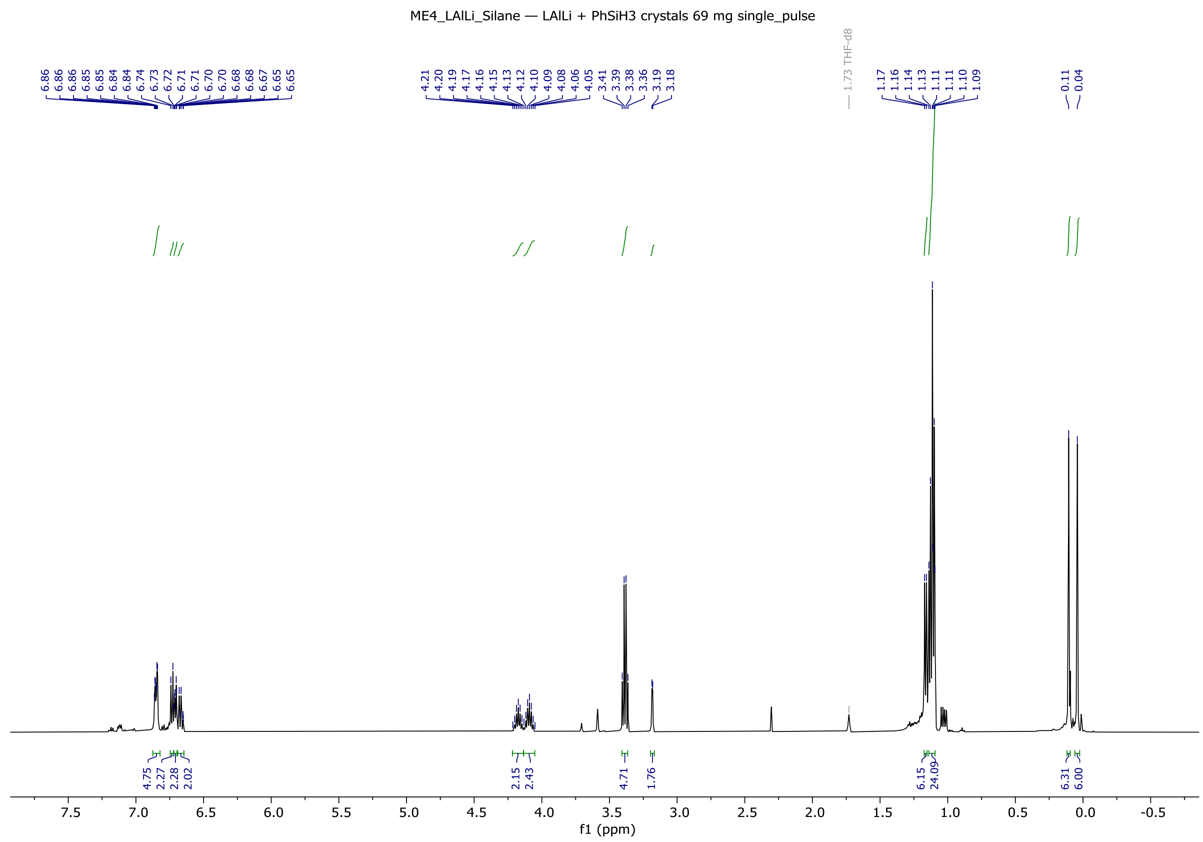

Figure S6. ^{1}H NMR spectrum of **1-Li·(Et$_2$O)** in THF-d_8.

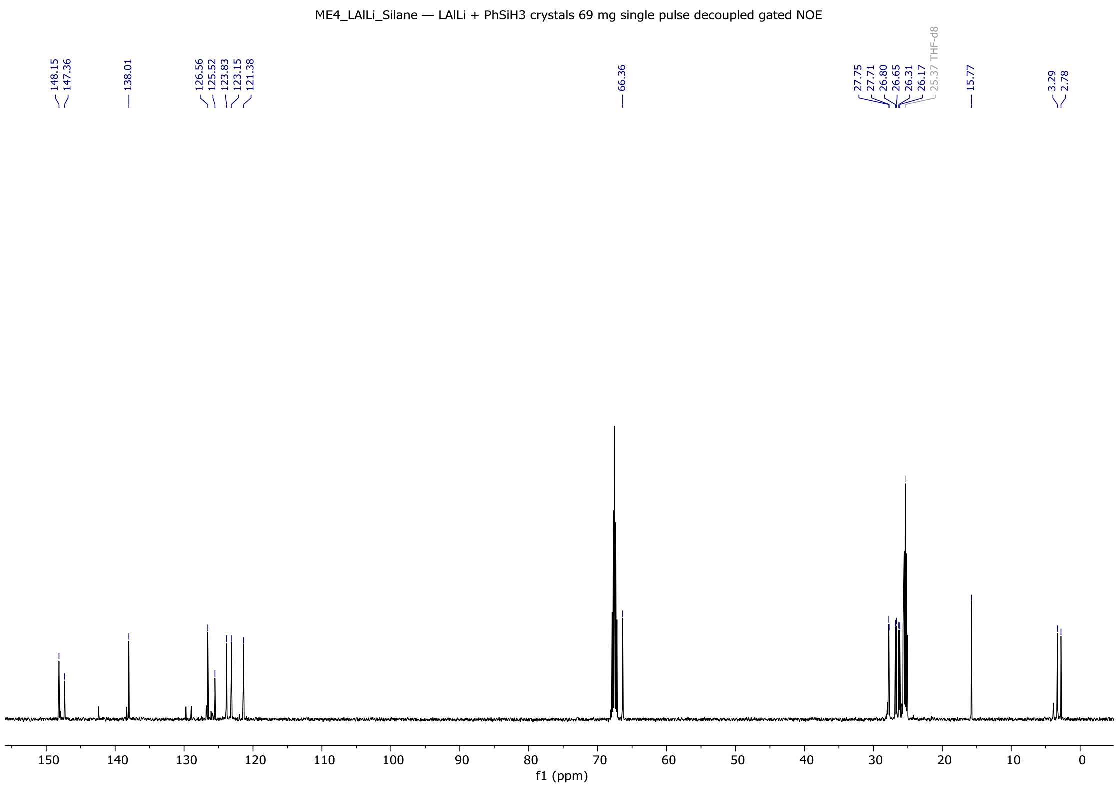

Figure S7. ^{13}C{^{1}H} NMR spectrum of **1-Li·(Et$_2$O)** in THF-d_8.

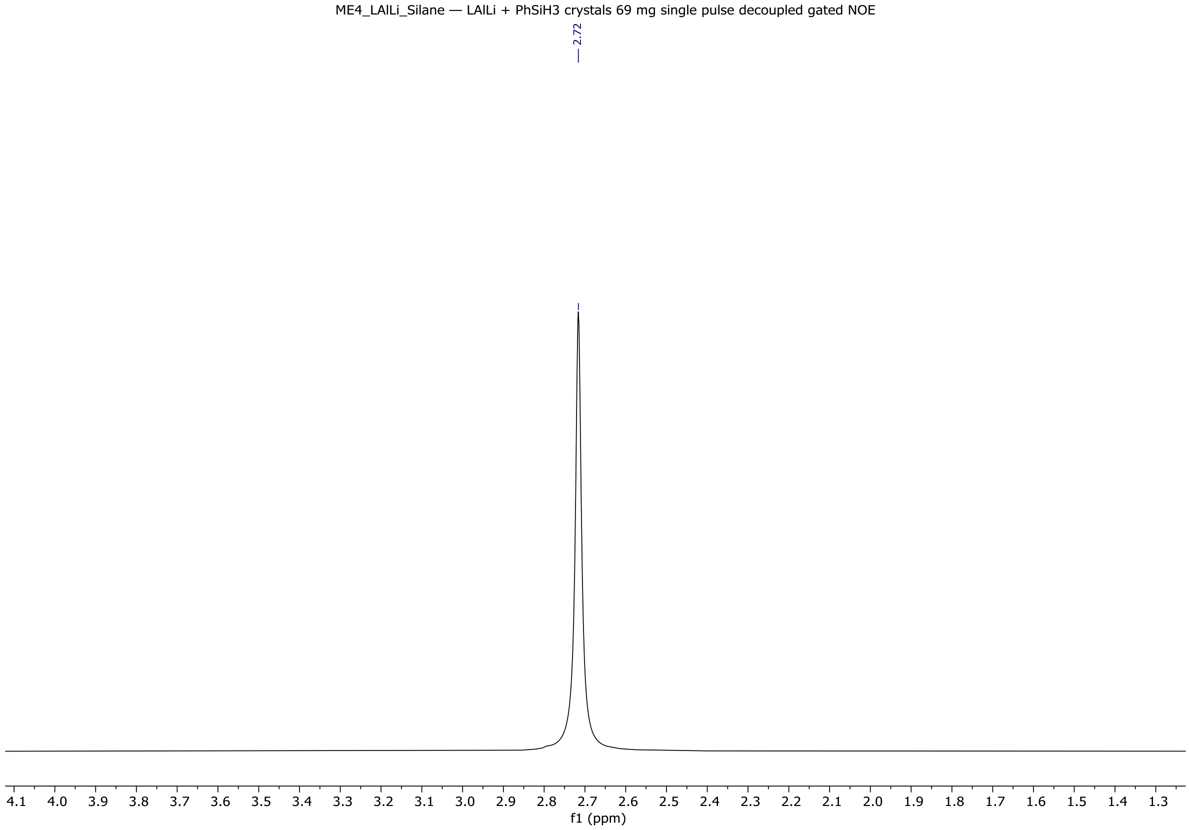

Figure S8. ^{7}Li NMR spectrum of **1-Li·(Et$_2$O)** in THF-d_8.

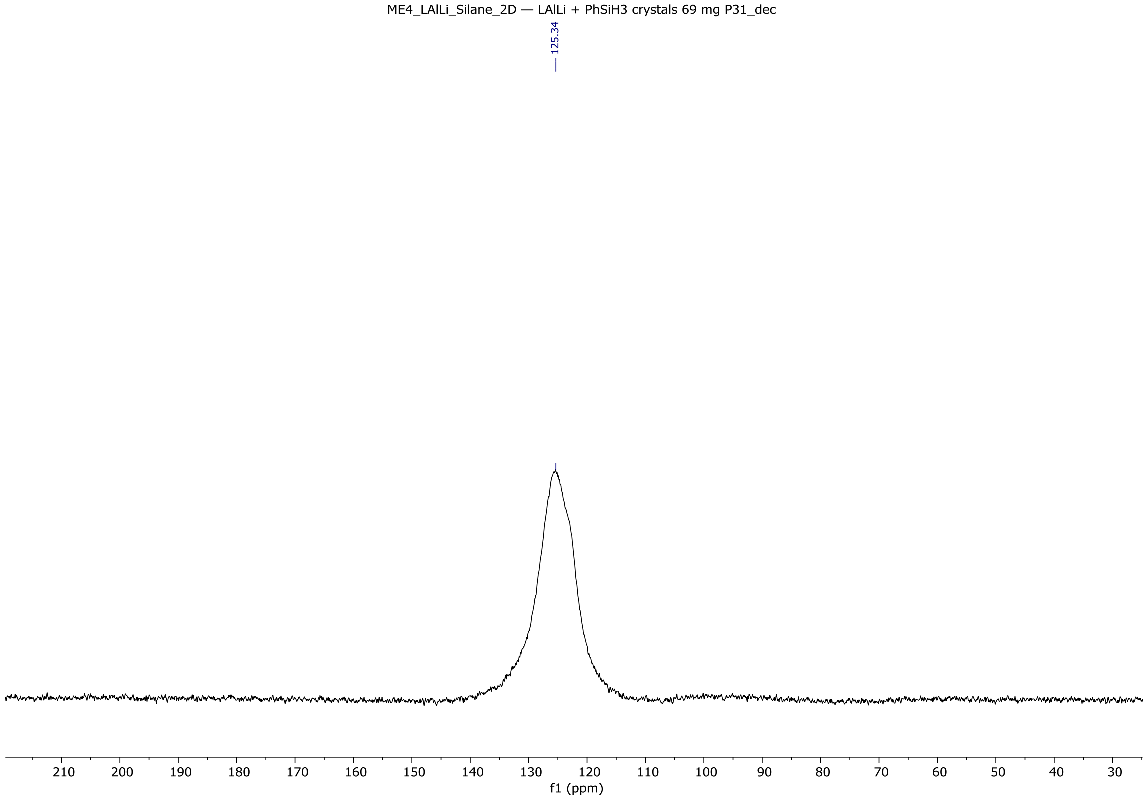

Figure S9. ^{27}Al{^{1}H} NMR spectrum of **1-Li·(Et$_2$O)** in THF-d_8.

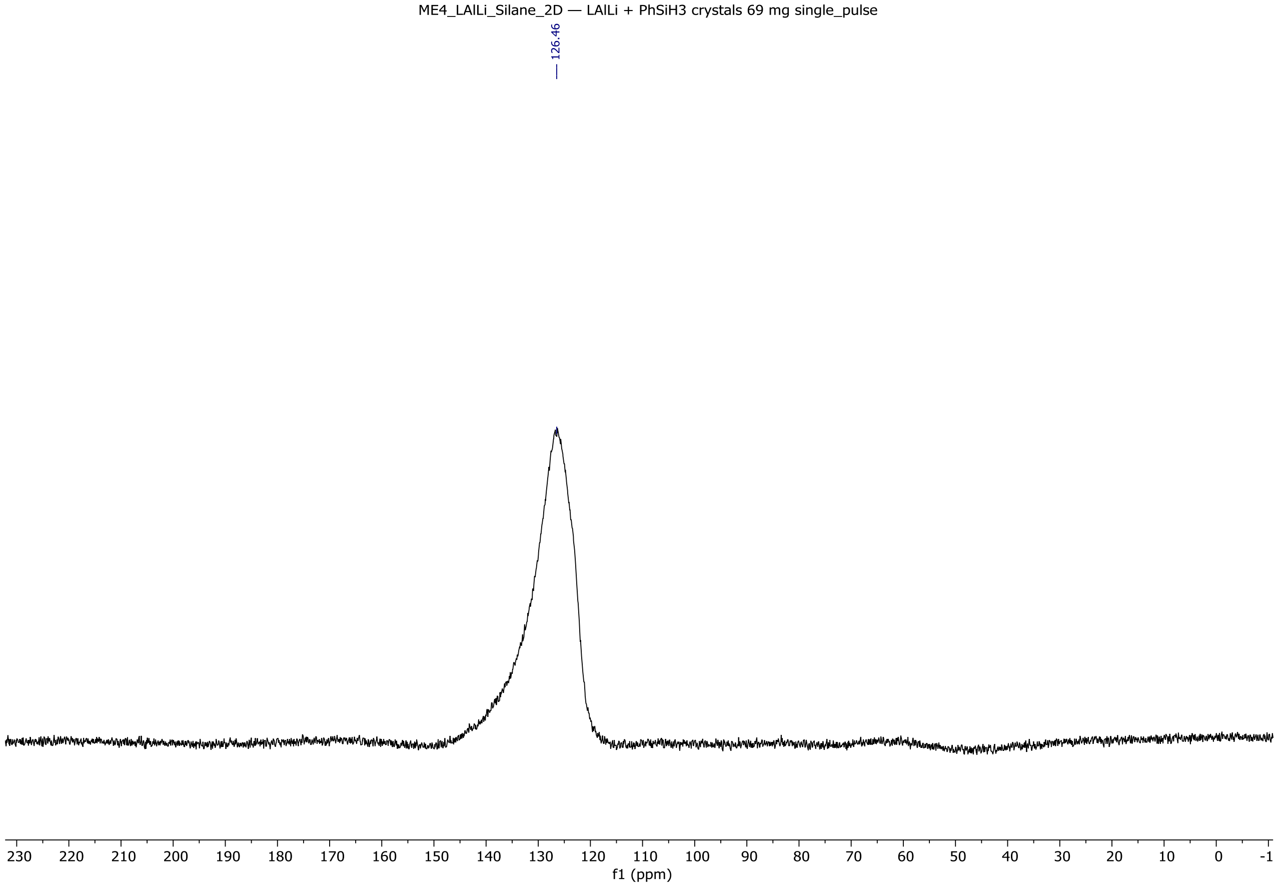

Figure S10. ^{27}Al NMR spectrum of **1-Li·(Et$_2$O)** in THF-d_8.

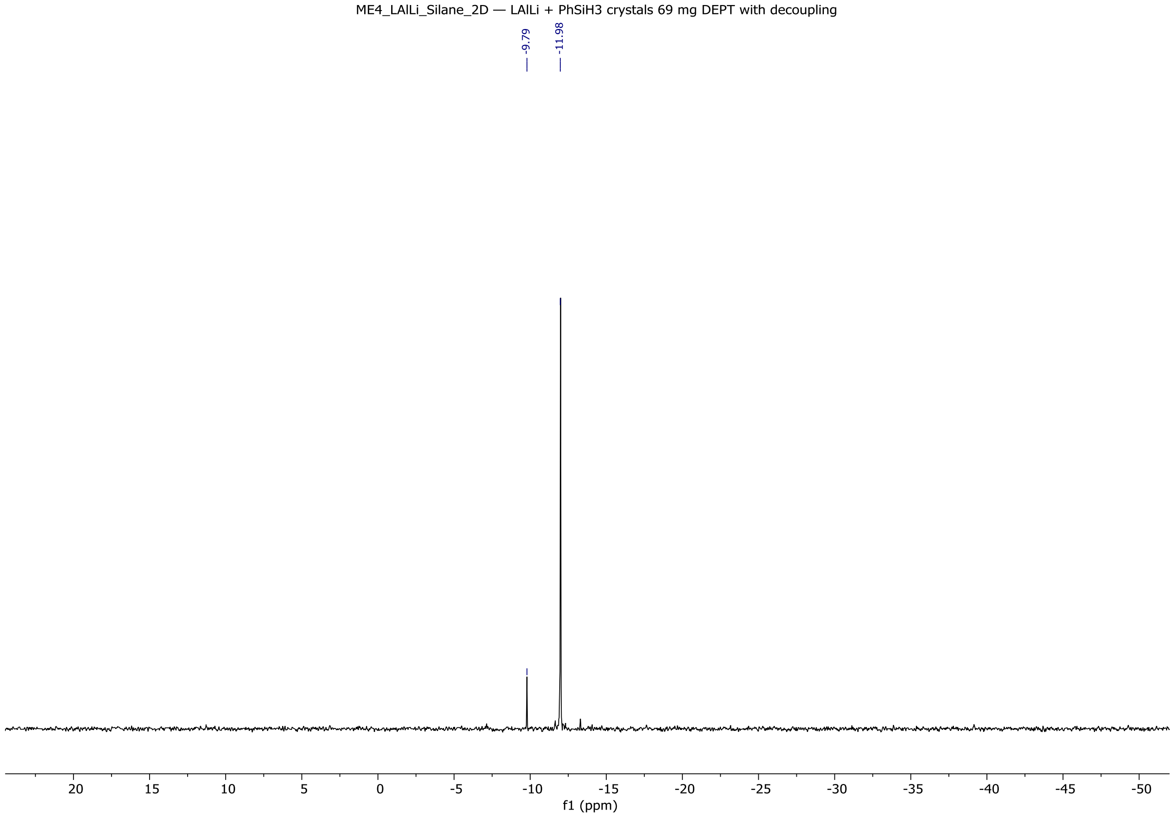

Figure S11. ^{29}Si DEPT NMR spectrum of **1-Li·(Et$_2$O)** in THF-d_8.

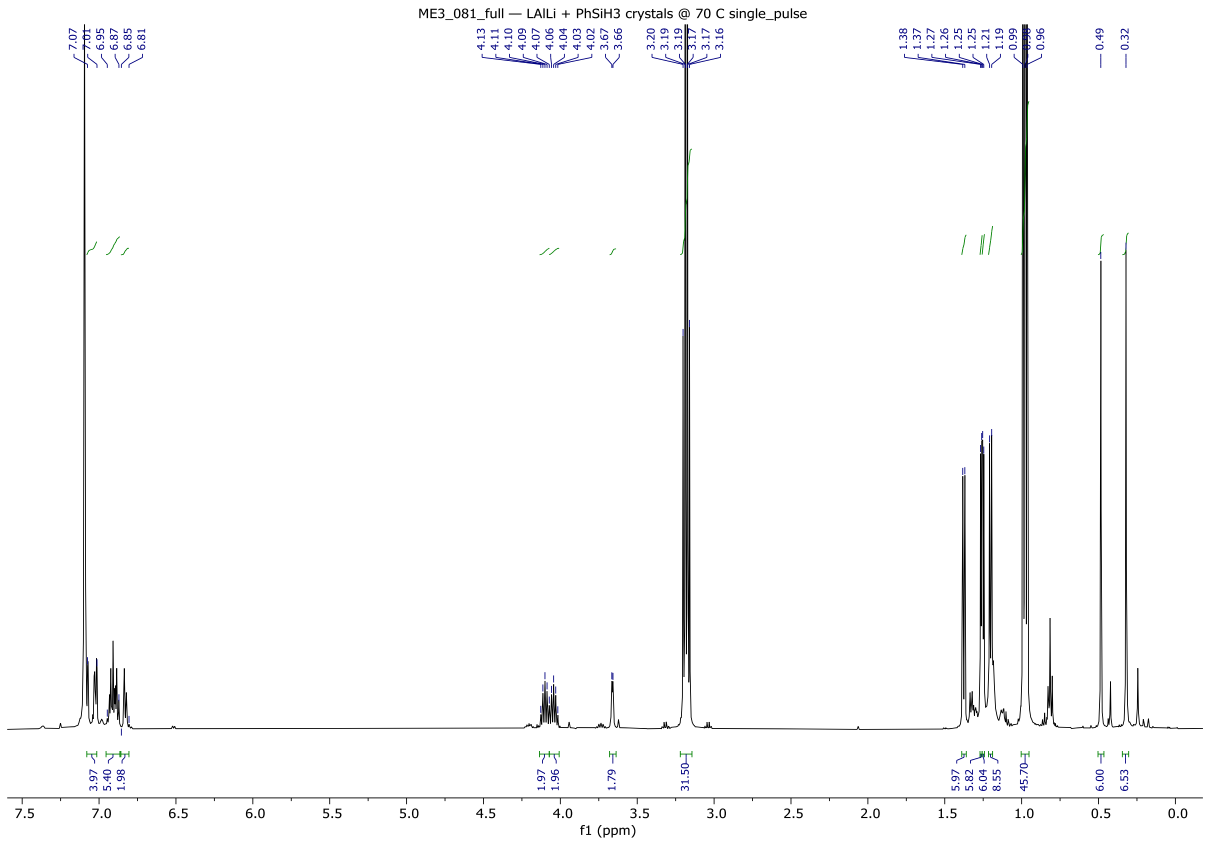

Figure S12. ^{1}H NMR spectrum of **1-Li·(Et$_2$O)** in benzene-*d*$_6$.

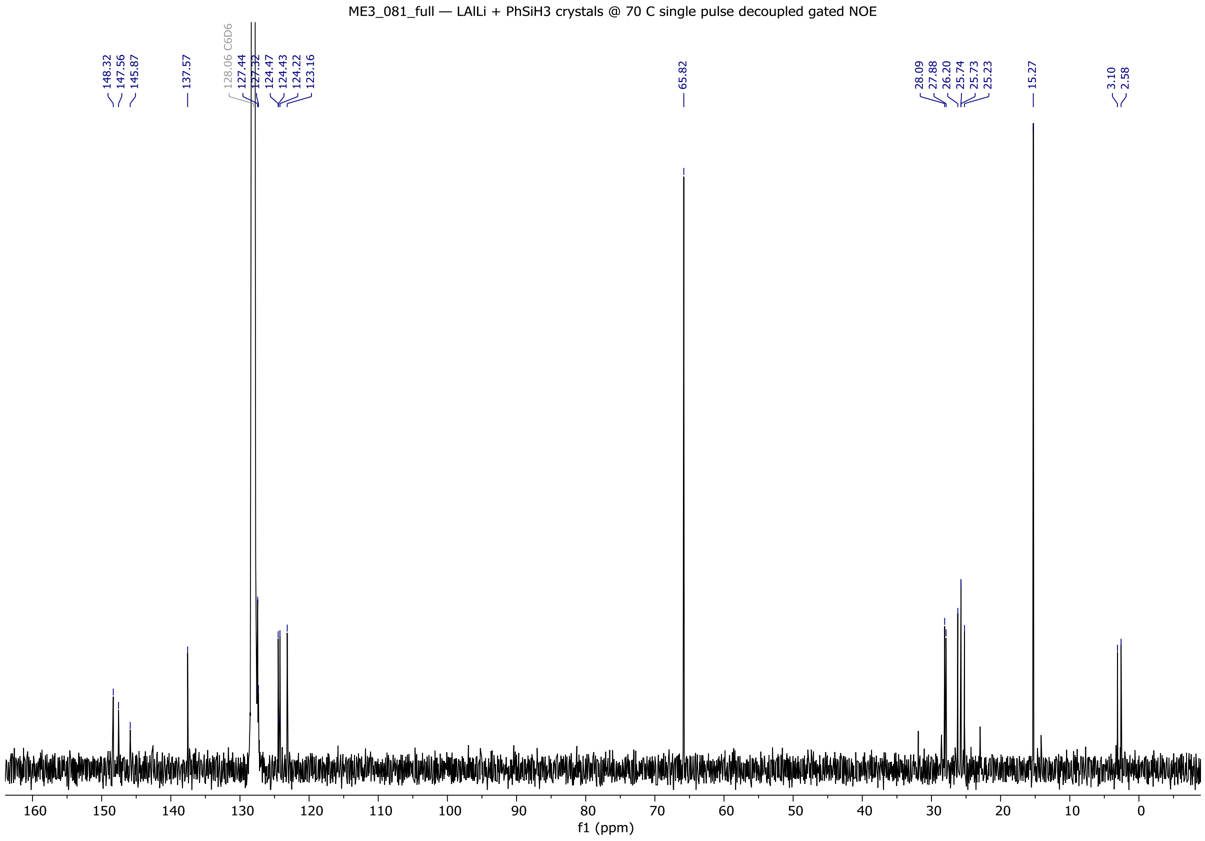

Figure S13. $^{13}C\{^{1}H\}$ NMR spectrum of **1-Li·(Et$_2$O)** in benzene-*d*$_6$.

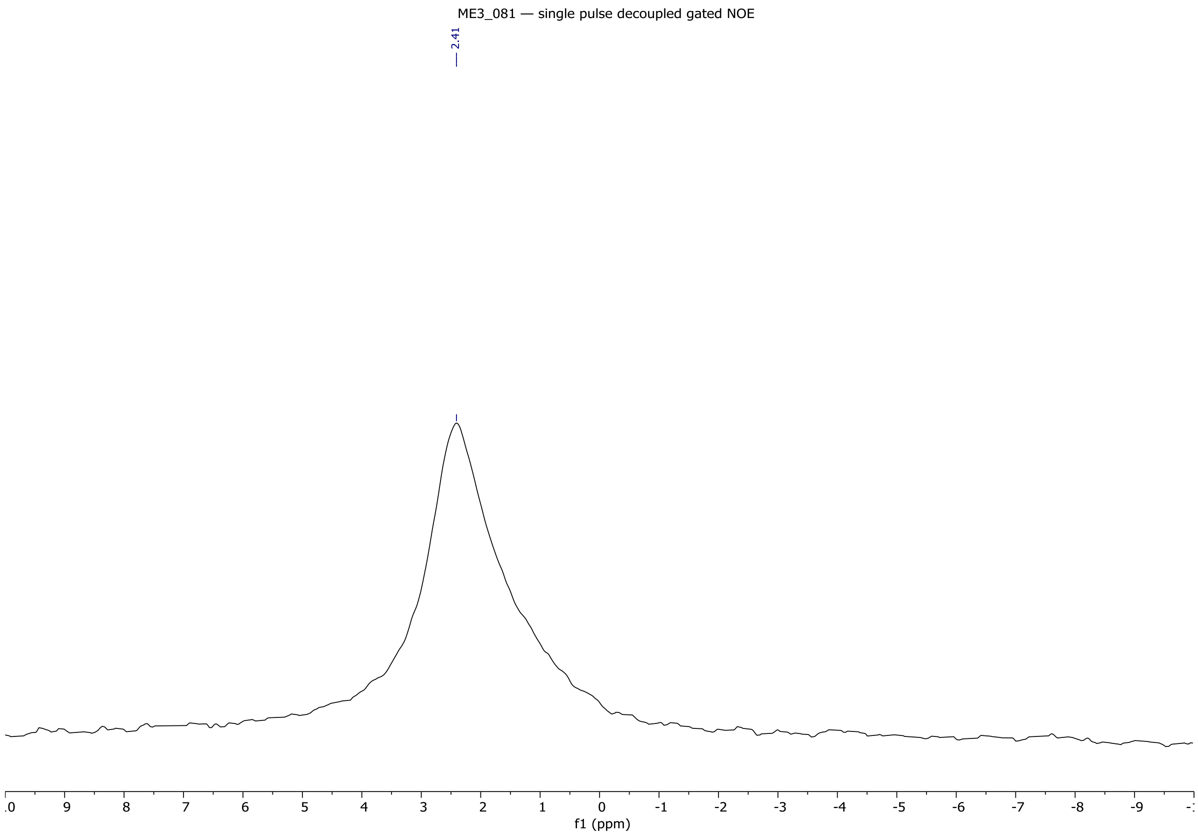

Figure S14. ^{7}Li NMR spectrum of **1-Li·(Et$_2$O)** in benzene-d_6.

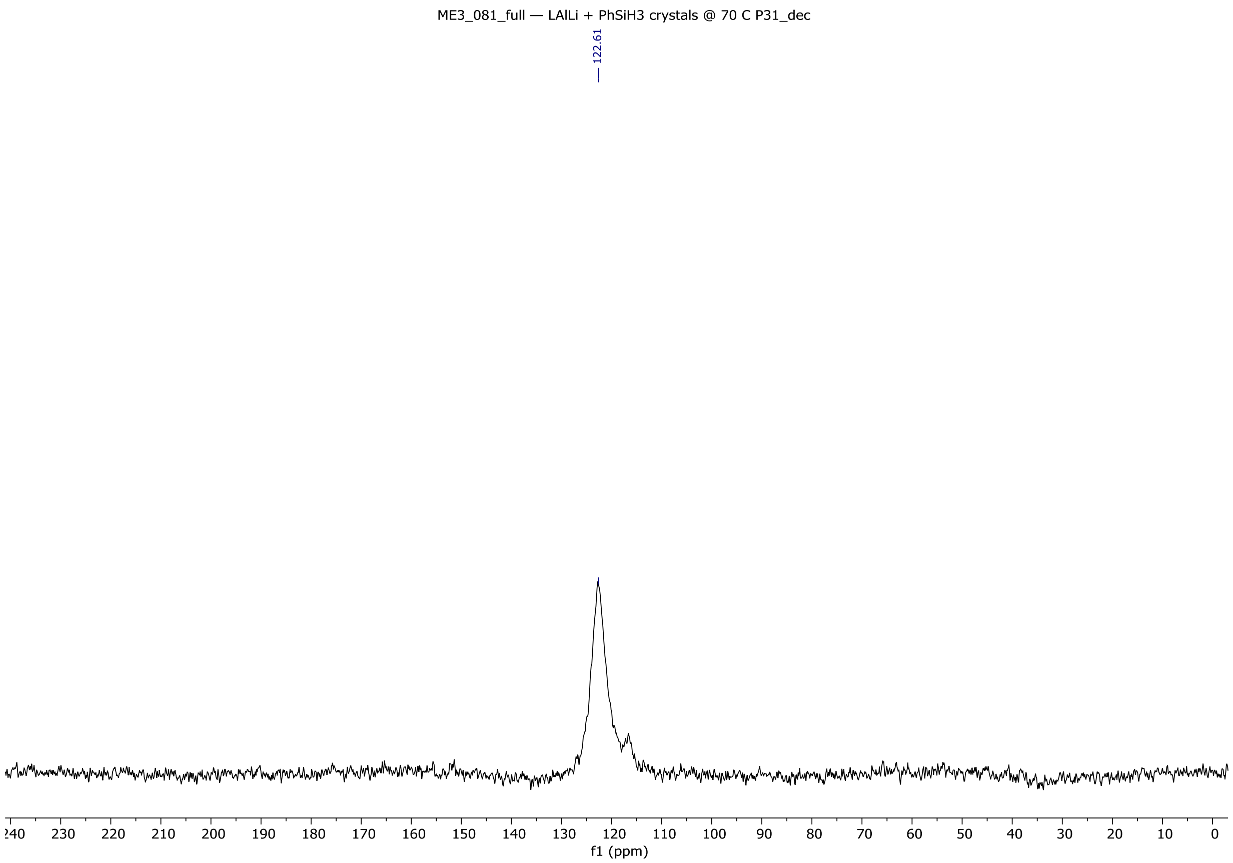

Figure S15. ^{27}Al{^{1}H} NMR spectrum of **1-Li·(Et$_2$O)** in benzene-d_6.

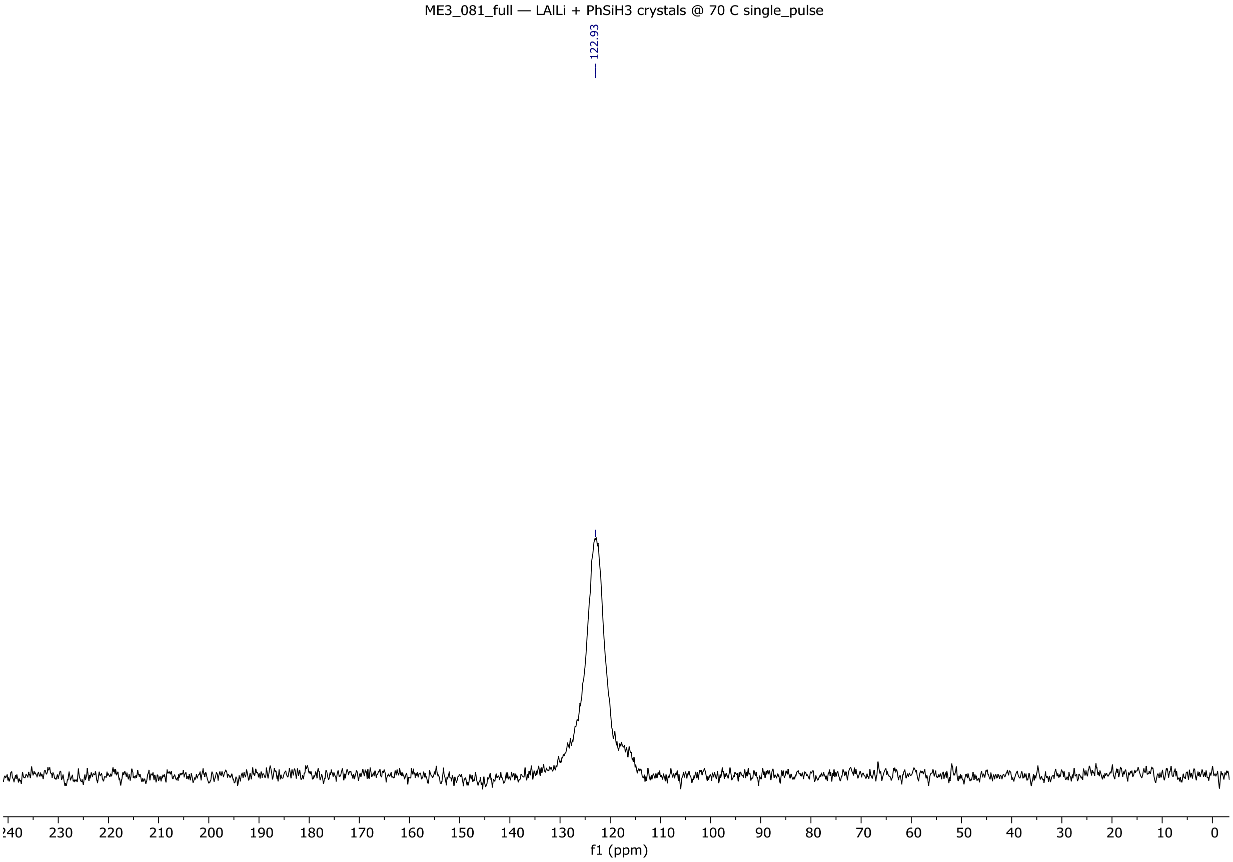

Figure S16. ^{27}Al NMR spectrum of **1-Li·(Et$_2$O)** in benzene-d_6.

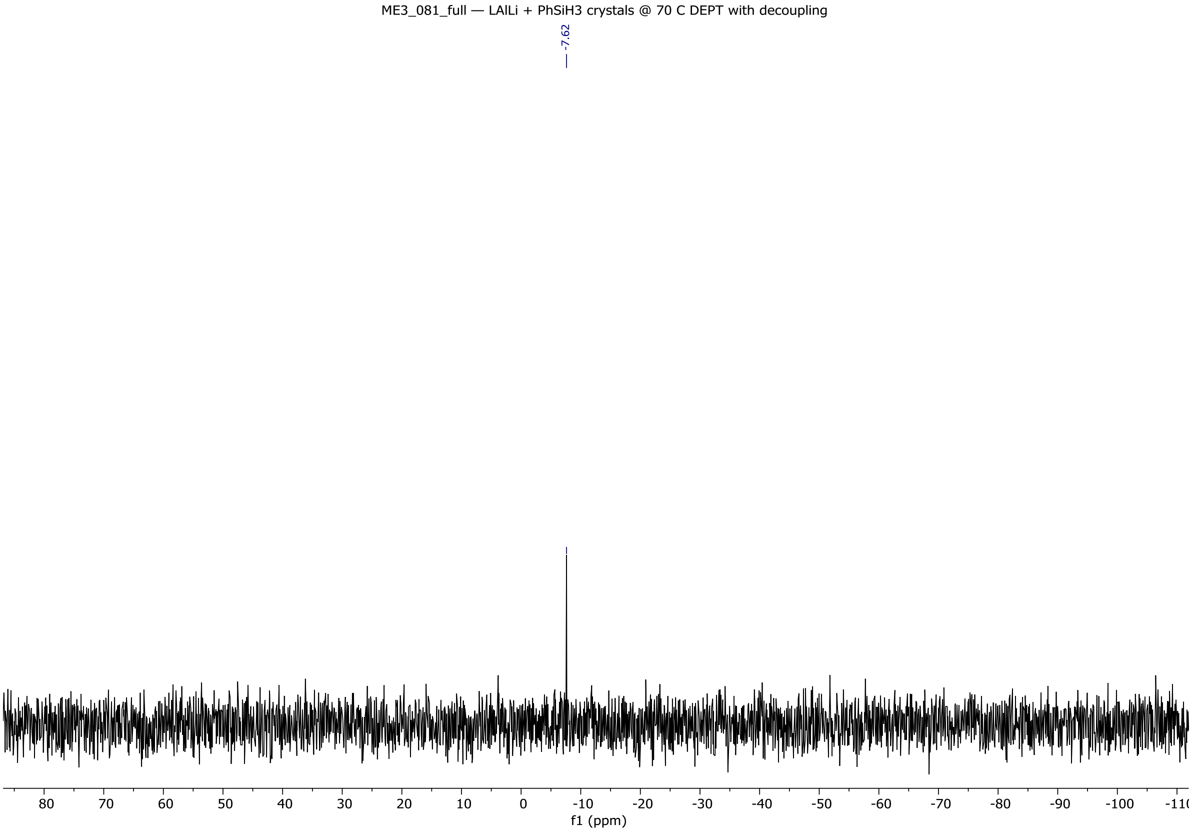

Figure S17. ^{29}Si DEPT NMR spectrum of **1-Li·(Et$_2$O)** in benzene-d_6.

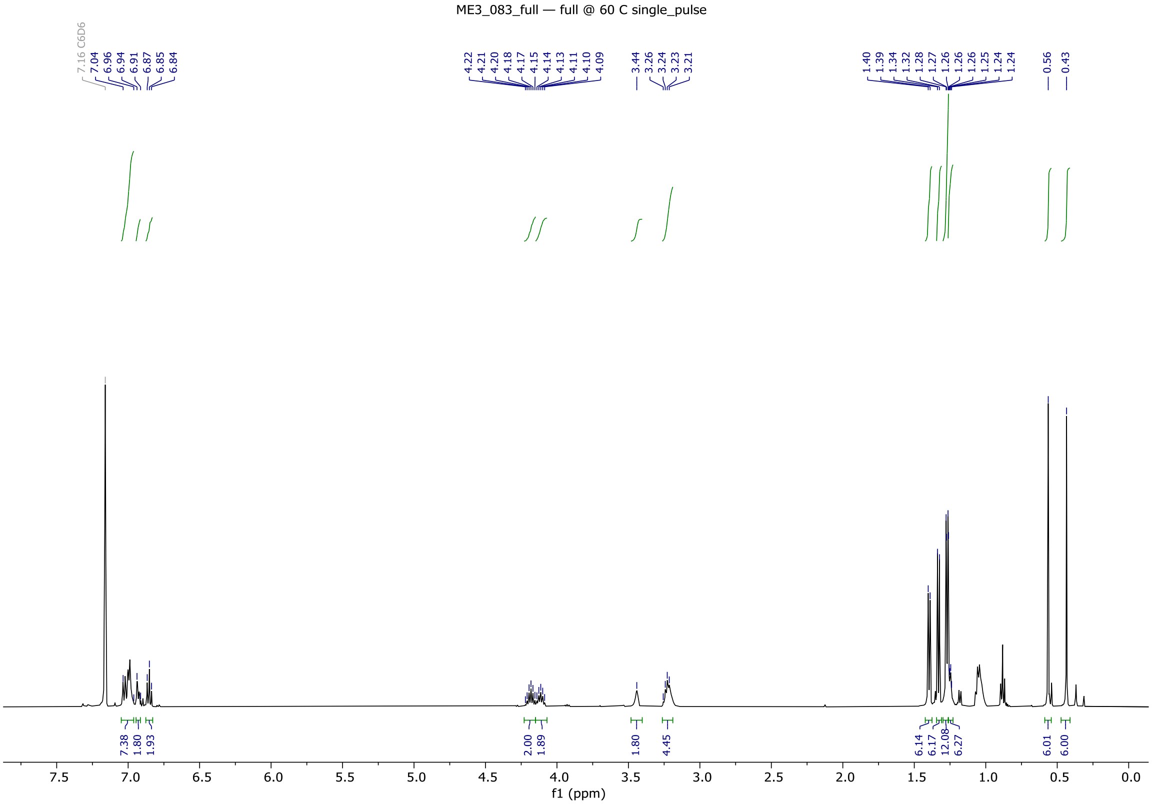

Figure S18. ^{1}H NMR spectrum of **1-Na·(Et$_2$O)** in benzene-d_6.

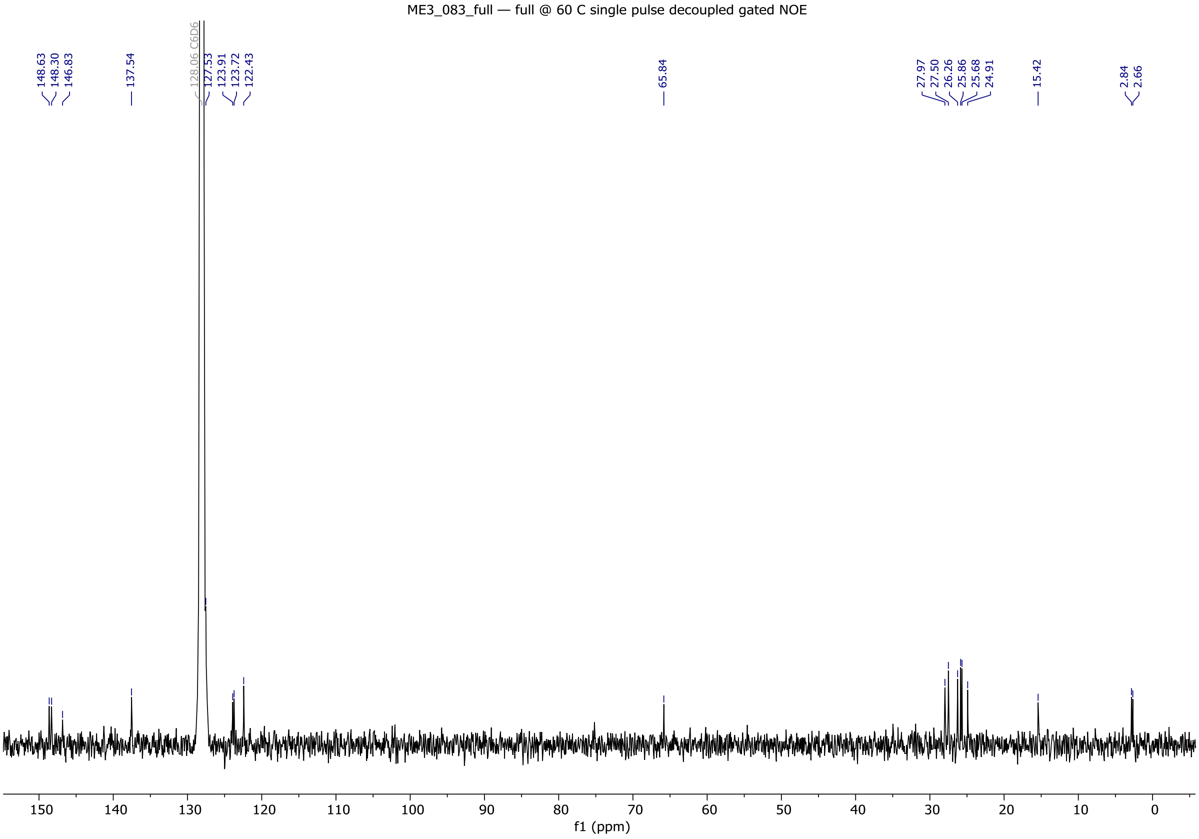

Figure S19. ^{13}C{^{1}H} NMR spectrum of **1-Na·(Et$_2$O)** in benzene-d_6.

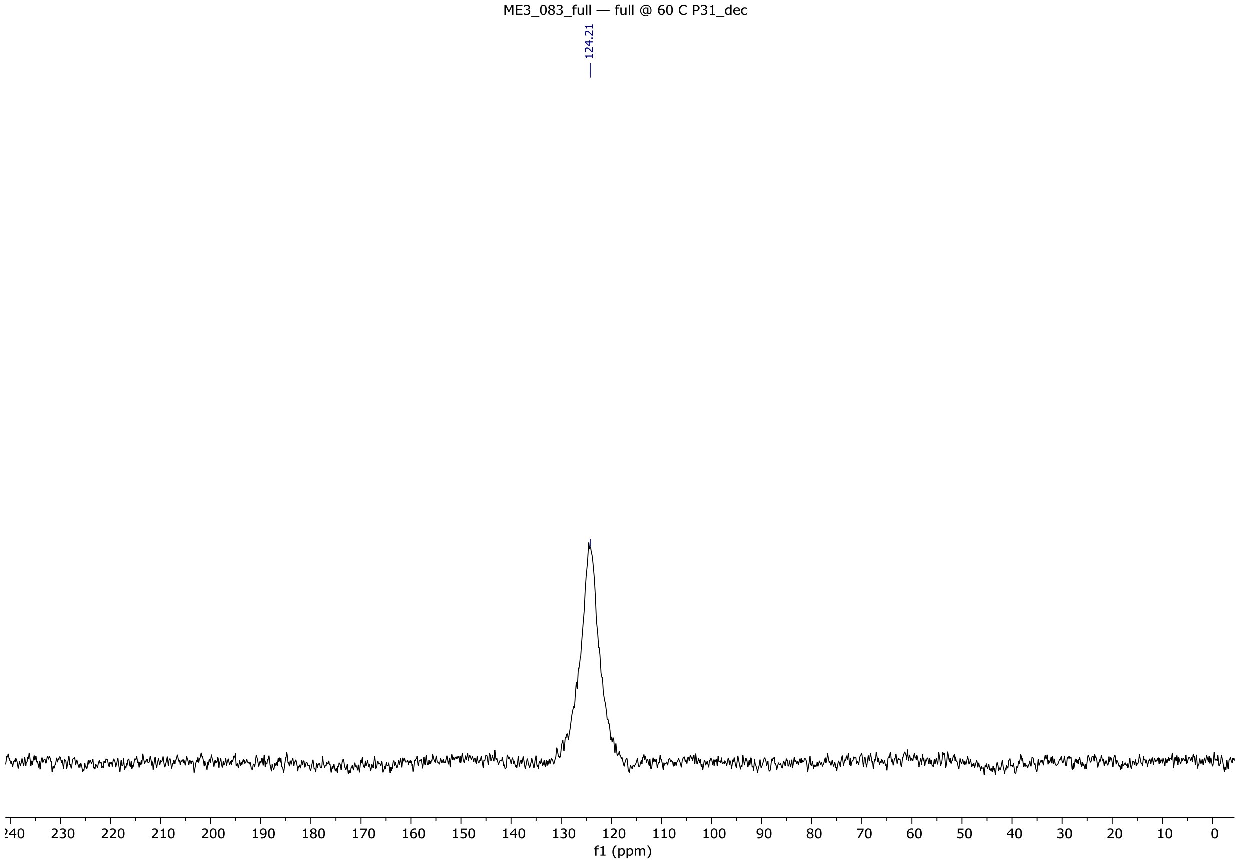

Figure S20. $^{27}Al\{^{1}H\}$ NMR spectrum of **1-Na·(Et$_2$O)** in benzene-*d*$_6$.

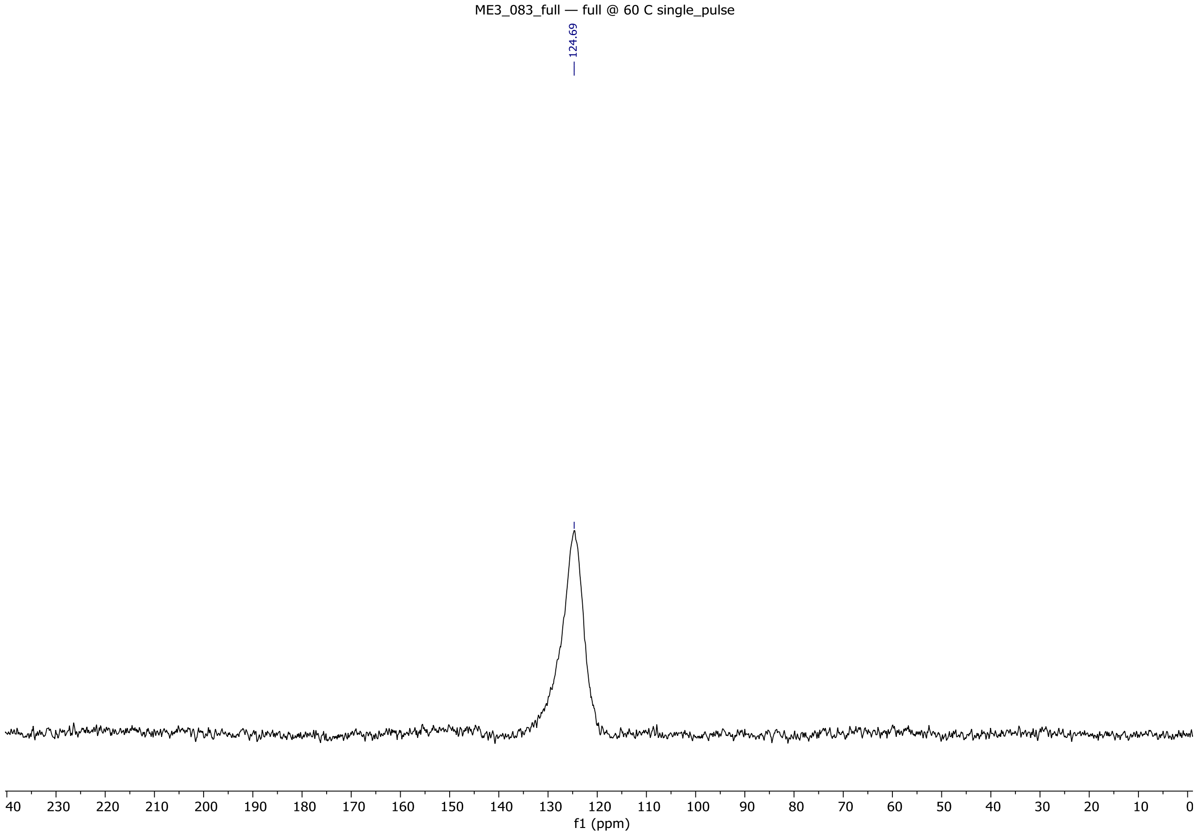

Figure S21. ^{27}Al NMR spectrum of **1-Na·(Et$_2$O)** in benzene-*d*$_6$.

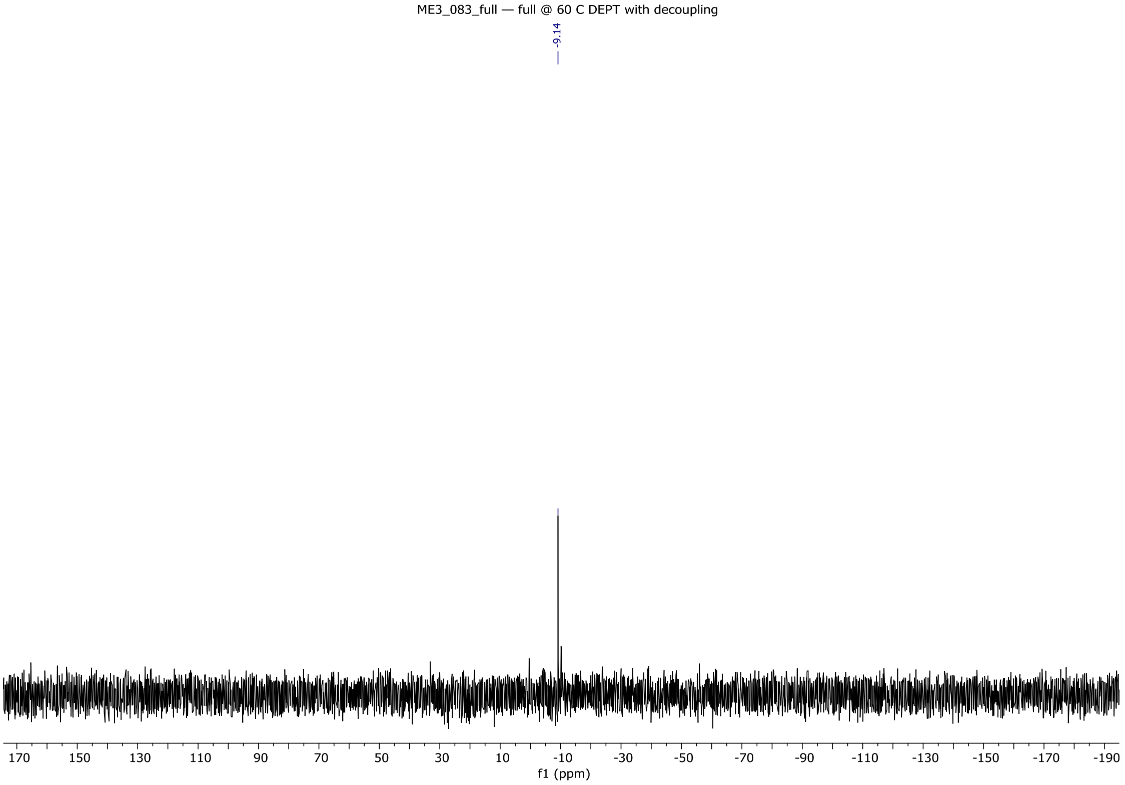

Figure S22. ^{29}Si DEPT NMR spectrum of **1-Na·(Et$_2$O)** in benzene-d_6.

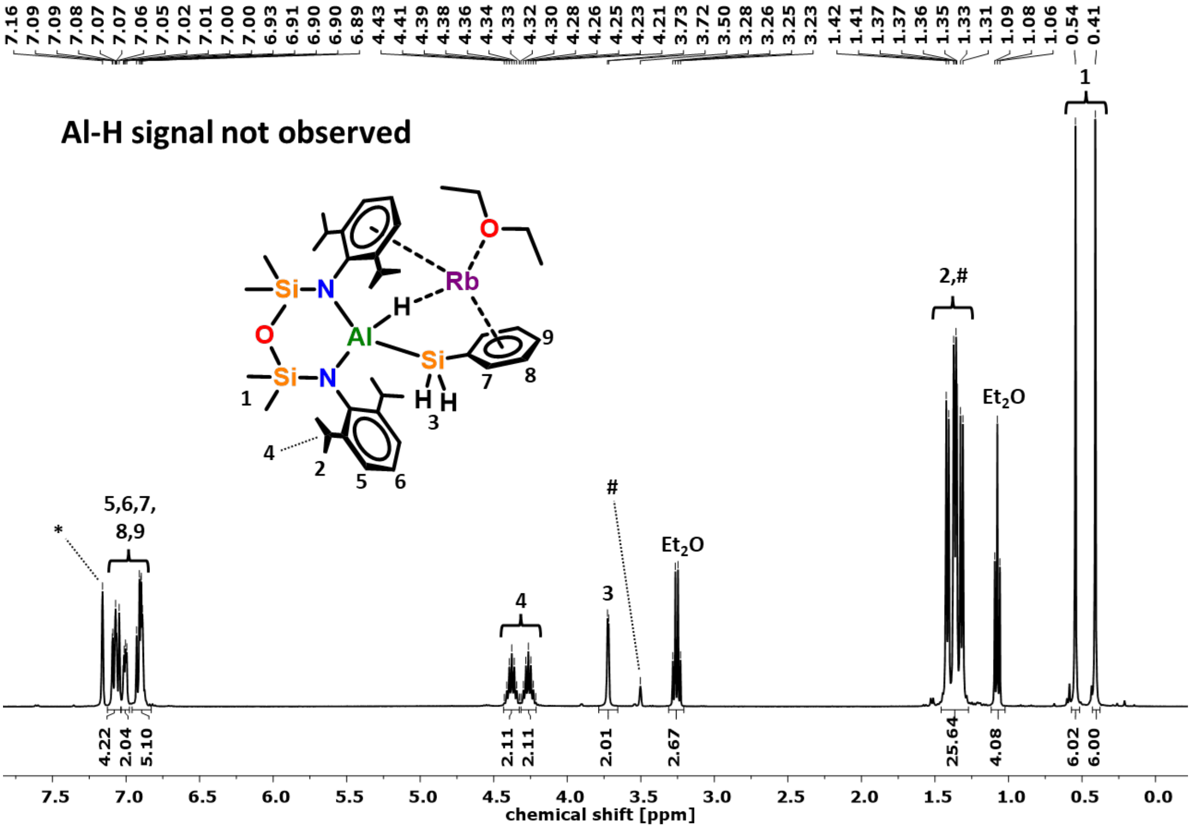

Figure S23. ^{1}H NMR spectrum of **1-Rb·(Et$_2$O)** in a mixture of benzene-d_6:THF-d_8 (10:1). Residual signal for deuterated solvents marked with * and #.

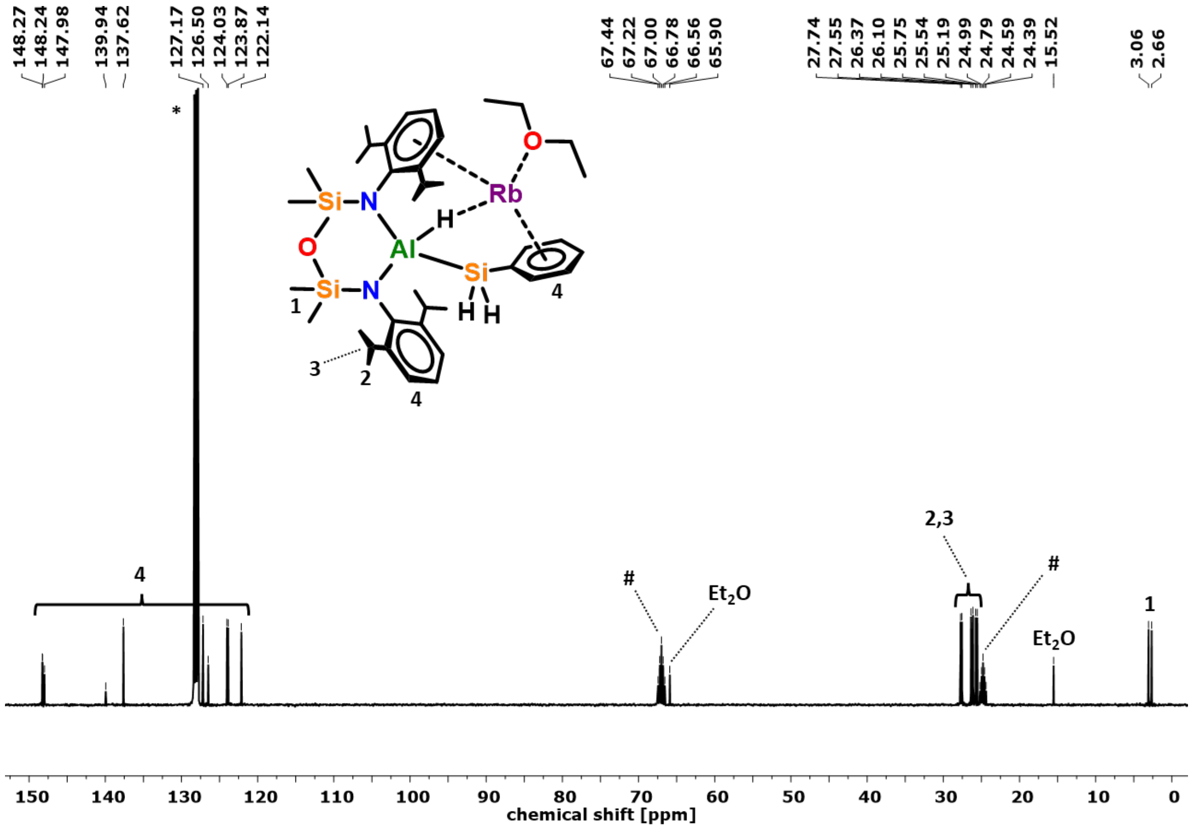

Figure S24. ^{13}C NMR spectrum of **1-Rb·(Et$_2$O)** in a mixture of benzene-d_6:THF-d_8 (10:1). Residual signal for deuterated solvents marked with * and #.

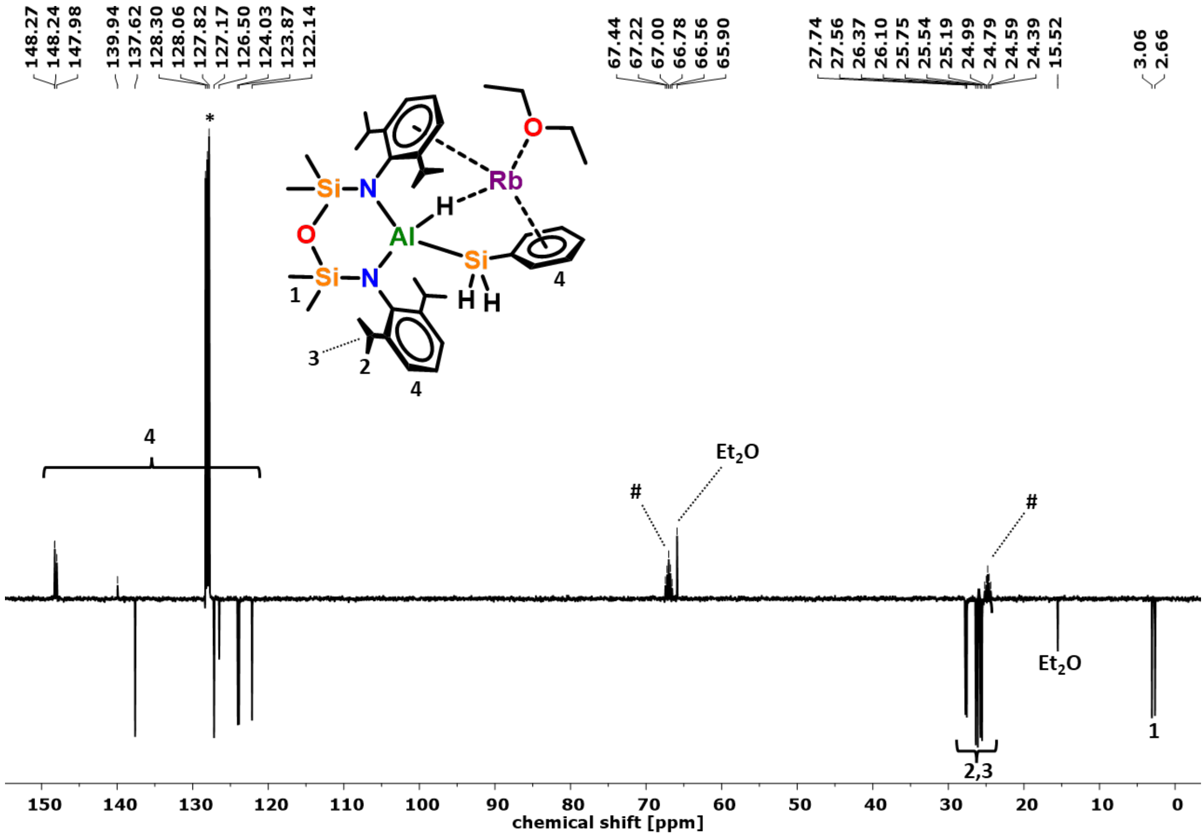

Figure S25. ^{13}C-DEPTQ NMR spectrum of **1-Rb·(Et$_2$O)** in a mixture of benzene-d_6:THF-d_8 (10:1). Residual signal for deuterated solvents marked with * and #.

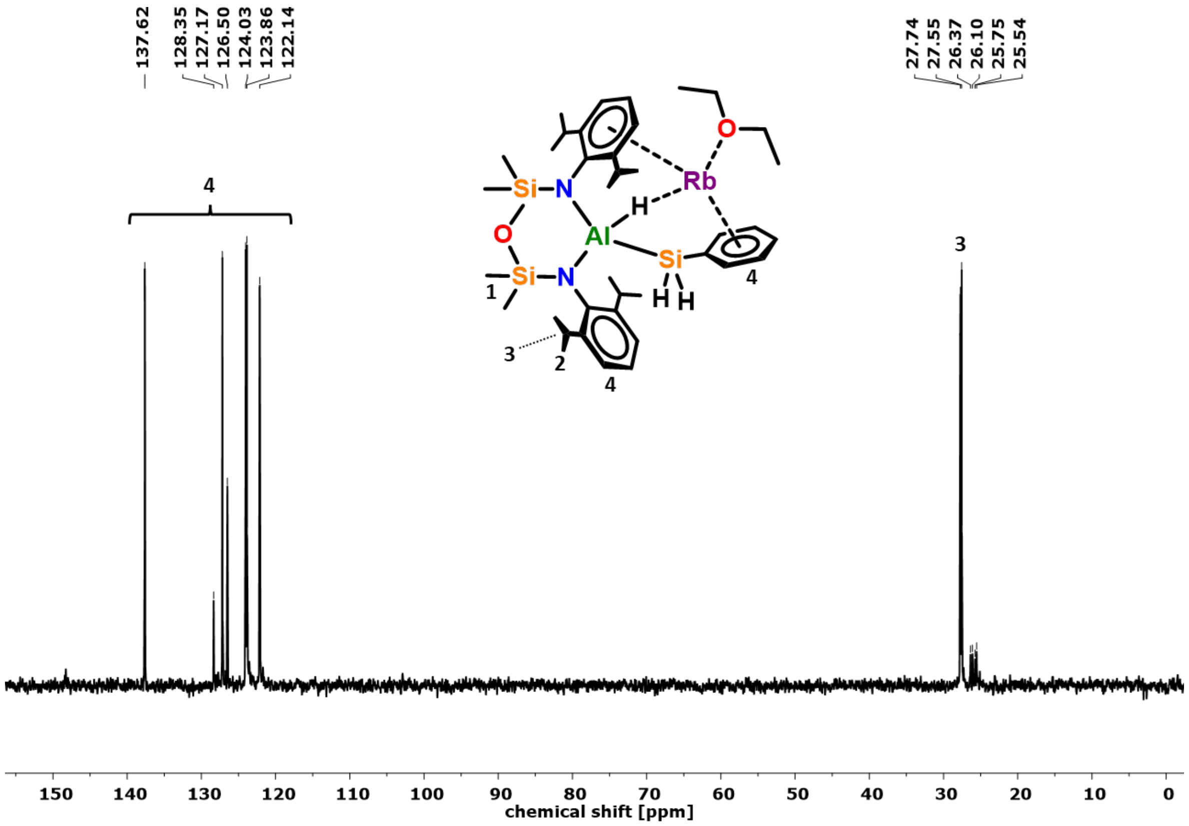

Figure S26. ^{13}C-DEPT-90 NMR spectrum of **1-Rb·(Et$_2$O)** in a mixture of benzene-d_6:THF-d_8 (10:1).

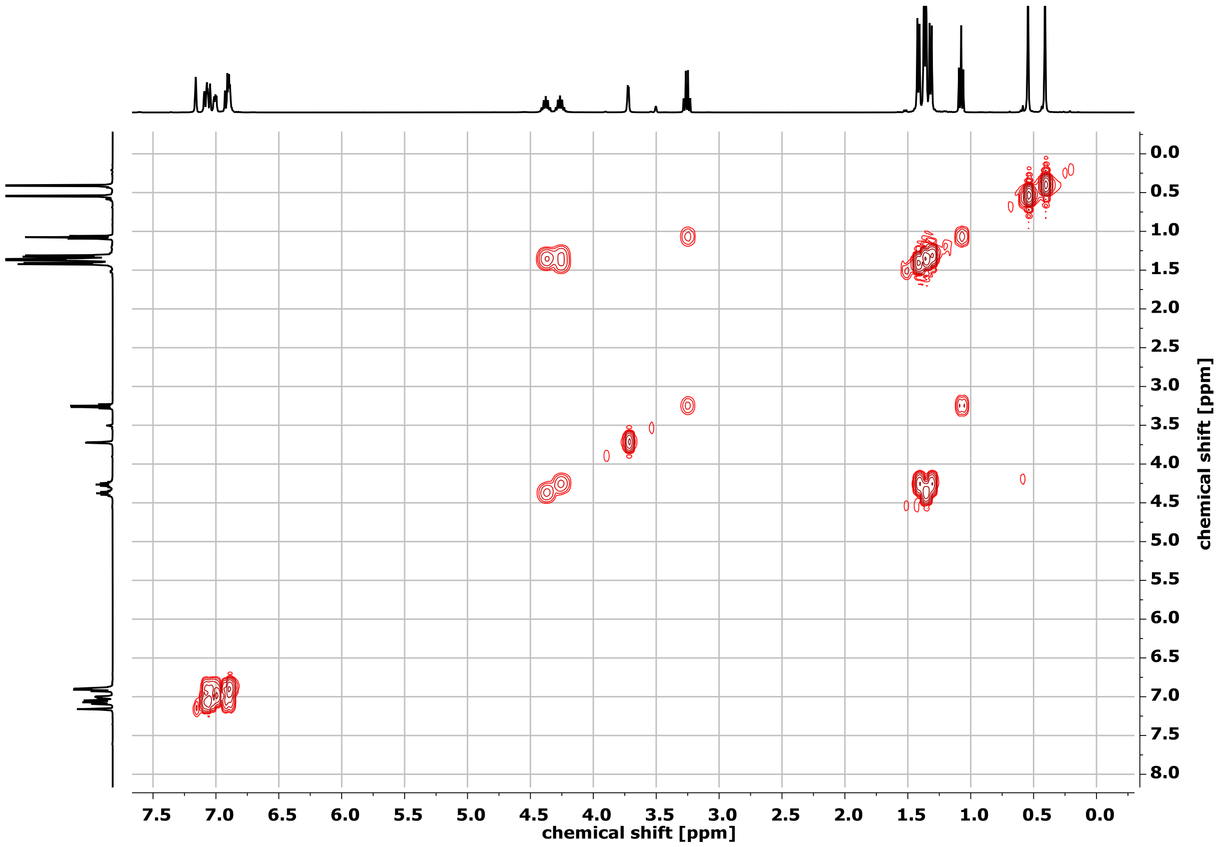

Figure S27. ^{1}H^{1}H-COSY NMR spectrum of **1-Rb·(Et$_2$O)** in a mixture of benzene-d_6:THF-d_8 (10:1).

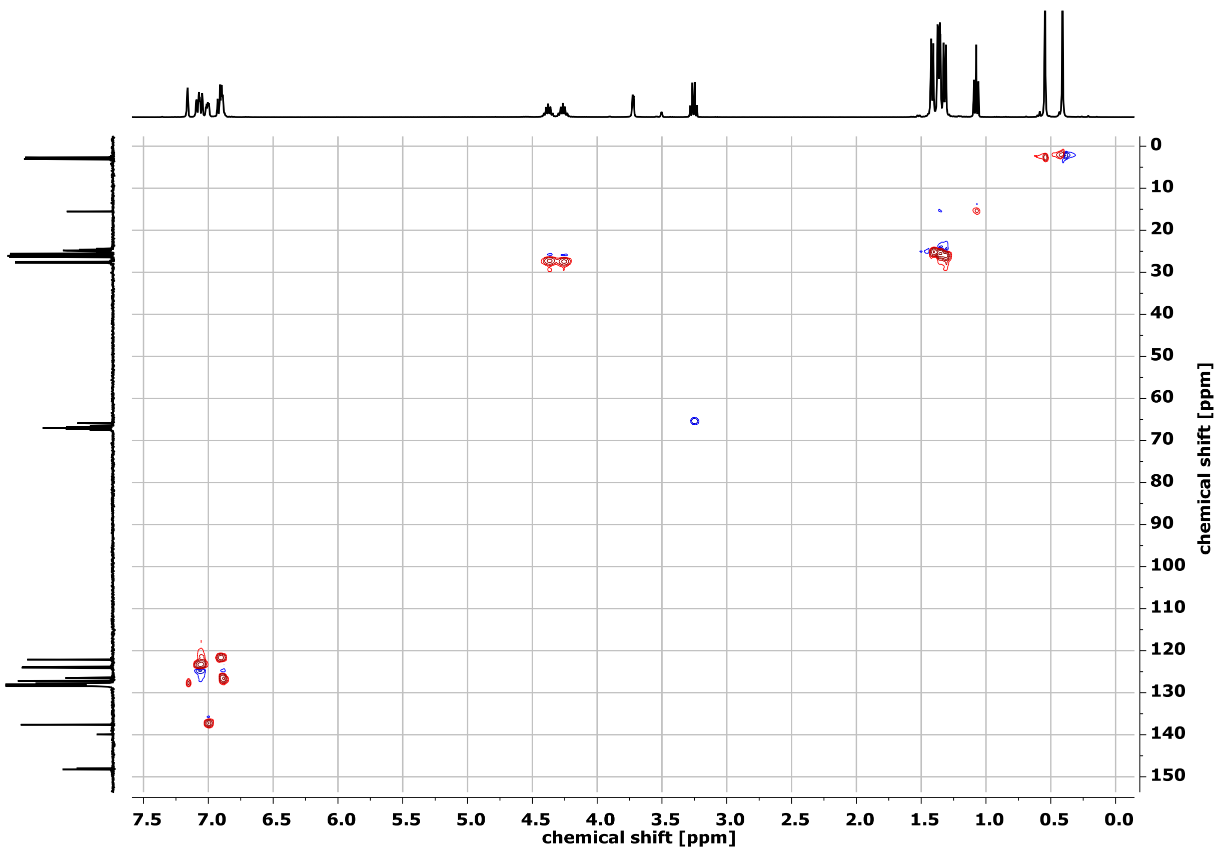

Figure S28. ^{1}H^{13}C-HSQC NMR spectrum of **1-Rb·(Et₂O)** in a mixture of benzene-d_6:THF-d_8 (10:1).

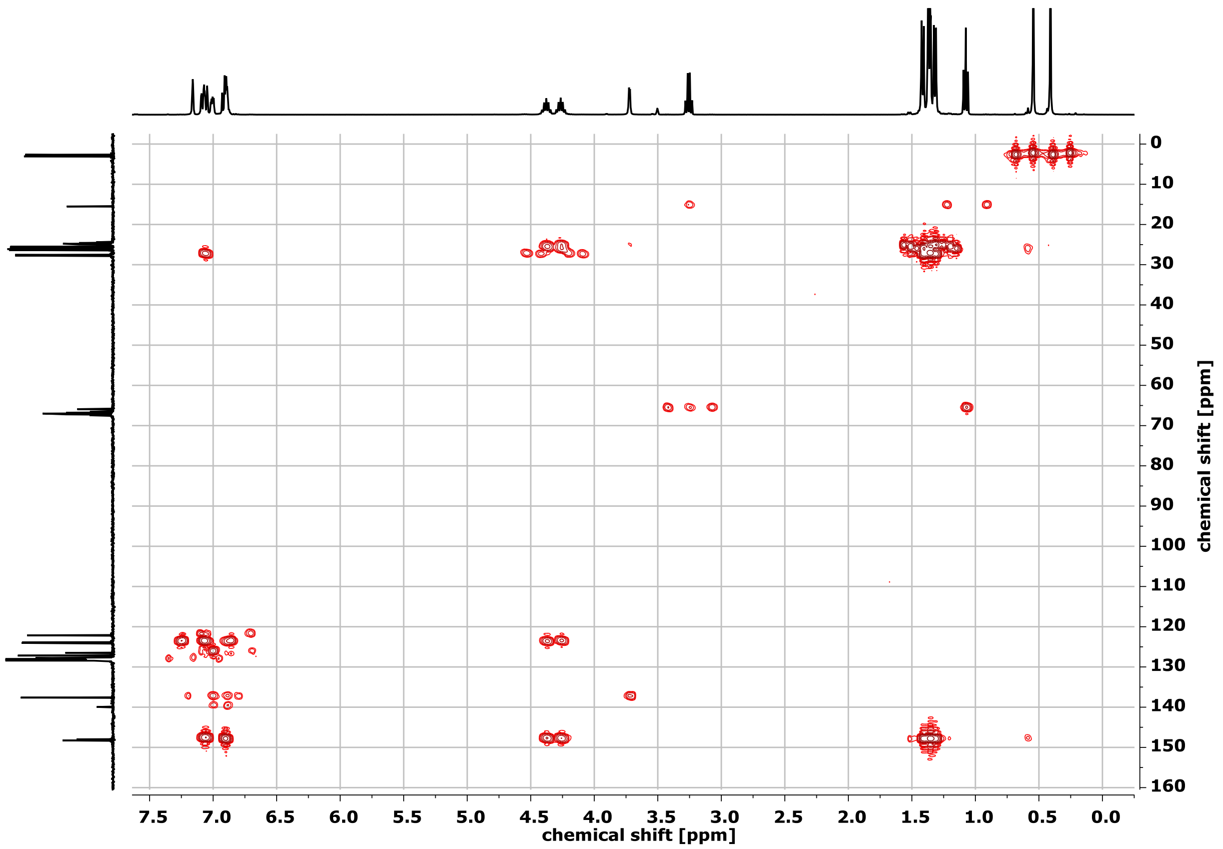

Figure S29. ^{1}H^{13}C-HMBC NMR spectrum of **1-Rb·(Et₂O)** in a mixture of benzene-d_6:THF-d_8 (10:1).

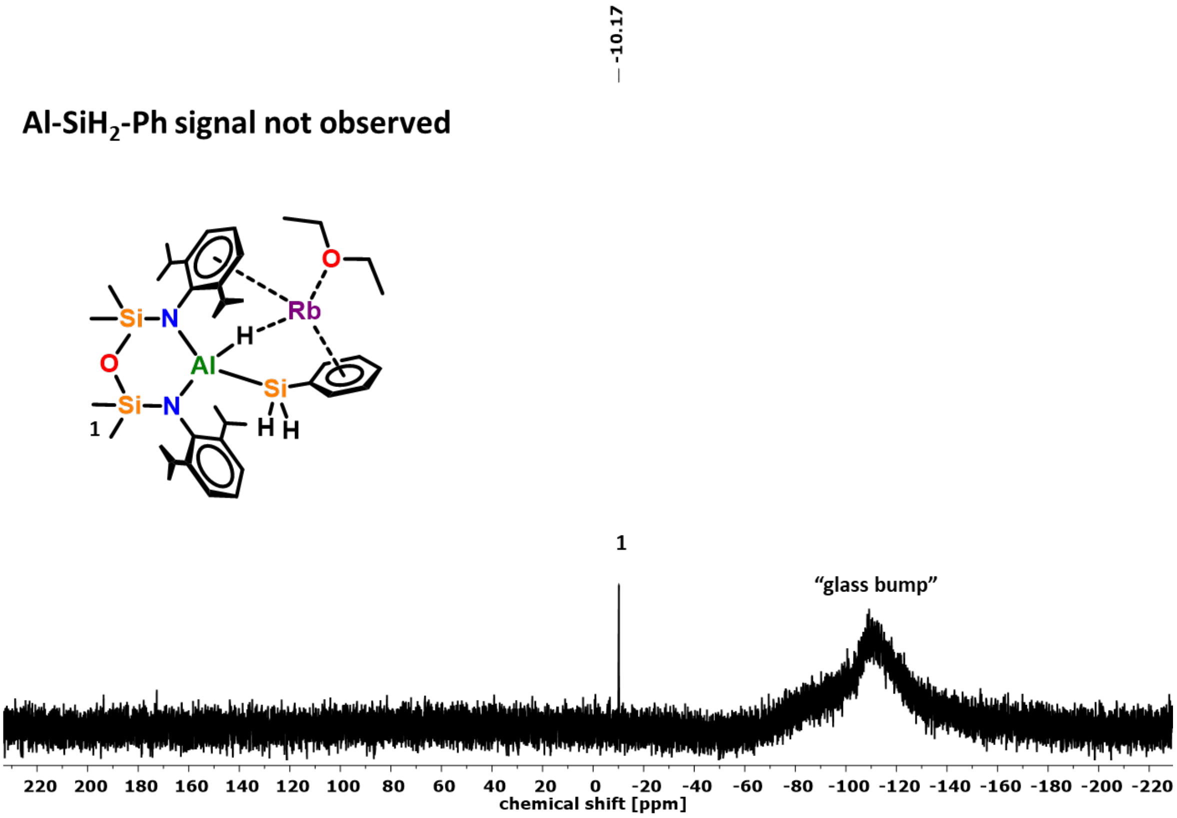

Figure S30. ^{29}Si NMR spectrum of **1-Rb·(Et$_2$O)** in a mixture of benzene-d_6:THF-d_8 (10:1).

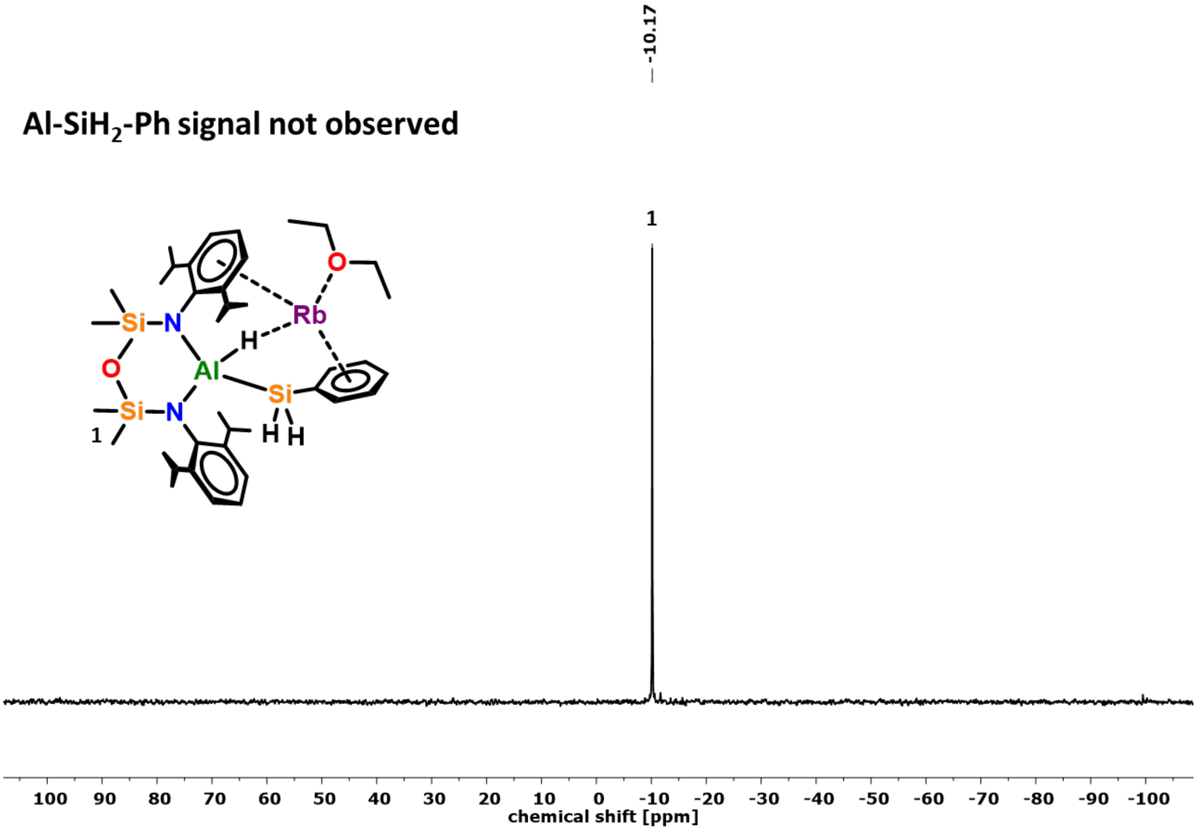

Figure S31. ^{29}Si-INEPT NMR spectrum of **1-Rb·(Et$_2$O)** in a mixture of benzene-d_6:THF-d_8 (10:1).

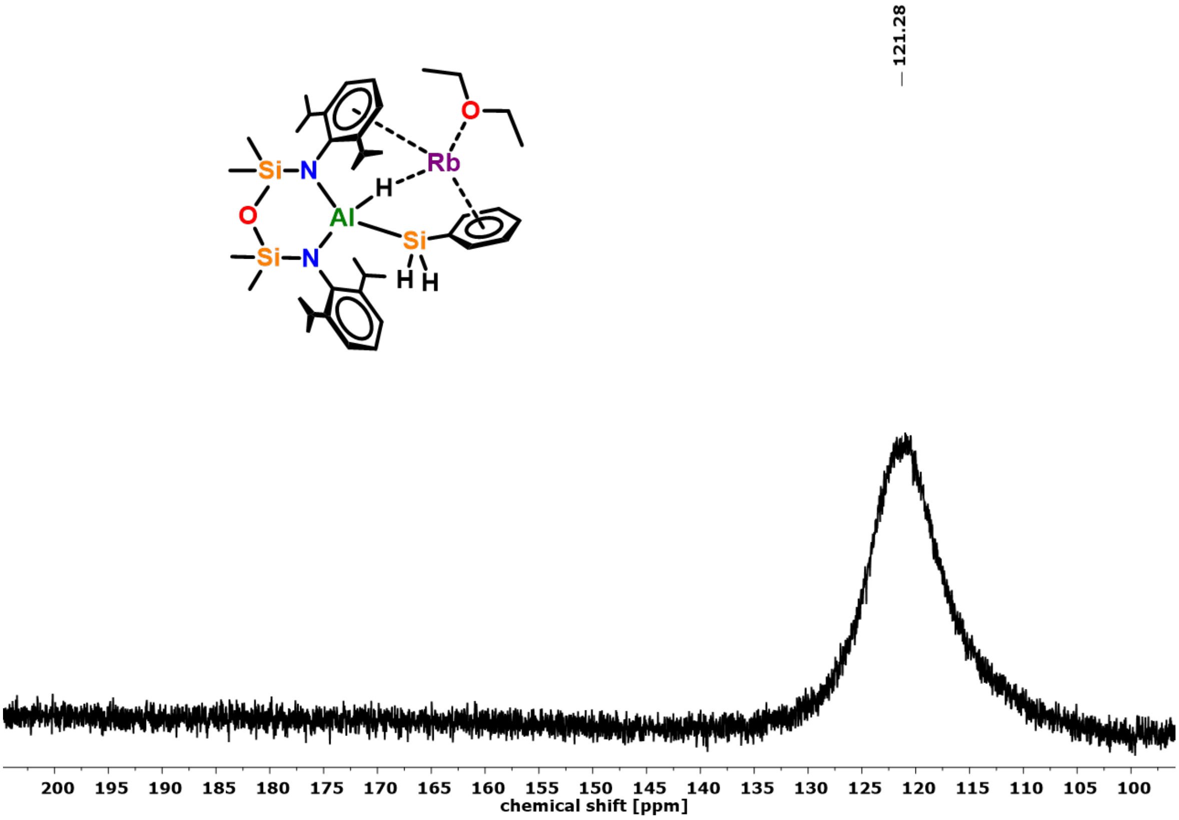

Figure S32. ^{27}Al NMR spectrum of **1-Rb·(Et$_2$O)** in a mixture of benzene-*d$_6$*:THF-*d$_8$* (10:1).

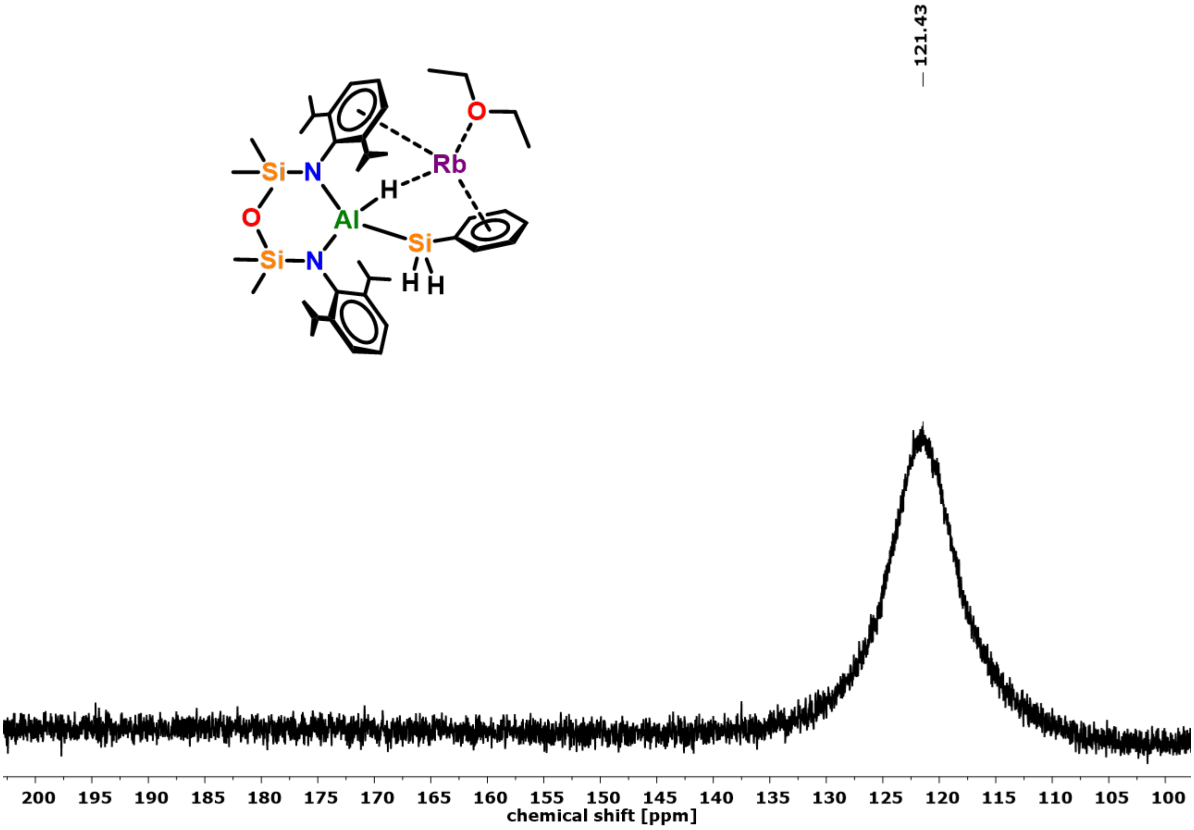

Figure S33. ^{27}Al{1H} NMR spectrum of **1-Rb·(Et$_2$O)** in a mixture of benzene-*d$_6$*:THF-*d$_8$* (10:1).

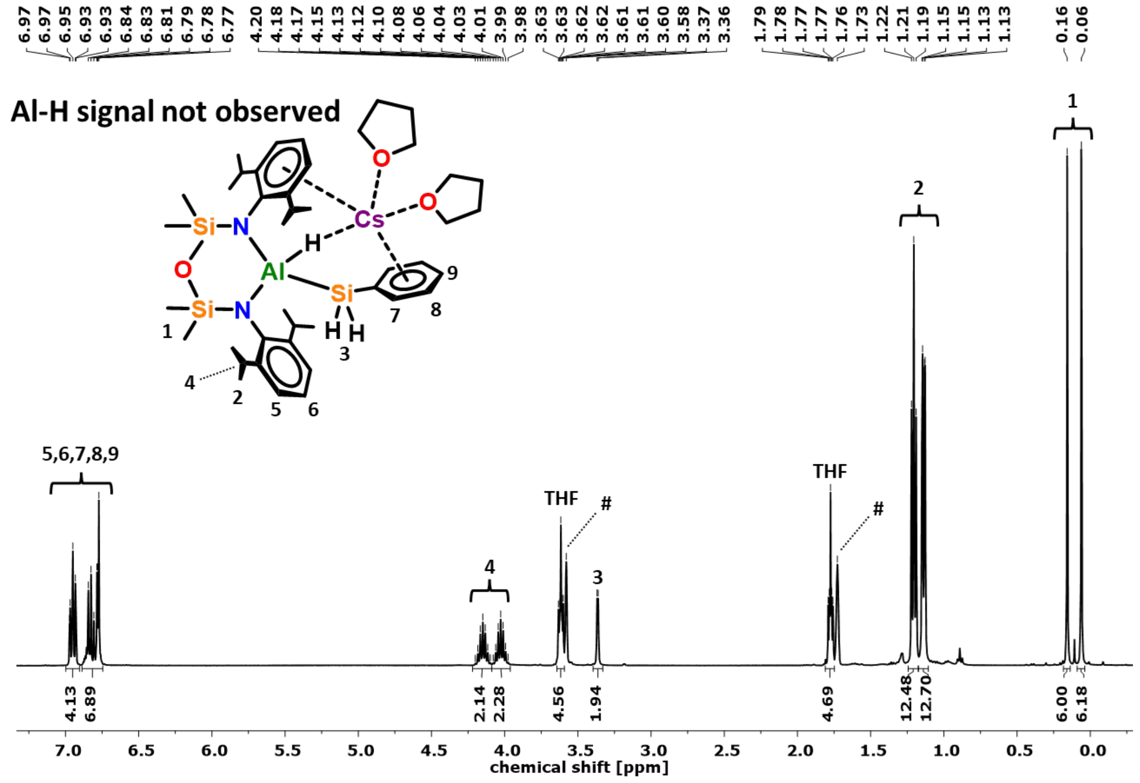

Figure S34. ^{1}H NMR spectrum of **1-Cs·(THF)$_2$** in THF-d_8. Residual signal for deuterated solvent marked with #. Note the chemical formula depicted represents that of the solid-state structure. Mismatching ^{1}H signal integrals for coordinated THF likely result from prior drying the crystalline material for NMR sample preparation under high vacuum.

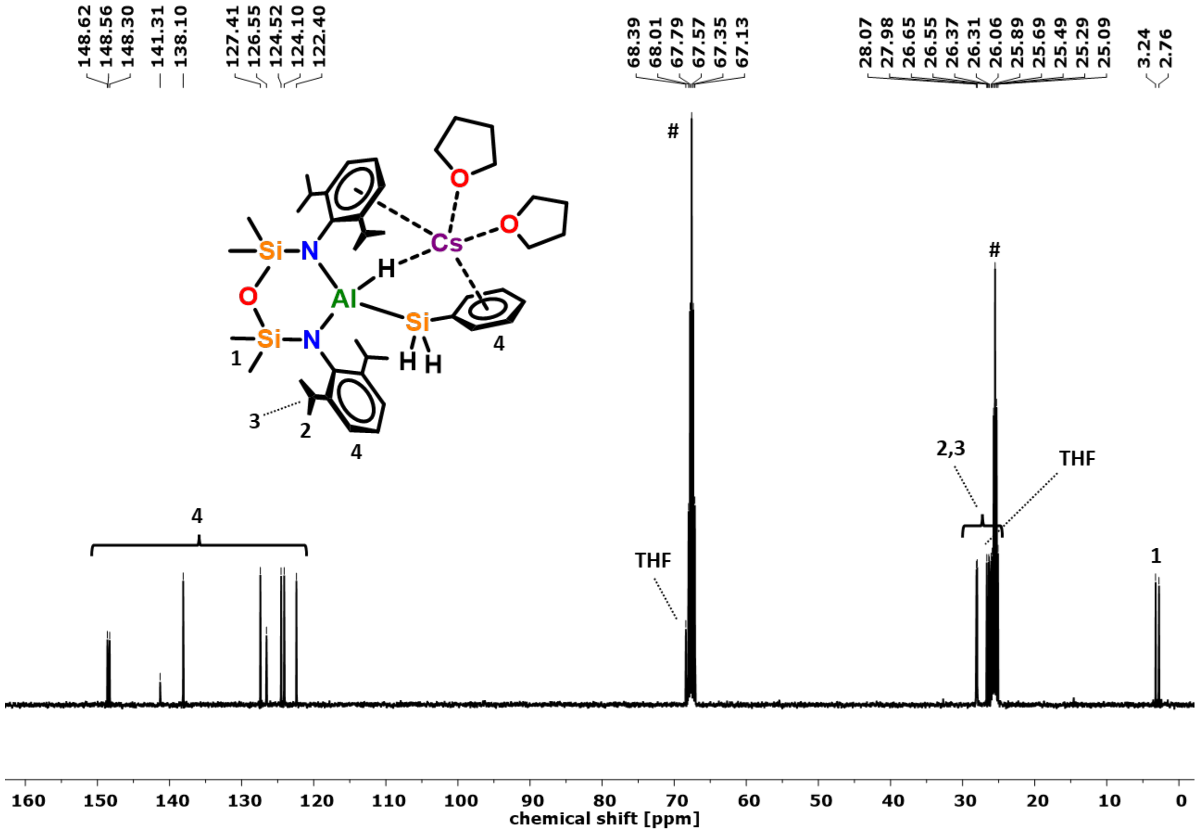

Figure S35. ^{13}C NMR spectrum of **1-Cs·(THF)$_2$** in THF-d_8. Residual signal for deuterated solvent marked with #.

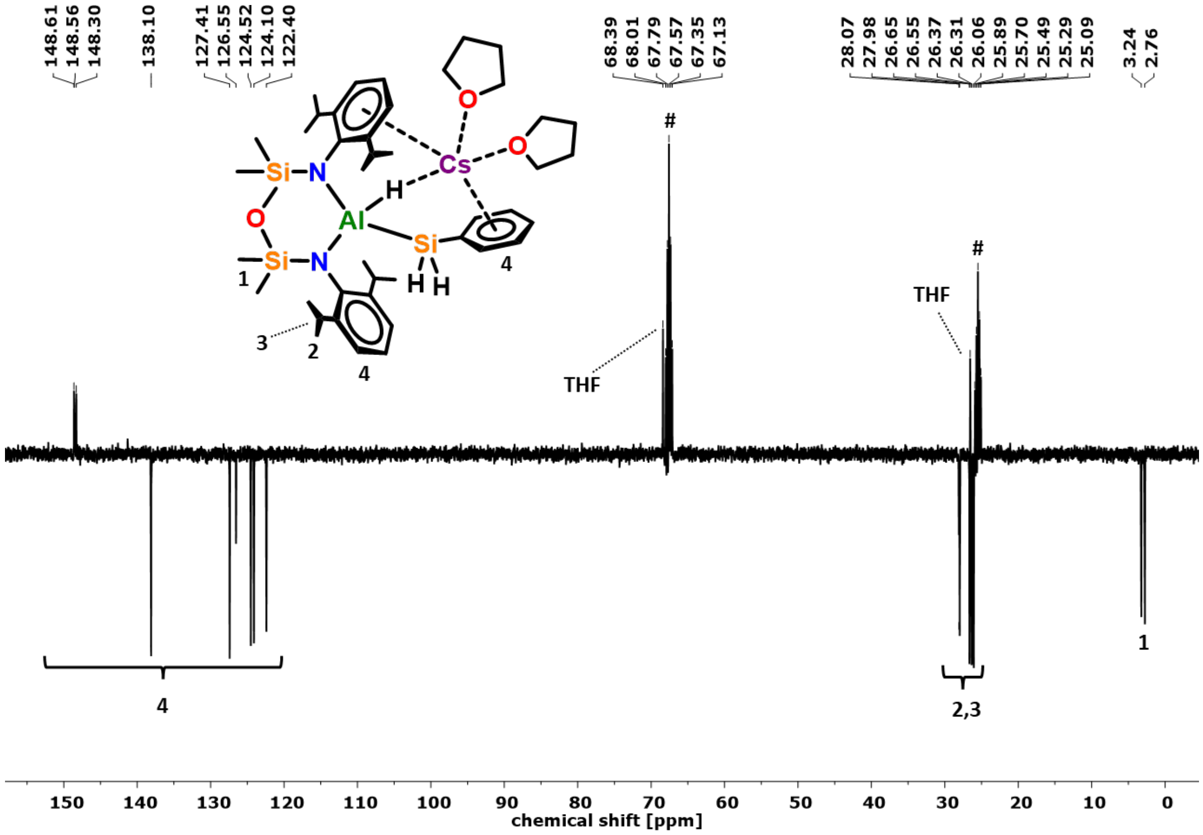

Figure S36. ^{13}C-DEPTQ NMR spectrum of **1-Cs·(THF)$_2$** in THF-d_8. Residual signal for deuterated solvent marked with #.

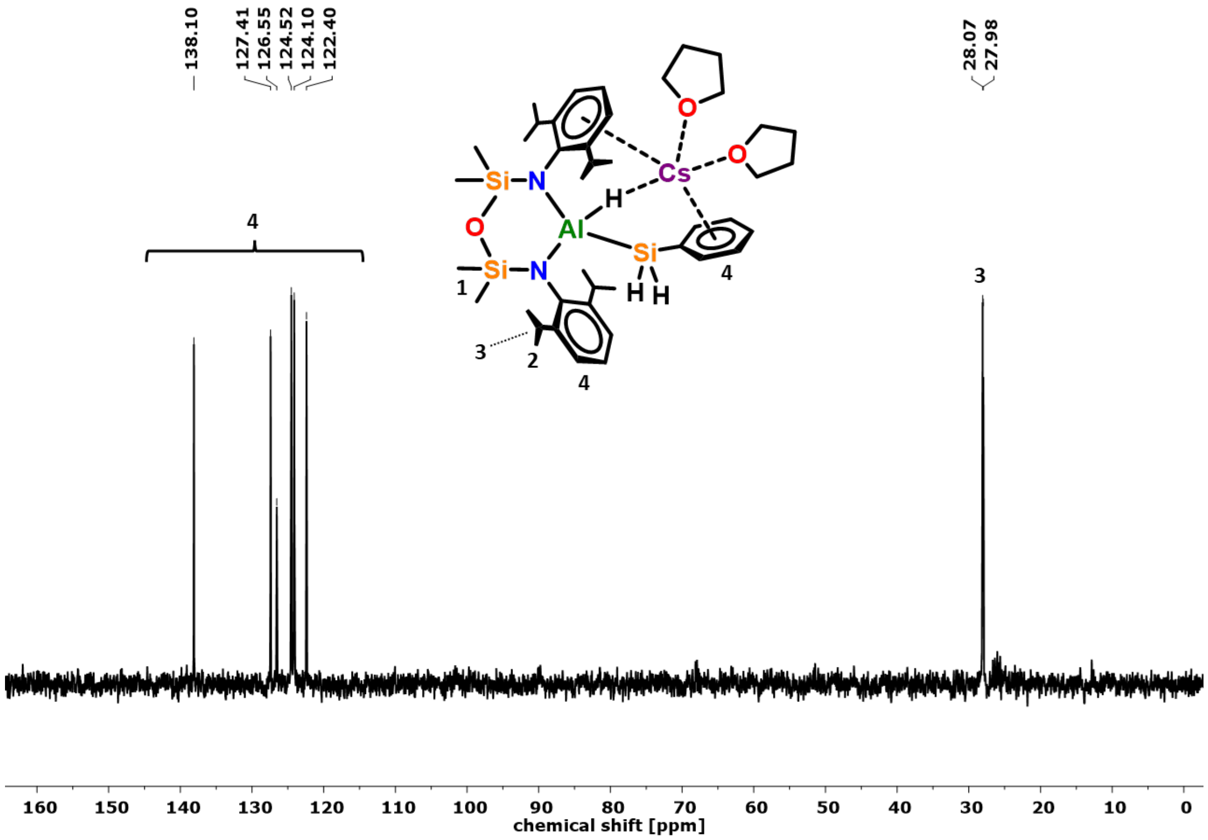

Figure S37. ^{13}C-DEPT-90 NMR spectrum of **1-Cs·(THF)$_2$** in THF-d_8.

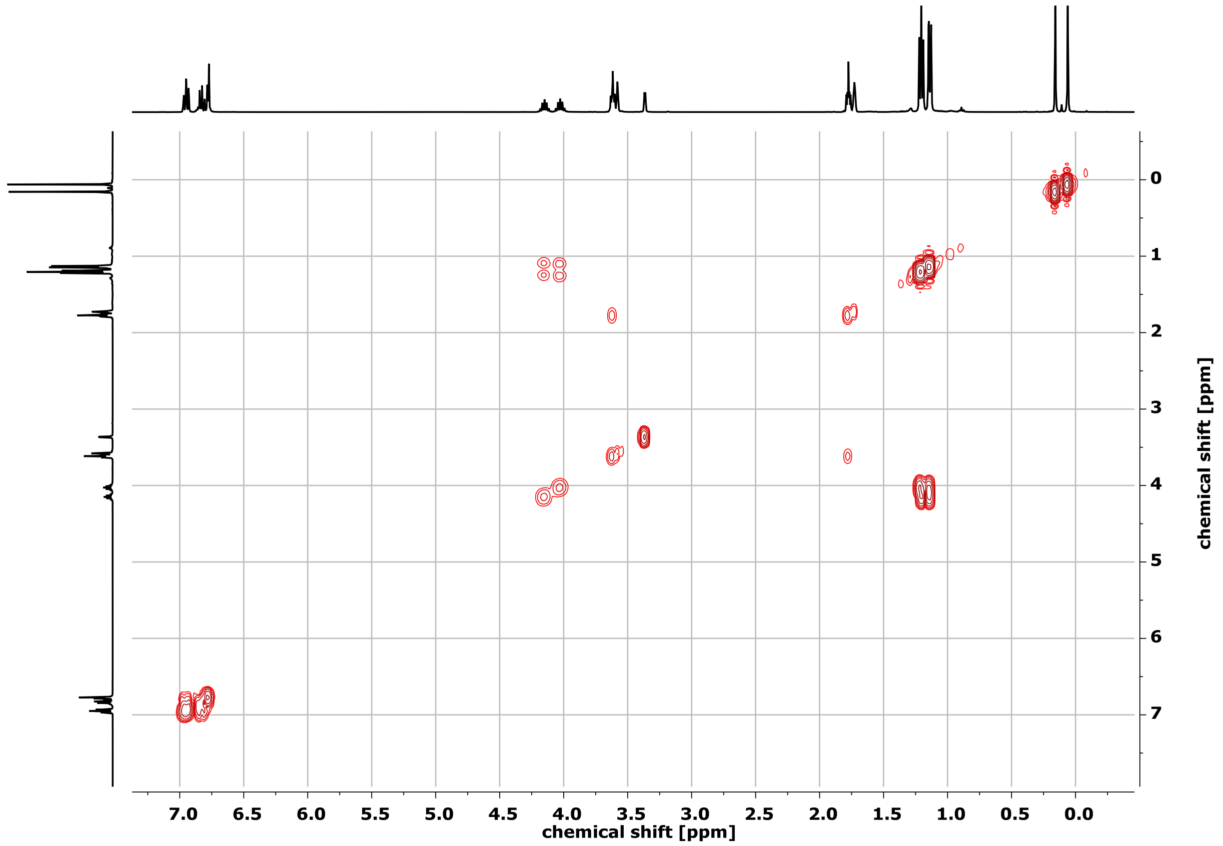

Figure S38. ^{1}H^{1}H-COSY NMR spectrum of **1-Cs·(THF)₂** in THF-*d₈*.

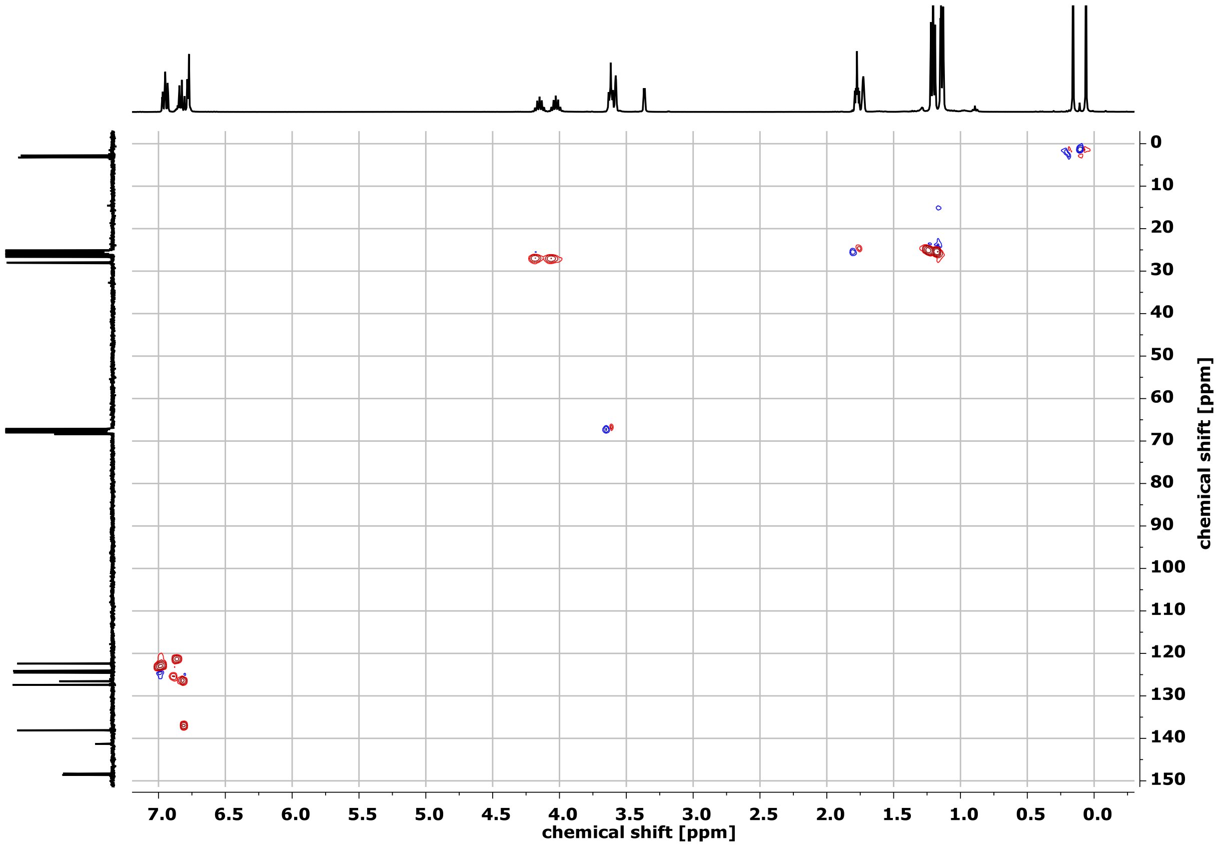

Figure S39. ^{1}H^{13}C-HSQC NMR spectrum of **1-Cs·(THF)$_2$** in THF-*d$_8$*.

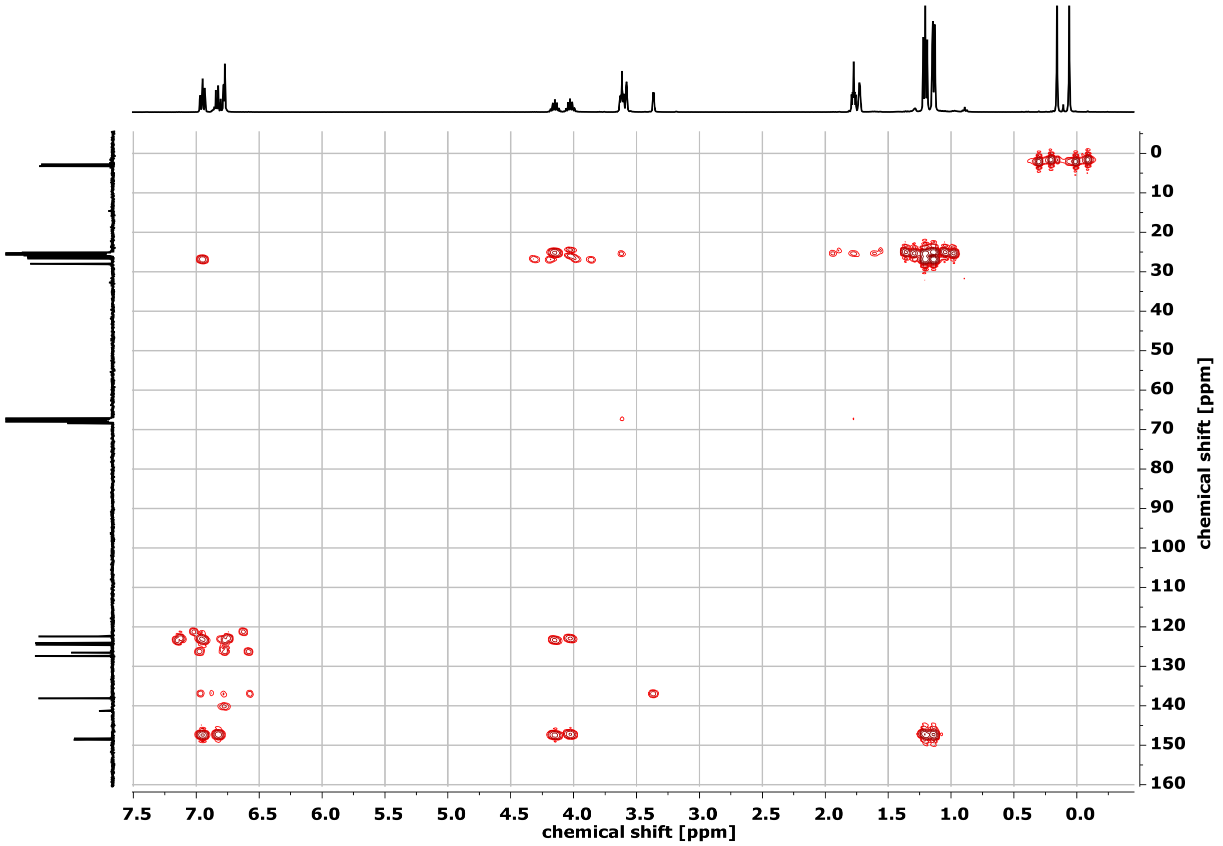

Figure S40. $^1H^{13}C$-HMBC NMR spectrum of **1-Cs·(THF)₂** in THF-*d₈*.

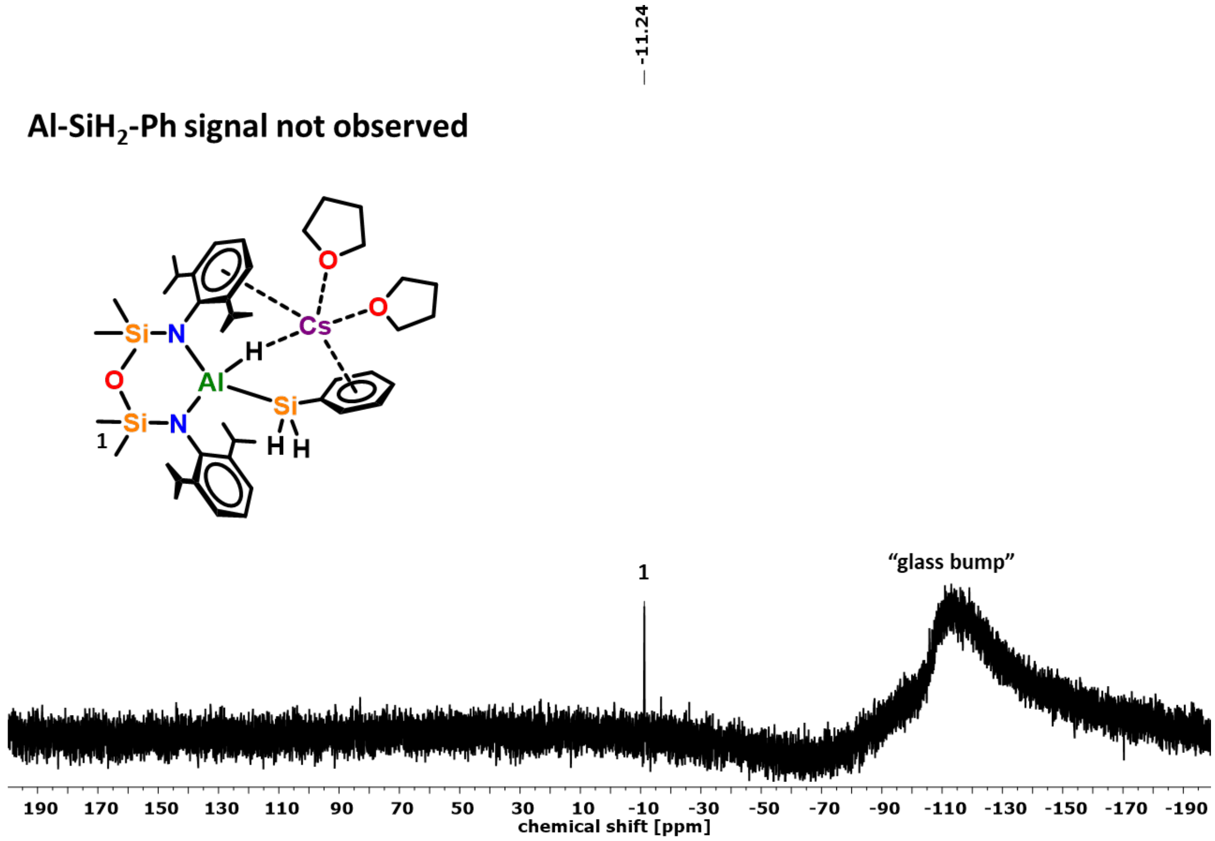

Figure S41. ^{29}Si NMR spectrum of **1-Cs·(THF)₂** in THF-*d₈*.

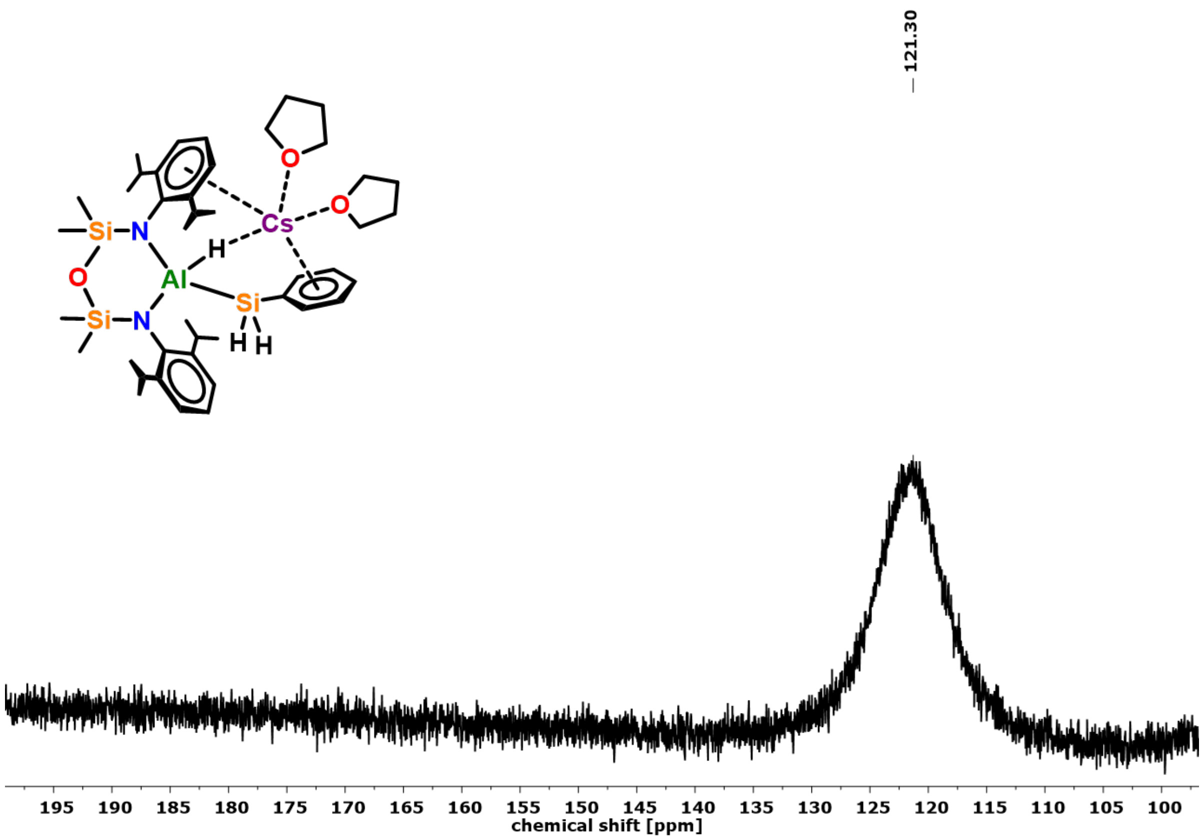

Figure S42. ^{27}Al NMR spectrum of **1-Cs·(THF)$_2$** in THF-d_8.

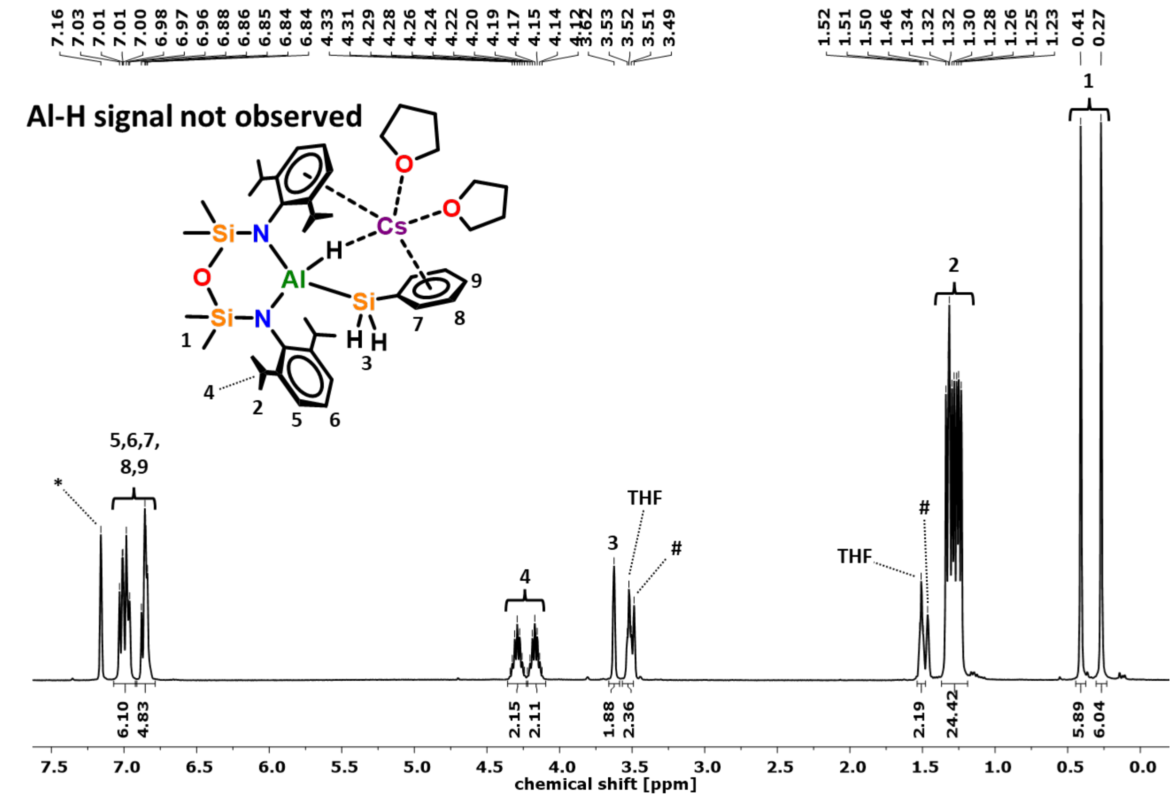

Figure S43. ^{1}H NMR spectrum of **1-Cs·(THF)$_2$** in a mixture of benzene-d_6:THF-d_8 (4:1). Residual signal for deuterated solvents marked with * and #. Note the chemical formula depicted represents that of the solid-state structure. Mismatching ^{1}H signal integrals for coordinated THF likely result from prior drying the crystalline material for NMR sample preparation under high vacuum.

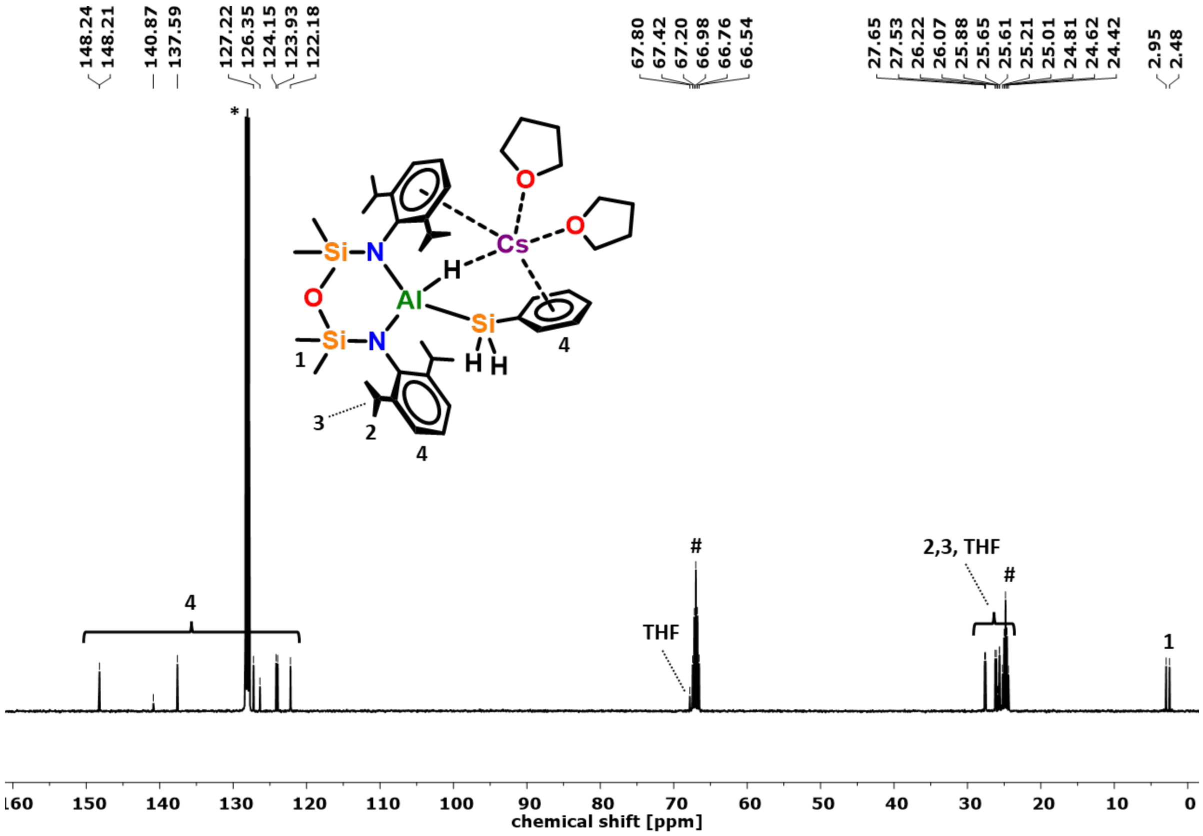

Figure S44. ^{13}C NMR spectrum of **1-Cs·(THF)₂** in a mixture of benzene-d_6:THF-d_8 (4:1). Residual signal for deuterated solvents marked with * and #.

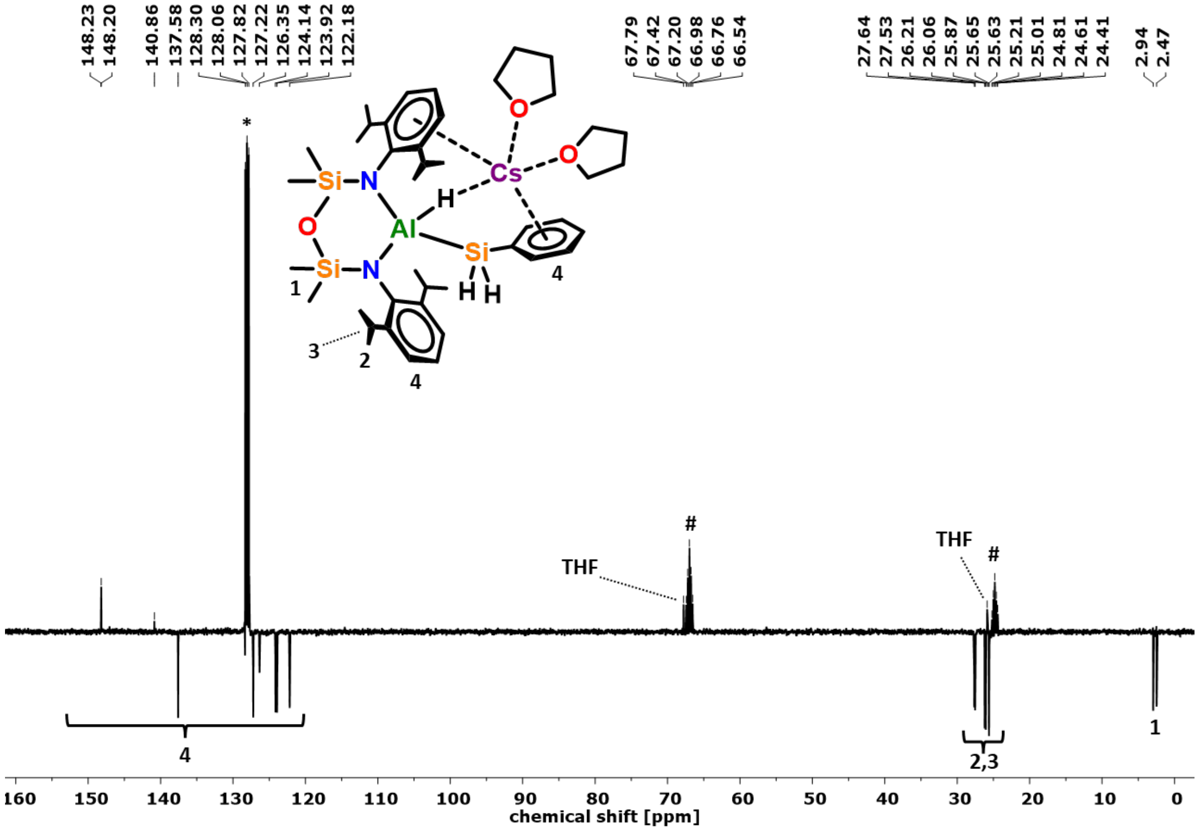

Figure S45. ^{13}C-DEPTQ NMR spectrum of **1-Cs·(THF)₂** in a mixture of benzene-d_6:THF-d_8 (4:1). Residual signal for deuterated solvents marked with * and #.

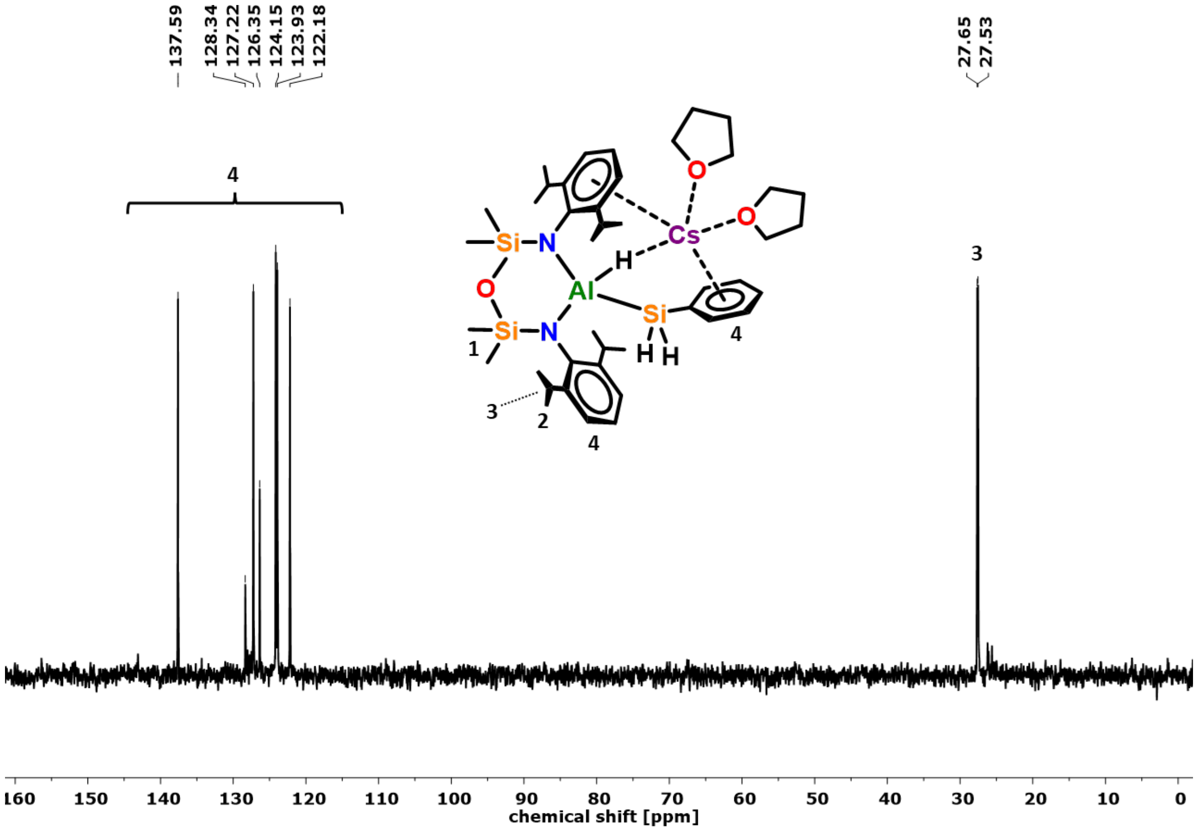

Figure S46. ^{13}C-DEPT-90 NMR spectrum of **1-Cs·(THF)₂** in a mixture of benzene-d_6:THF-d_8 (4:1).

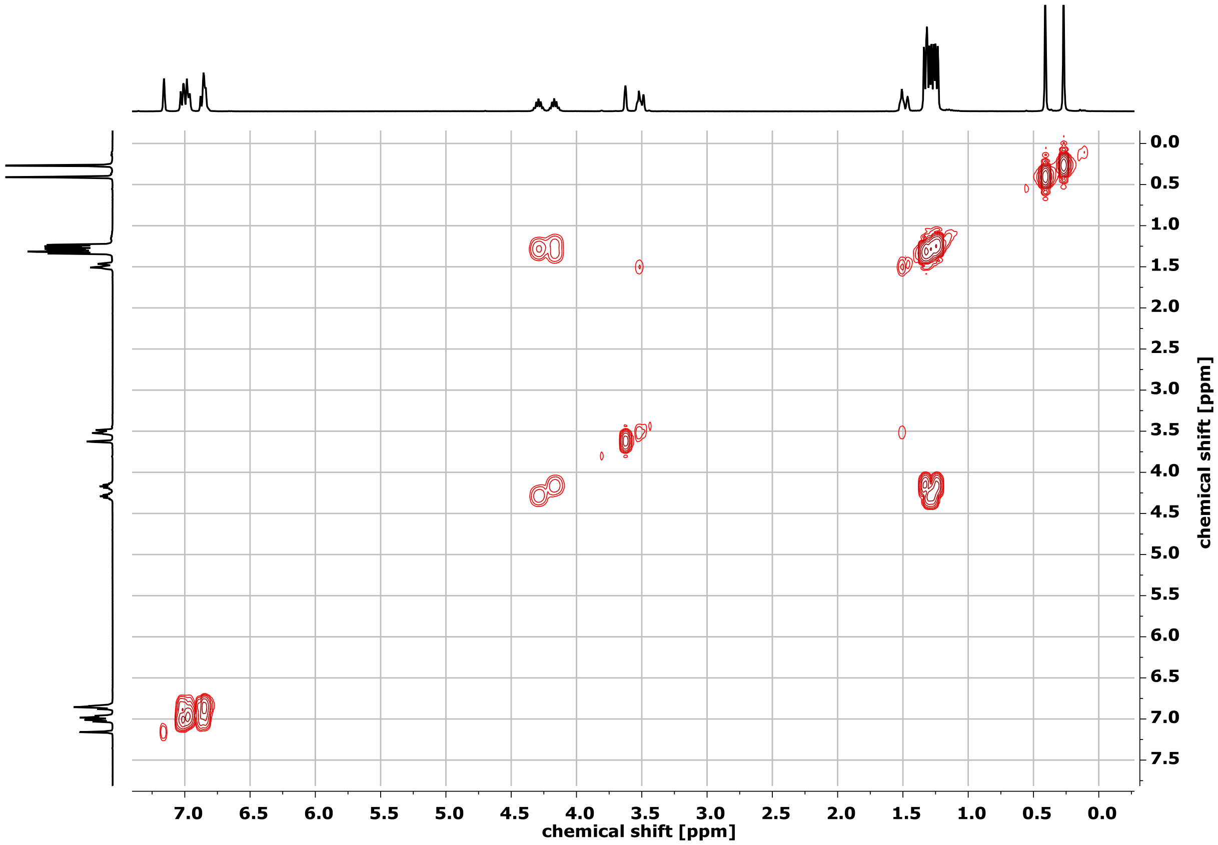

Figure S47. $^{1}H^{1}H$-COSY NMR spectrum of **1-Cs·(THF)₂** in a mixture of benzene-d_6:THF-d_8 (4:1).

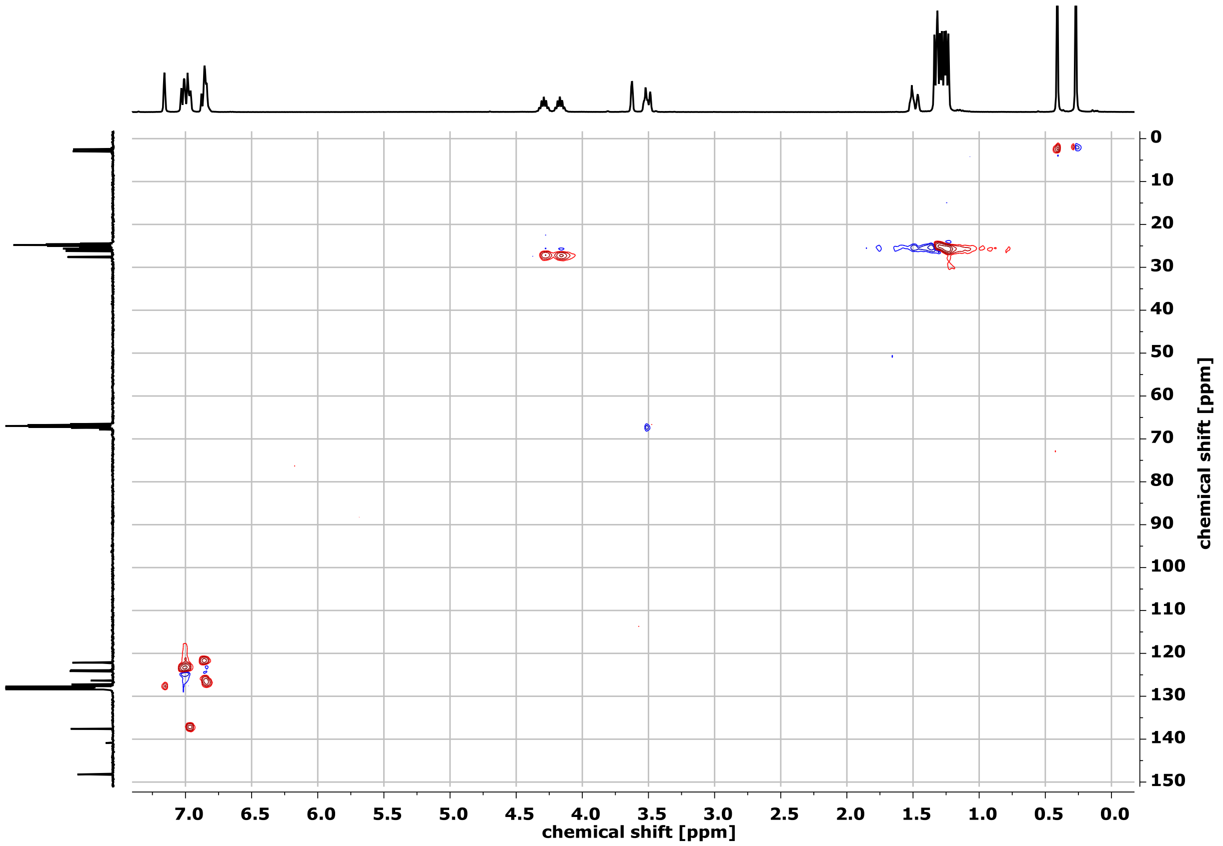

Figure S48. $^{1}H^{13}C$-HSQC NMR spectrum of **1-Cs·(THF)$_2$** in a mixture of benzene-*d$_6$*:THF-*d$_8$* (4:1).

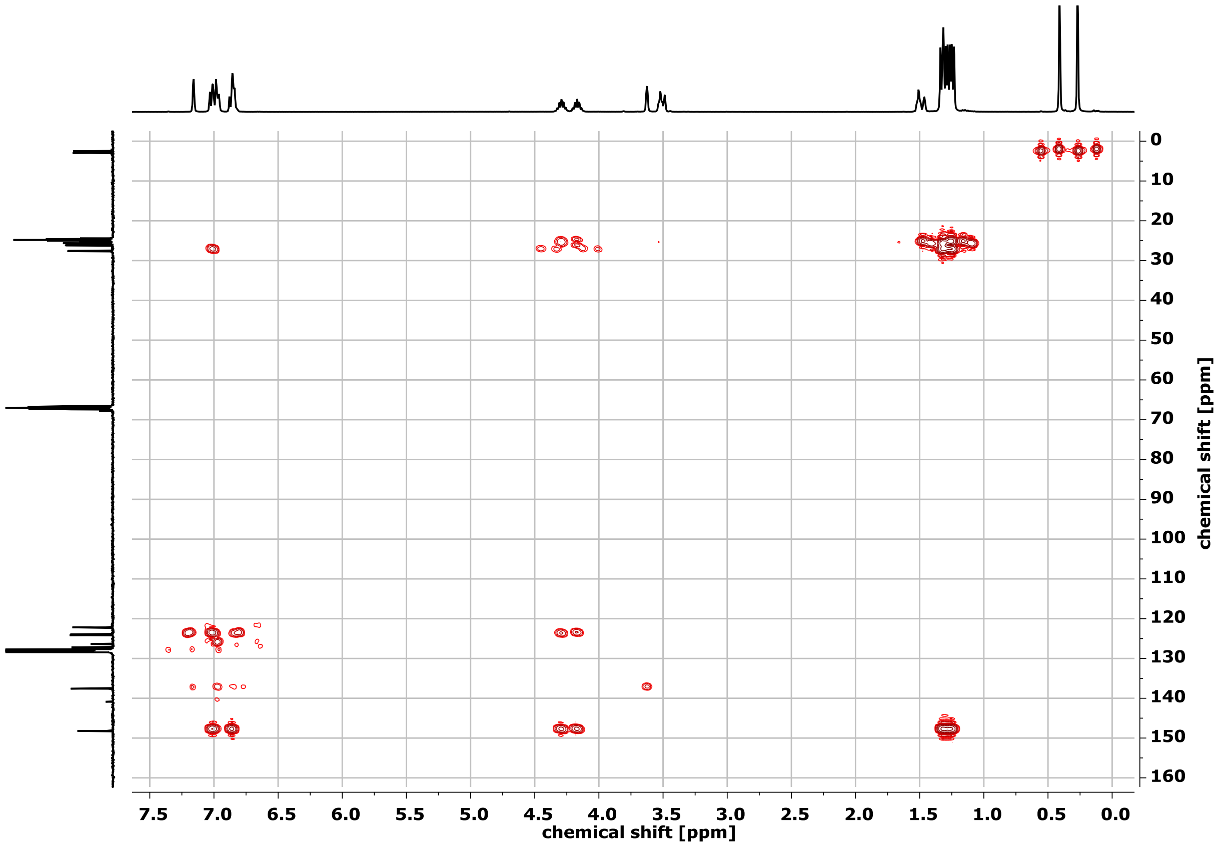

Figure S49. $^{1}H^{13}C$-HMBC NMR spectrum of **1-Cs·(THF)$_2$** in a mixture of benzene-*d$_6$*:THF-*d$_8$* (4:1).

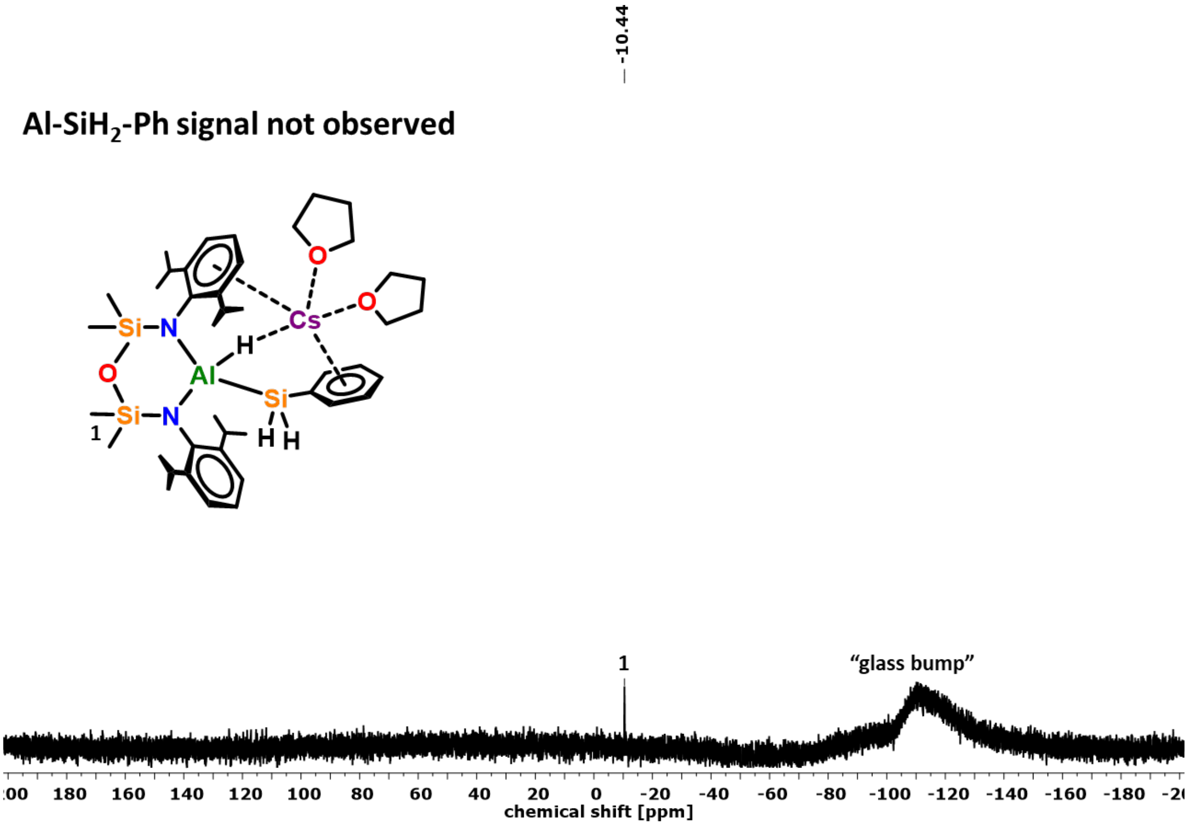

Figure S50. ^{29}Si NMR spectrum of **1-Cs·(THF)₂** in a mixture of benzene-d_6:THF-d_8 (4:1).

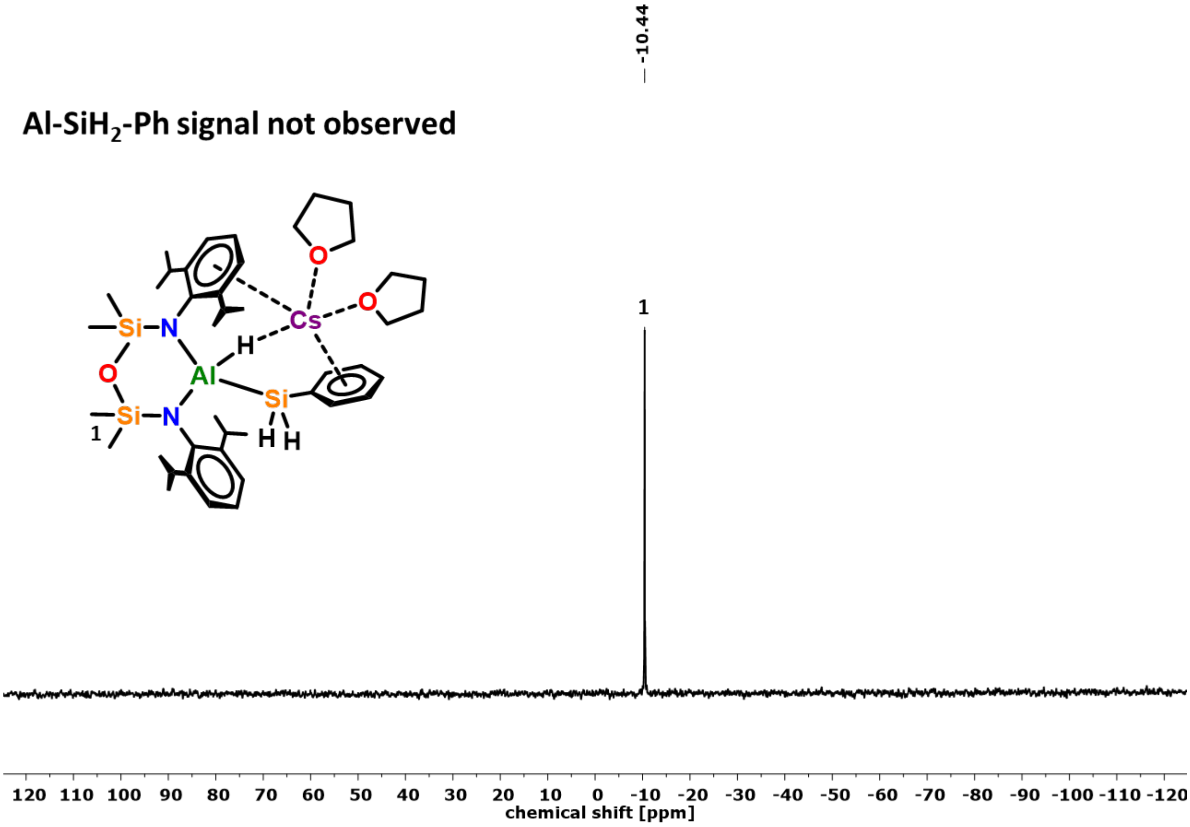

Figure S51. ^{29}Si-INEPT NMR spectrum of **1-Cs·(THF)$_2$** in a mixture of benzene-d_6:THF-d_8 (4:1).

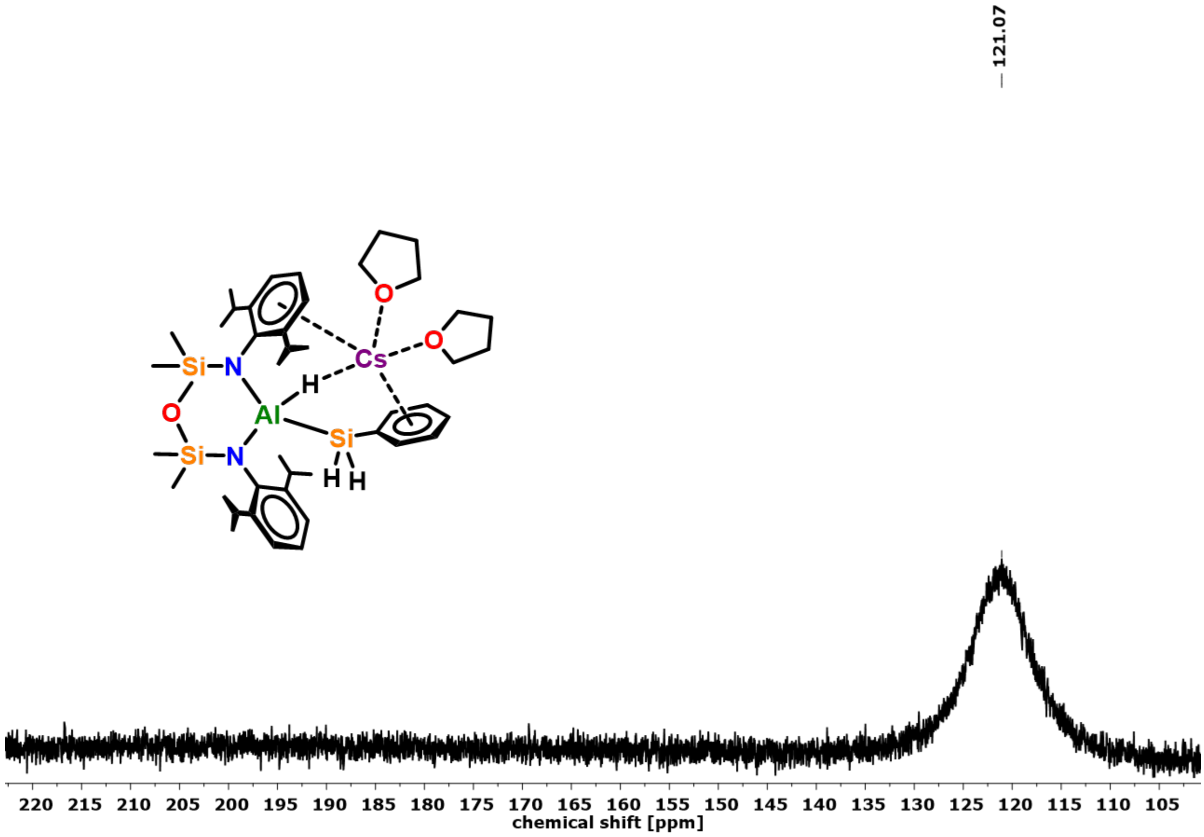

Figure S52. ^{27}Al NMR spectrum of **1-Cs·(THF)$_2$** in a mixture of benzene-d_6:THF-d_8 (4:1).

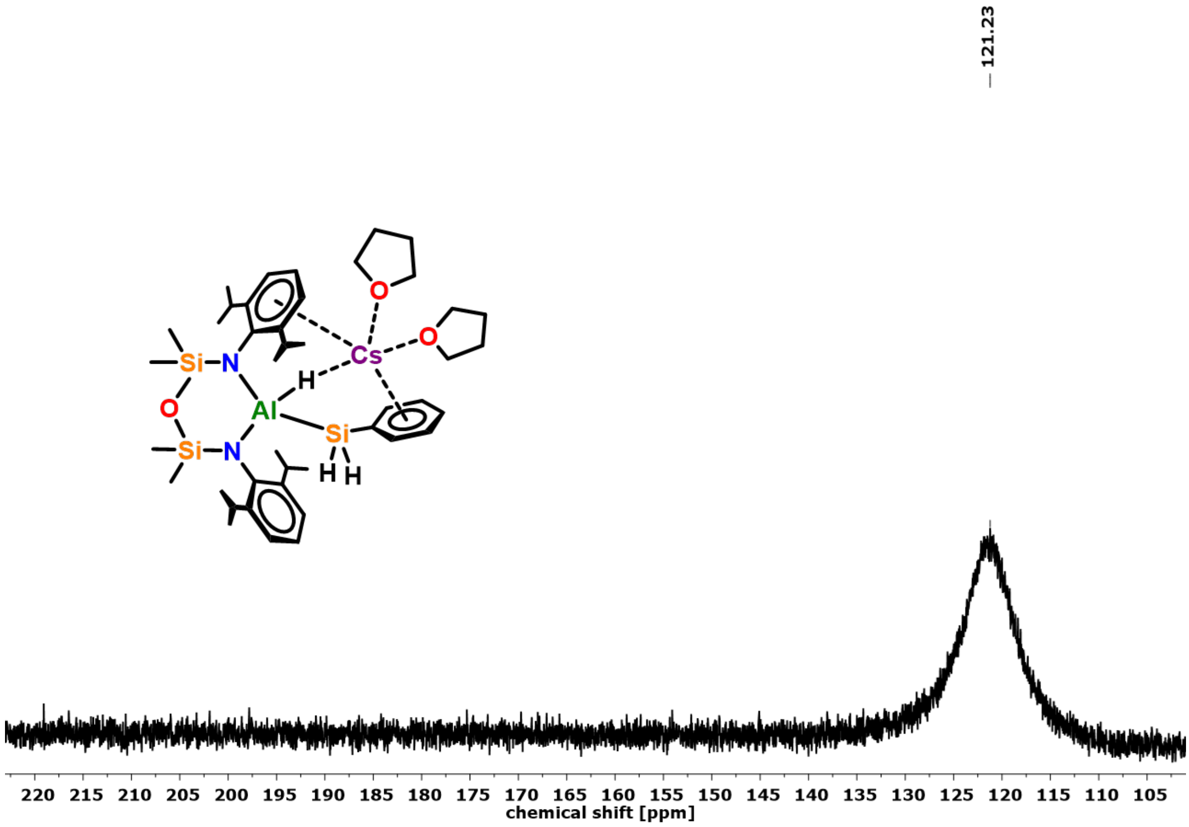

Figure S53. ^{27}Al{1H} NMR spectrum of **1-Cs·(THF)$_2$** in a mixture of benzene-d_6:THF-d_8 (4:1).

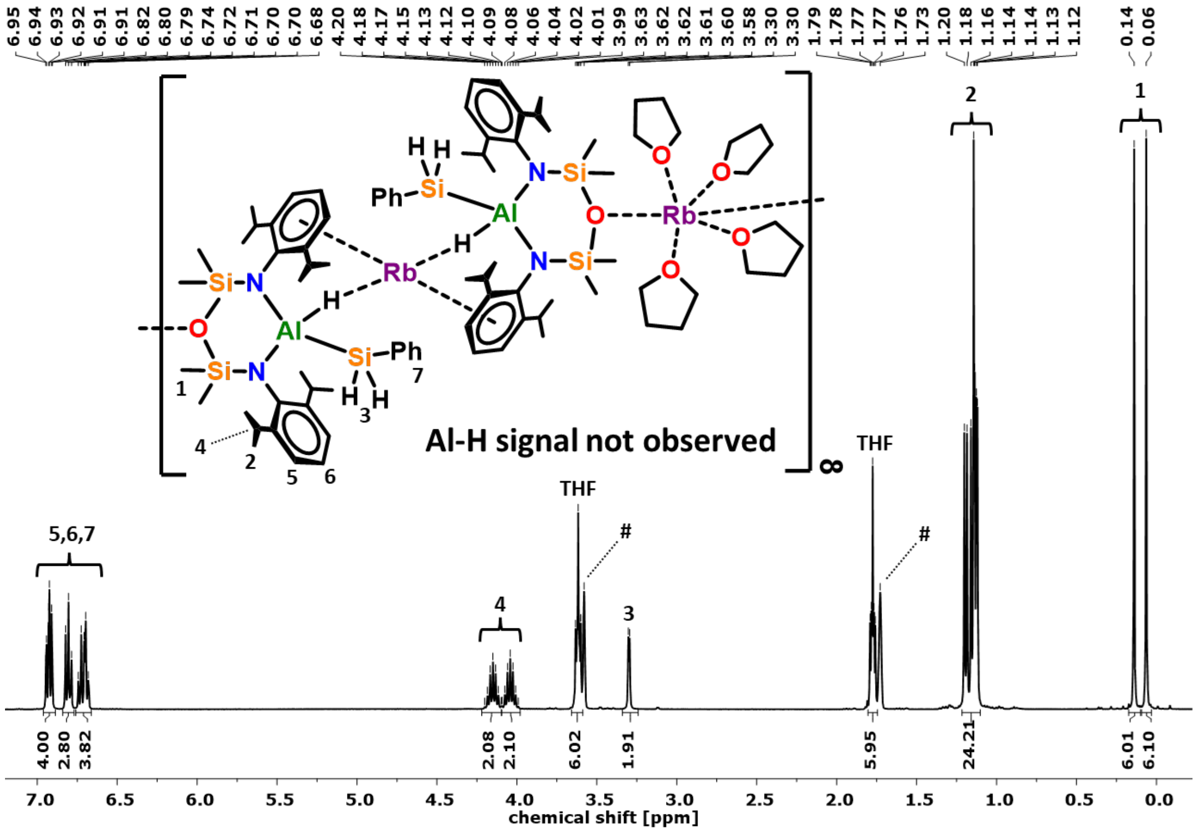

Figure S54. ^{1}H NMR spectrum of **2** in THF-d_8. Residual signal for deuterated solvent marked with #.

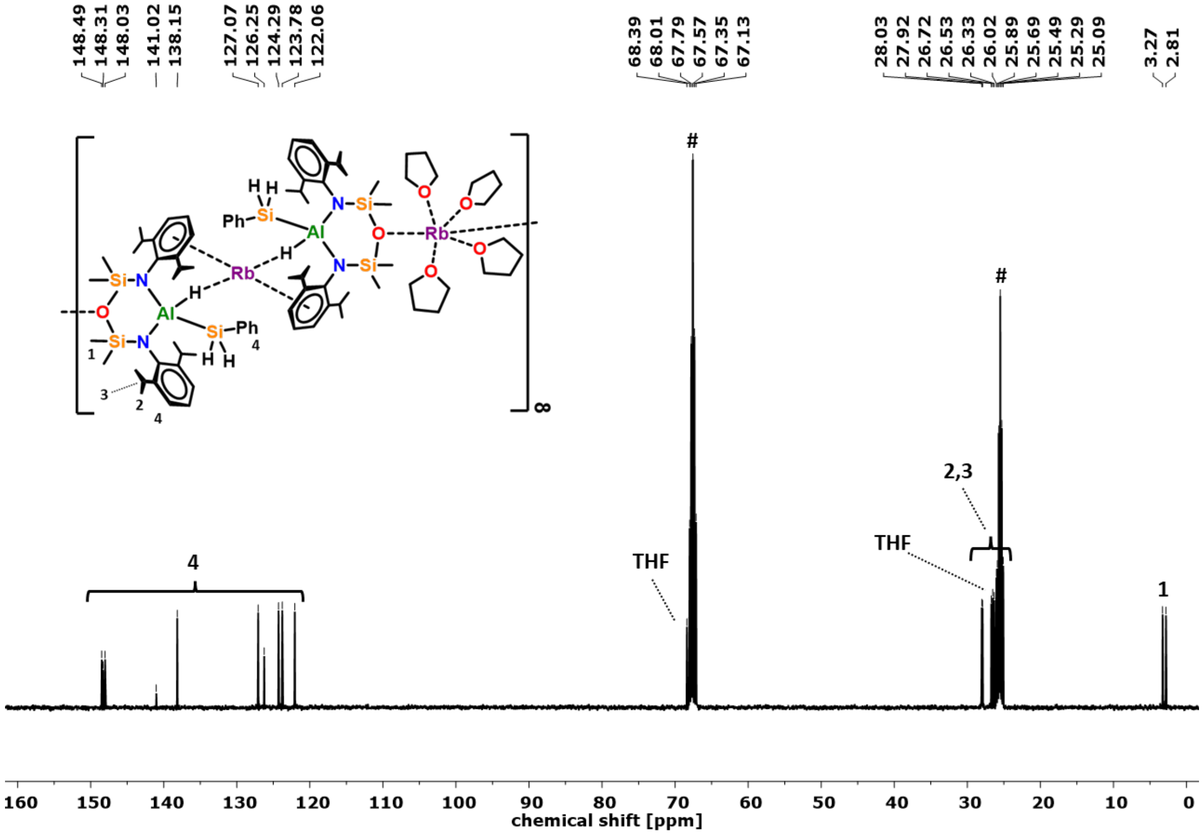

Figure S55. ^{13}C NMR spectrum of **2** in THF-d_8. Residual signal for deuterated solvent marked with #.

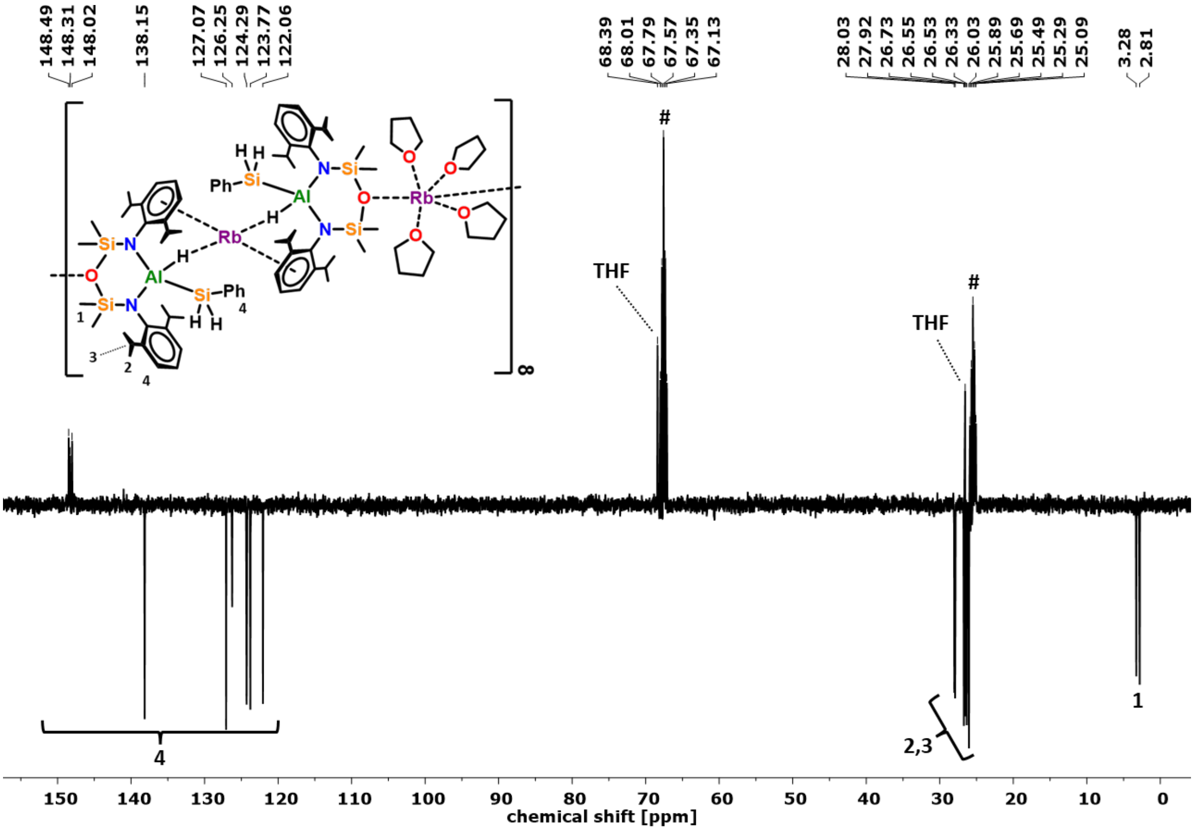

Figure S56. ^{13}C-DEPTQ NMR spectrum of **2** in THF-d_8. Residual signal for deuterated solvent marked with #.

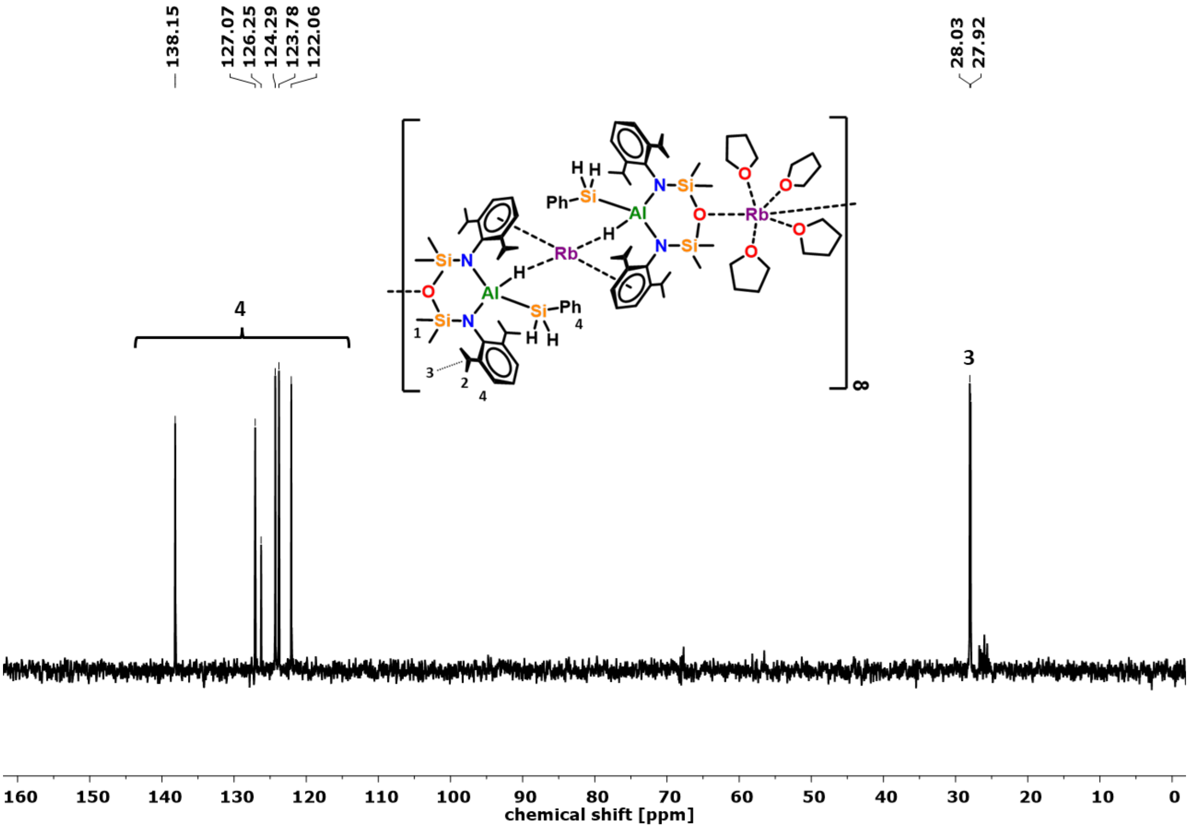

Figure S57. ^{13}C-DEPT-90 NMR spectrum of **2** in THF-d_8.

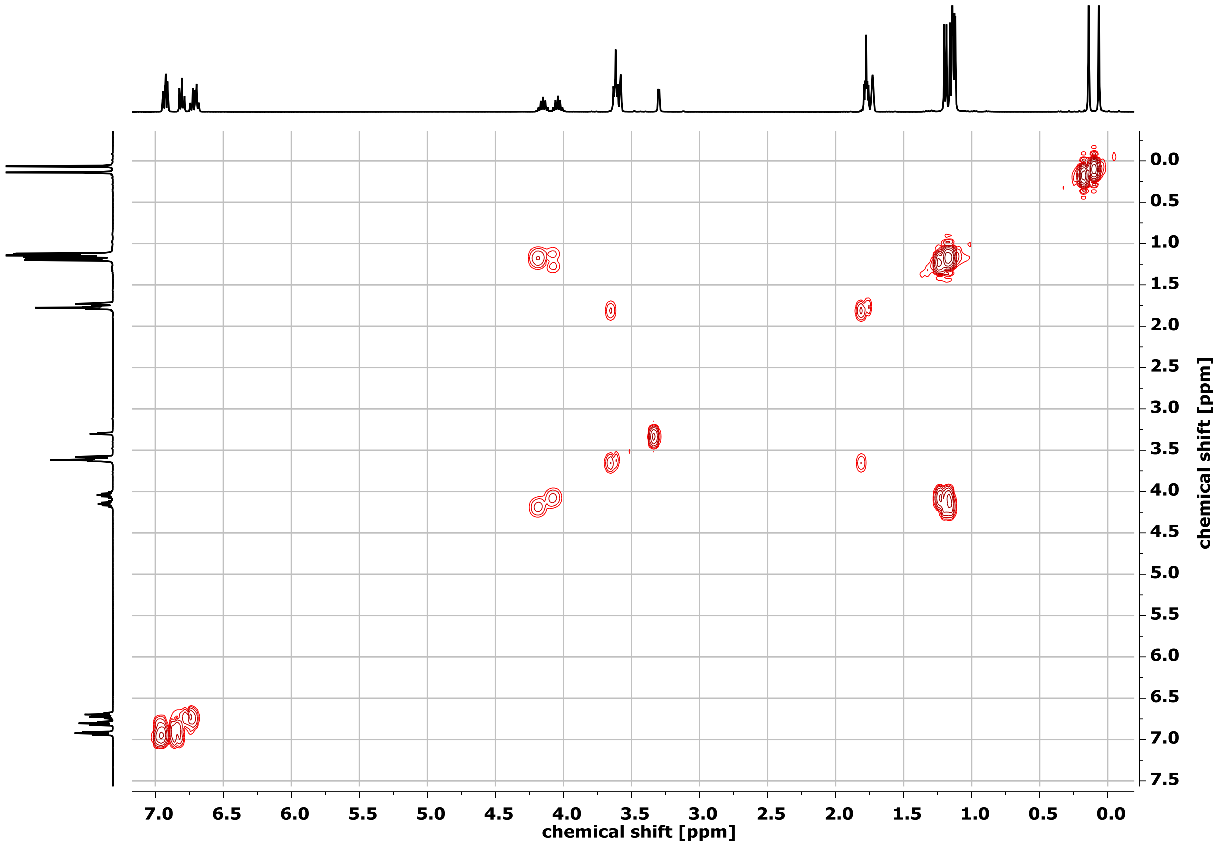

Figure S58. $^{1}H^{1}H$-COSY NMR spectrum of **2** in THF-d_8.

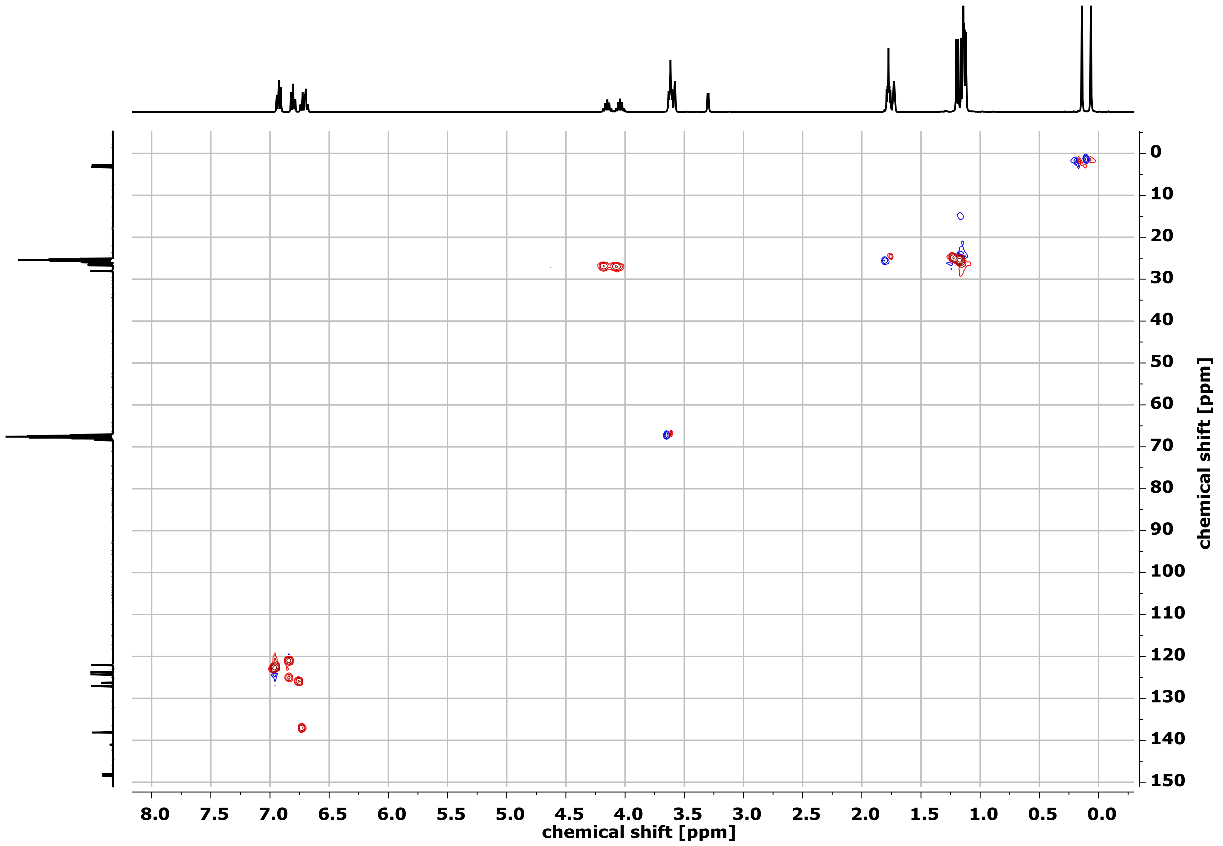

Figure S59. $^{1}H^{13}C$-HSQC NMR spectrum of **2** in THF-d_8.

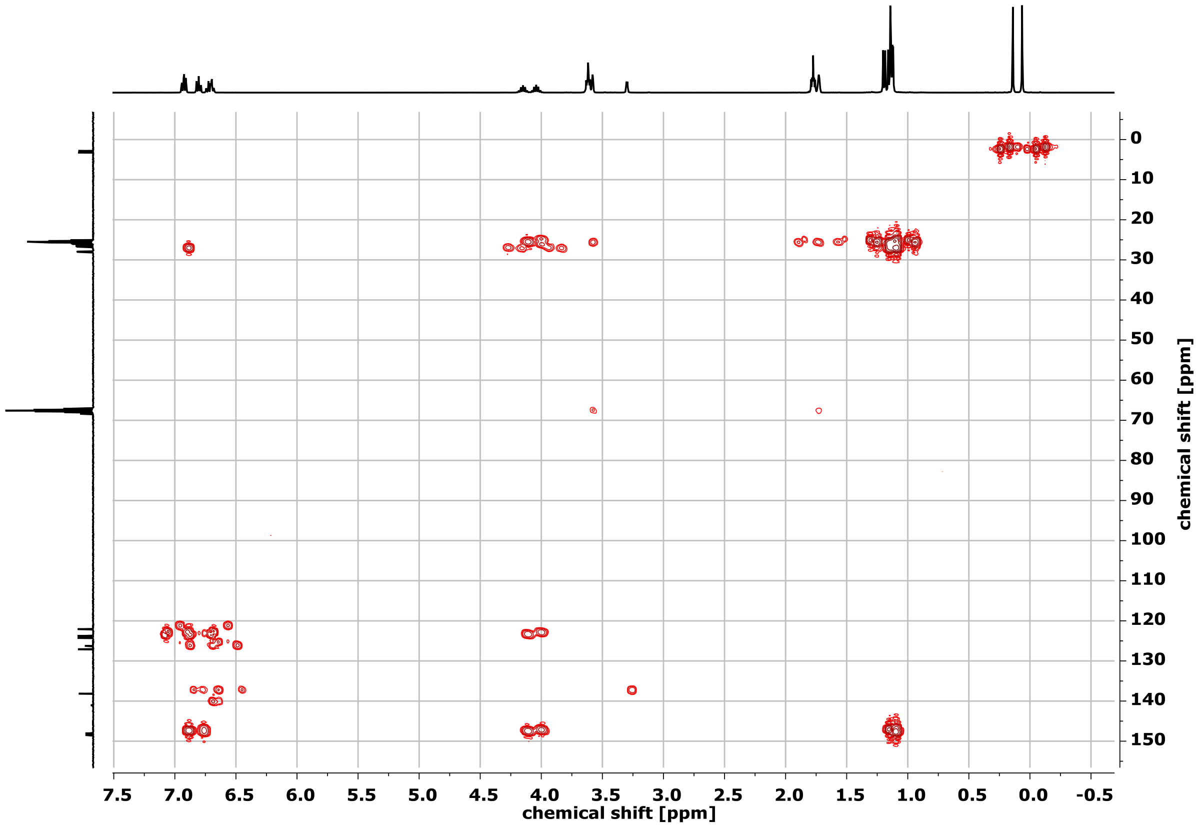

Figure S60. $^{1}H^{13}C$-HMBC NMR spectrum of **2** in THF-d_8.

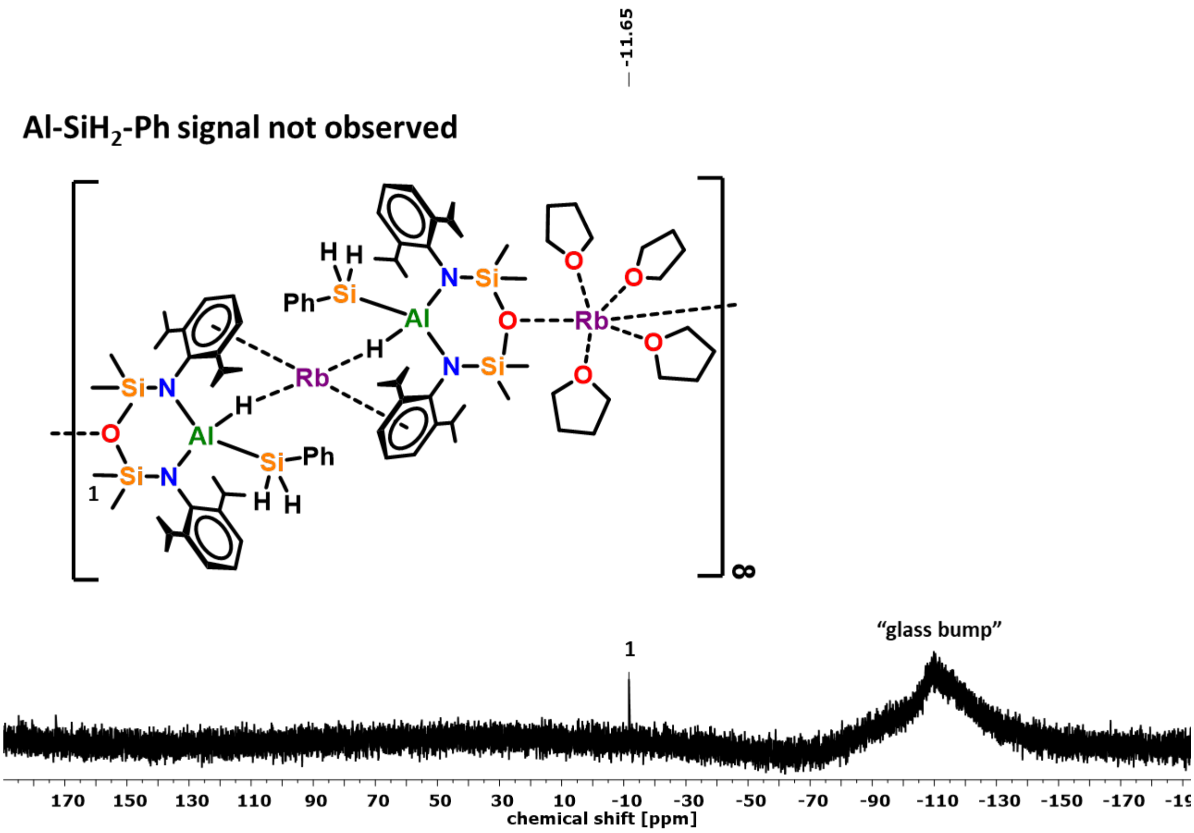

Figure S61. ^{29}Si NMR spectrum of **2** in THF-d_8.

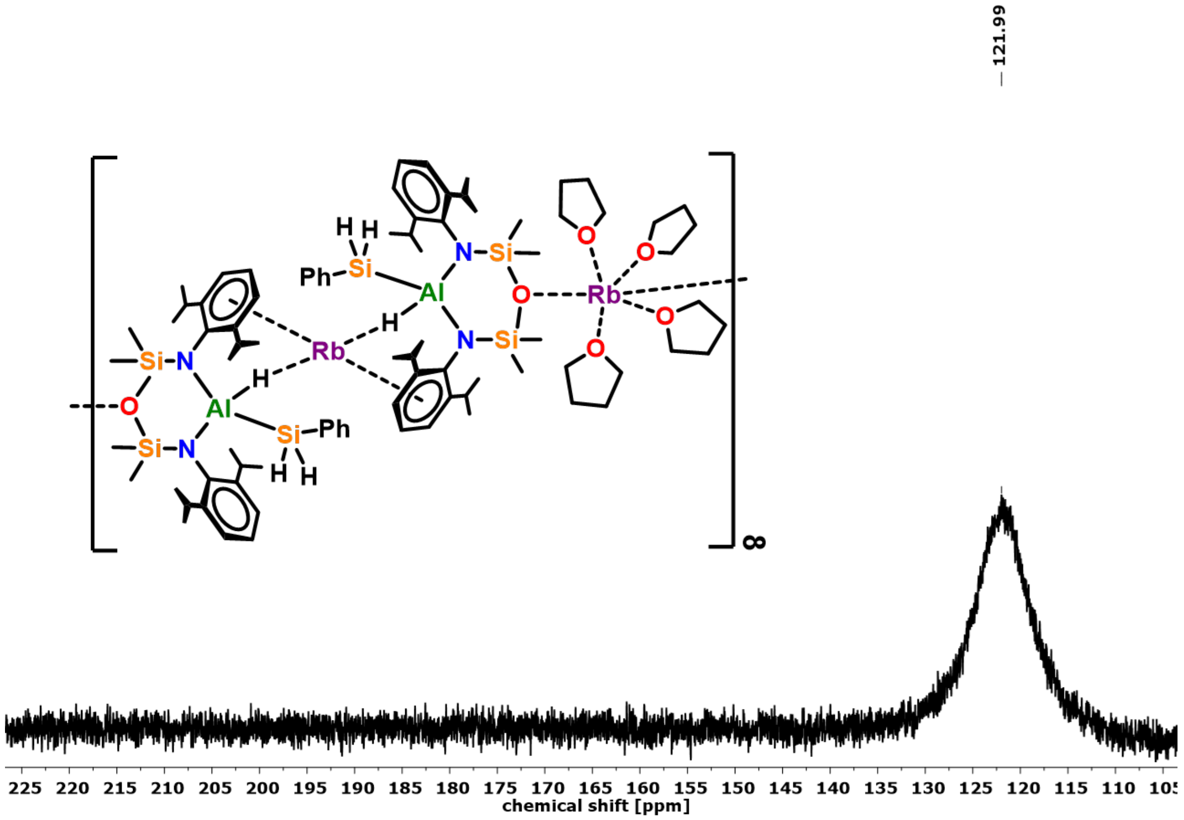

Figure S62. ^{27}Al NMR spectrum of **2** in THF-d_8.

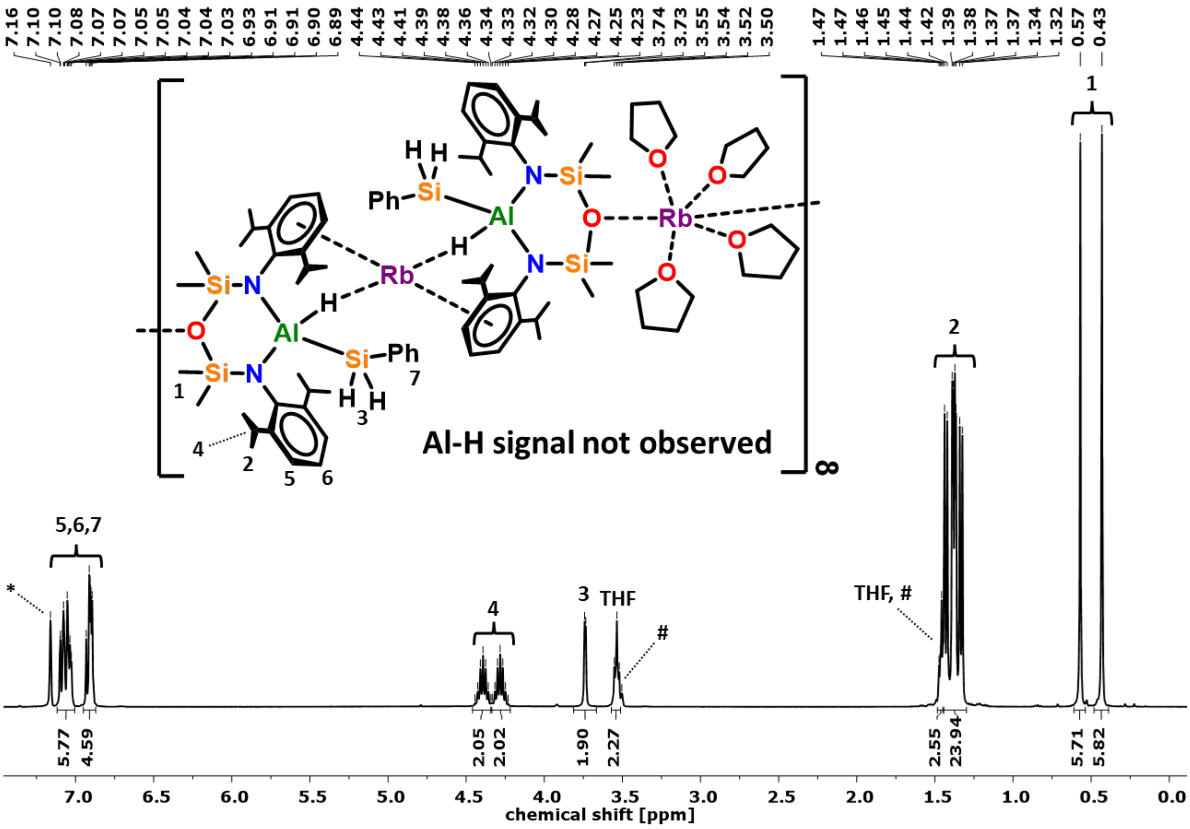

Figure S63. ^{1}H NMR spectrum of **2** in a mixture of benzene-d_6:THF-d_8 (10:1). Residual signal for deuterated solvents marked with * and #.

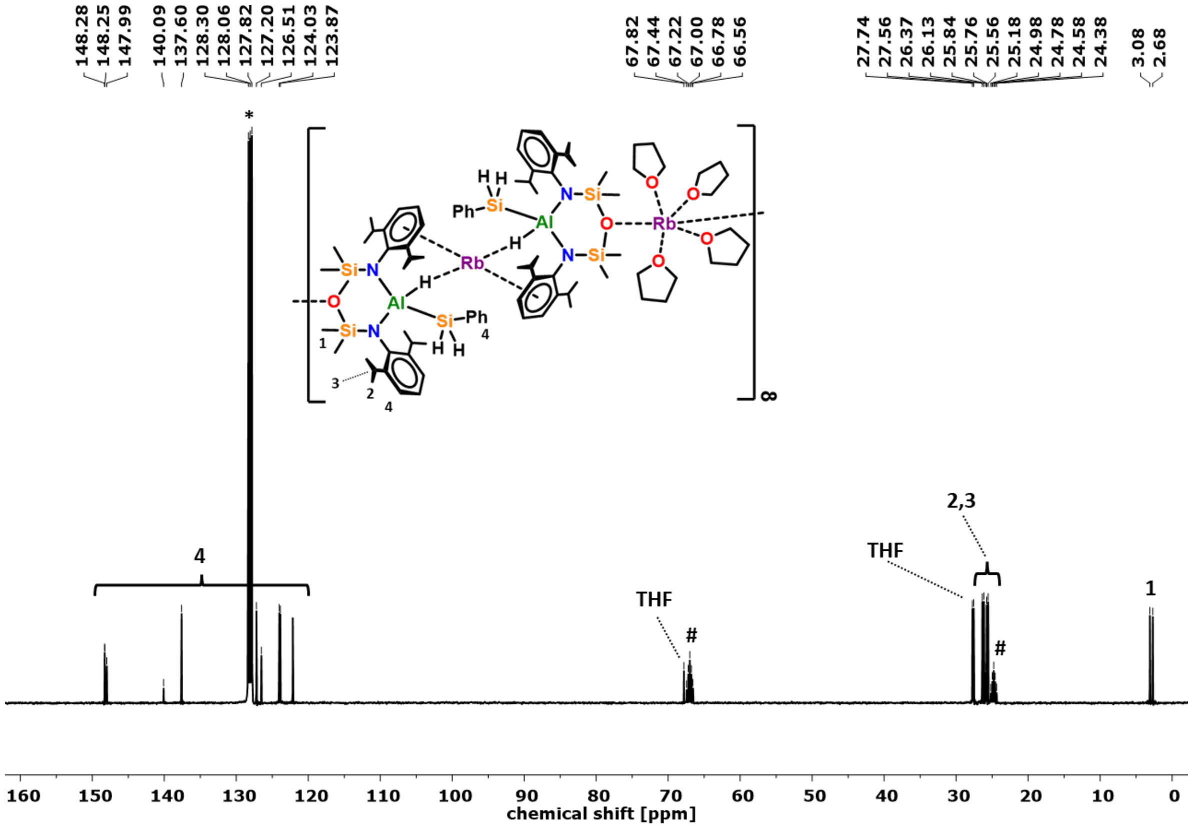

Figure S64. ^{13}C NMR spectrum of **2** in a mixture of benzene-d_6:THF-d_8 (10:1). Residual signal for deuterated solvents marked with * and #.

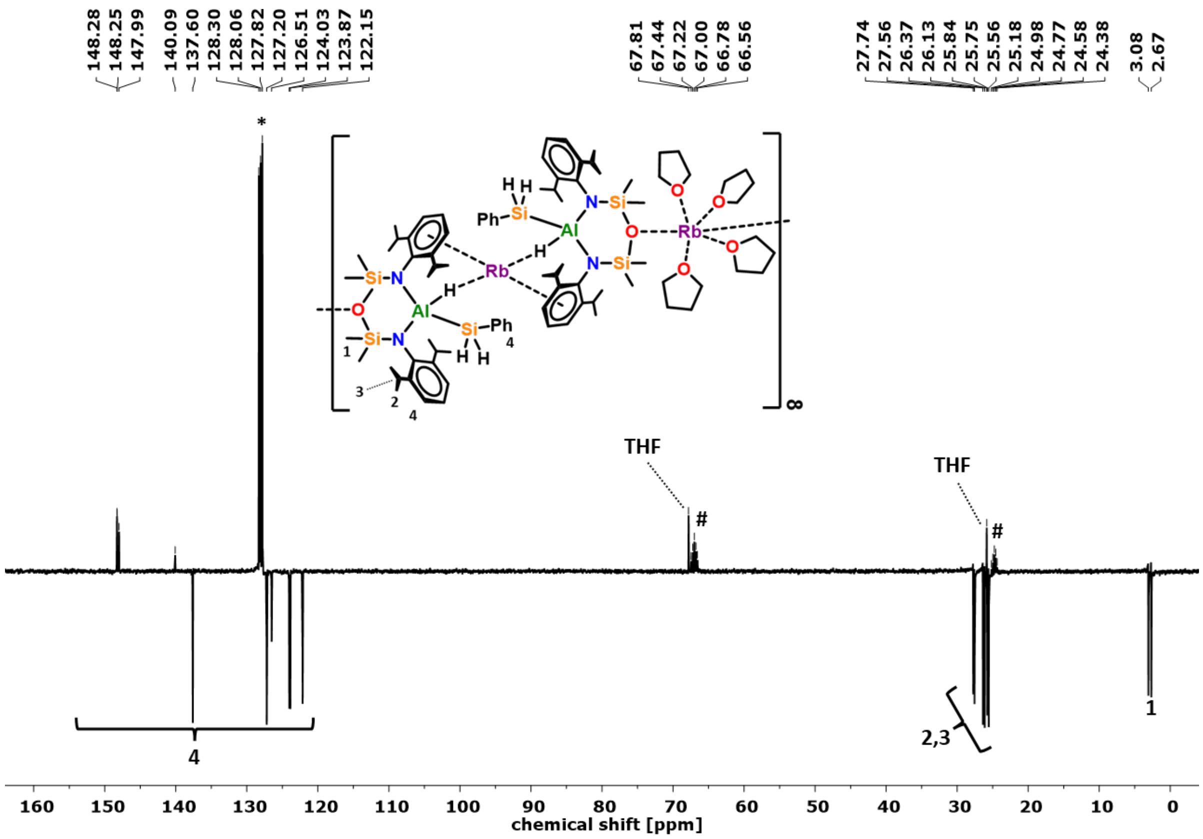

Figure S65. ^{13}C-DEPTQ NMR spectrum of **2** in a mixture of benzene-d_6:THF-d_8 (10:1). Residual signal for deuterated solvents marked with * and #.

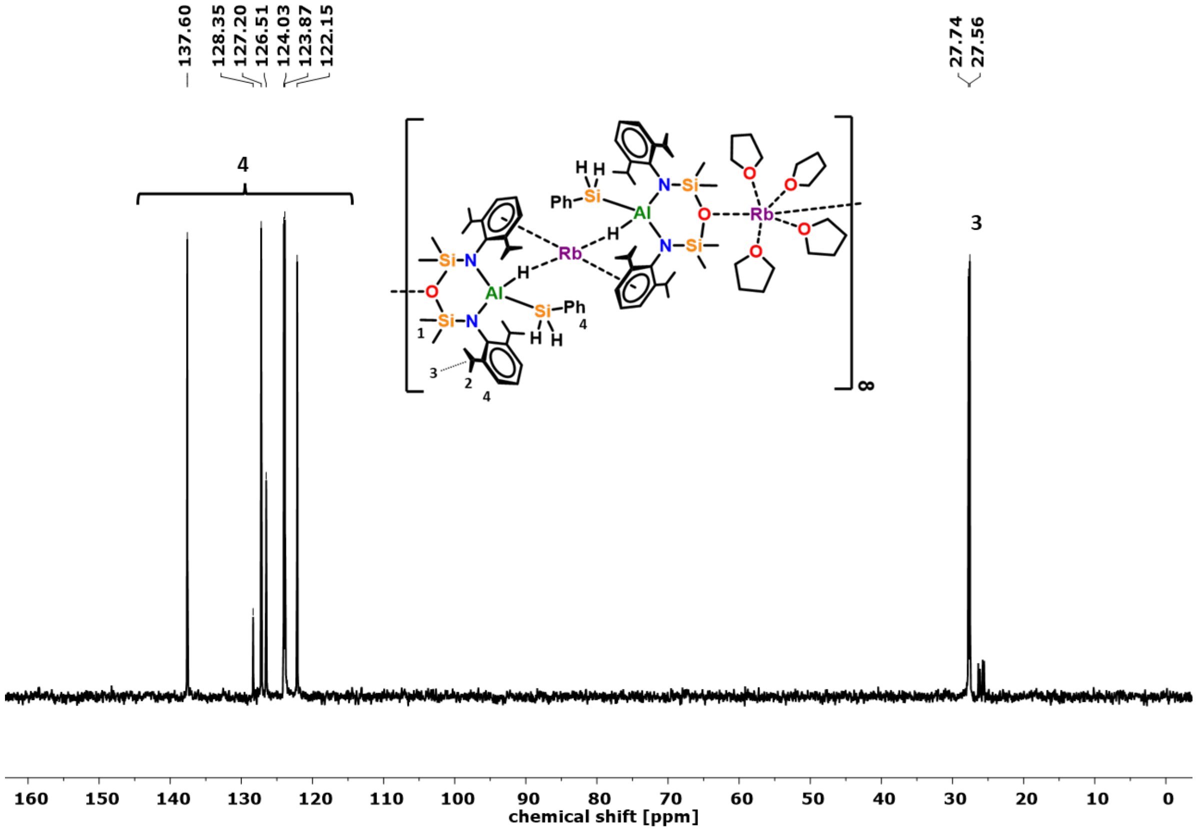

Figure S66. ^{13}C-DEPT-90 NMR spectrum of **2** in a mixture of benzene-d_6:THF-d_8 (10:1). Residual signal for deuterated solvents marked with * and #.

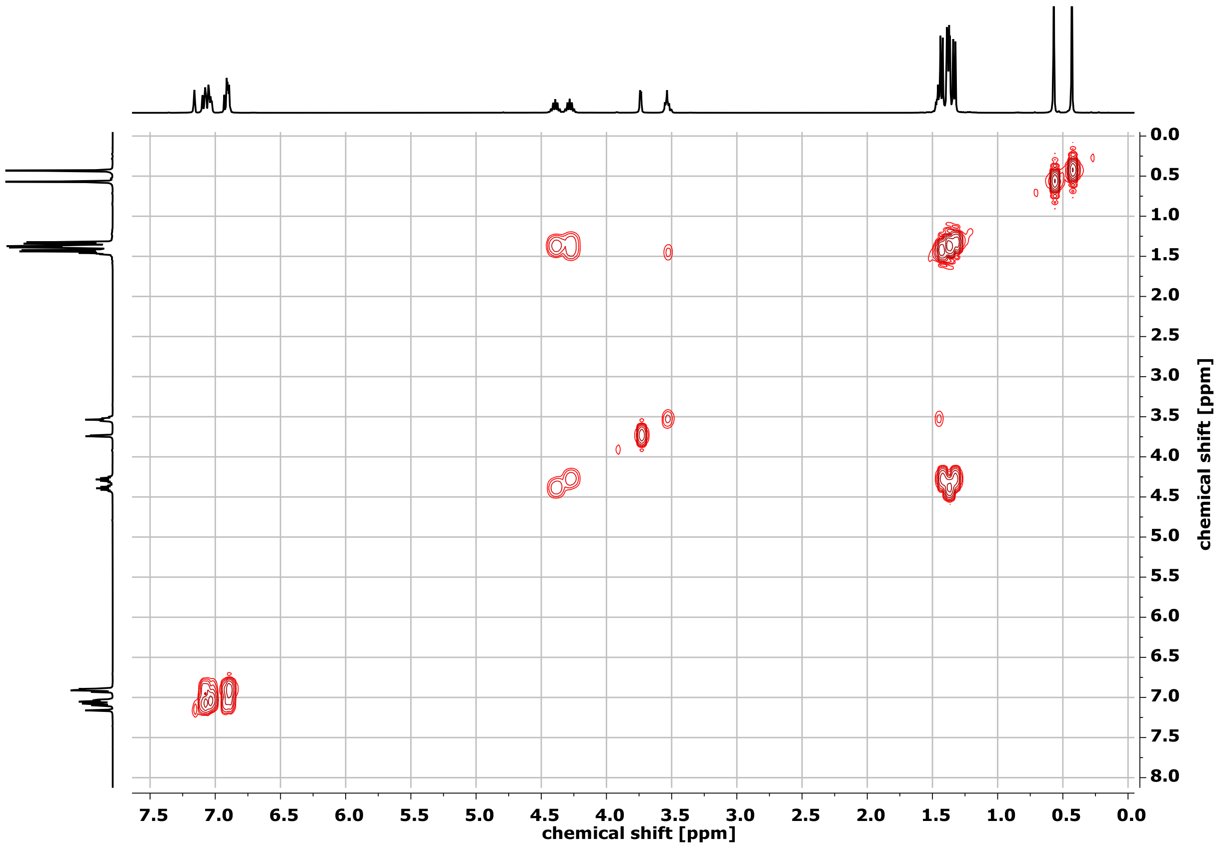

Figure S67. ^{1}H^{1}H-COSY NMR spectrum of **2** in a mixture of benzene-d_6:THF-d_8 (10:1). Residual signal for deuterated solvents marked with * and #.

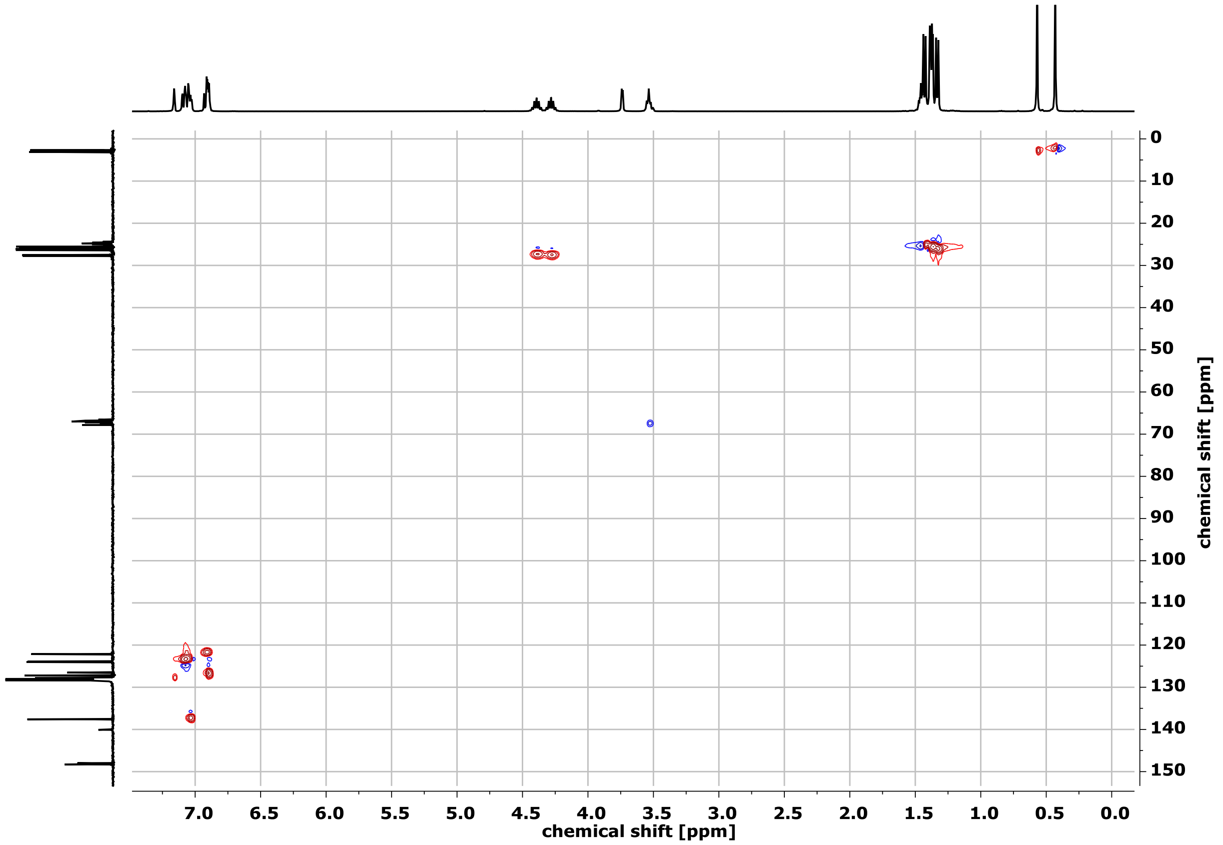

Figure S68. $^1H^{13}C$-HSQC NMR spectrum of **2** in a mixture of benzene-d_6:THF-d_8 (10:1). Residual signal for deuterated solvents marked with * and #.

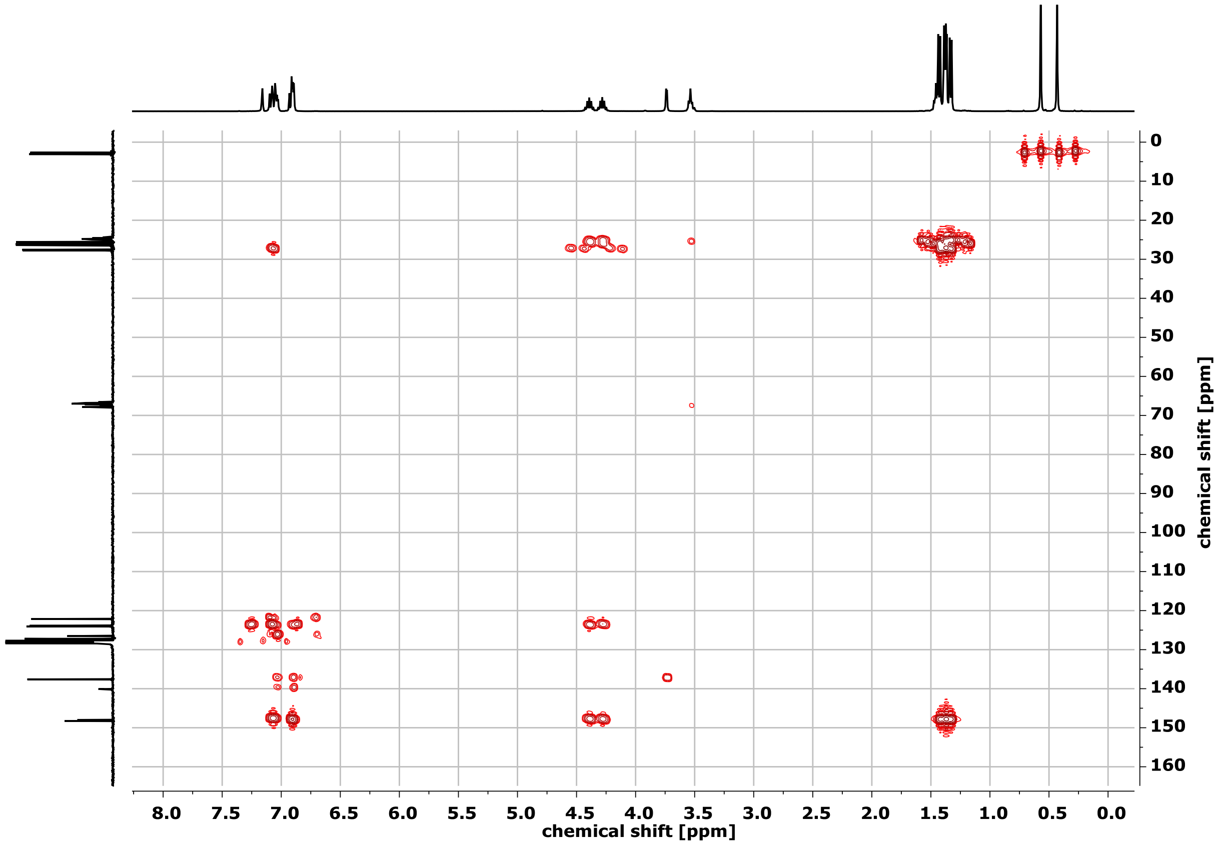

Figure S69. $^1H^{13}C$-HMBC NMR spectrum of **2** in a mixture of benzene-d_6:THF-d_8 (10:1). Residual signal for deuterated solvents marked with * and #.

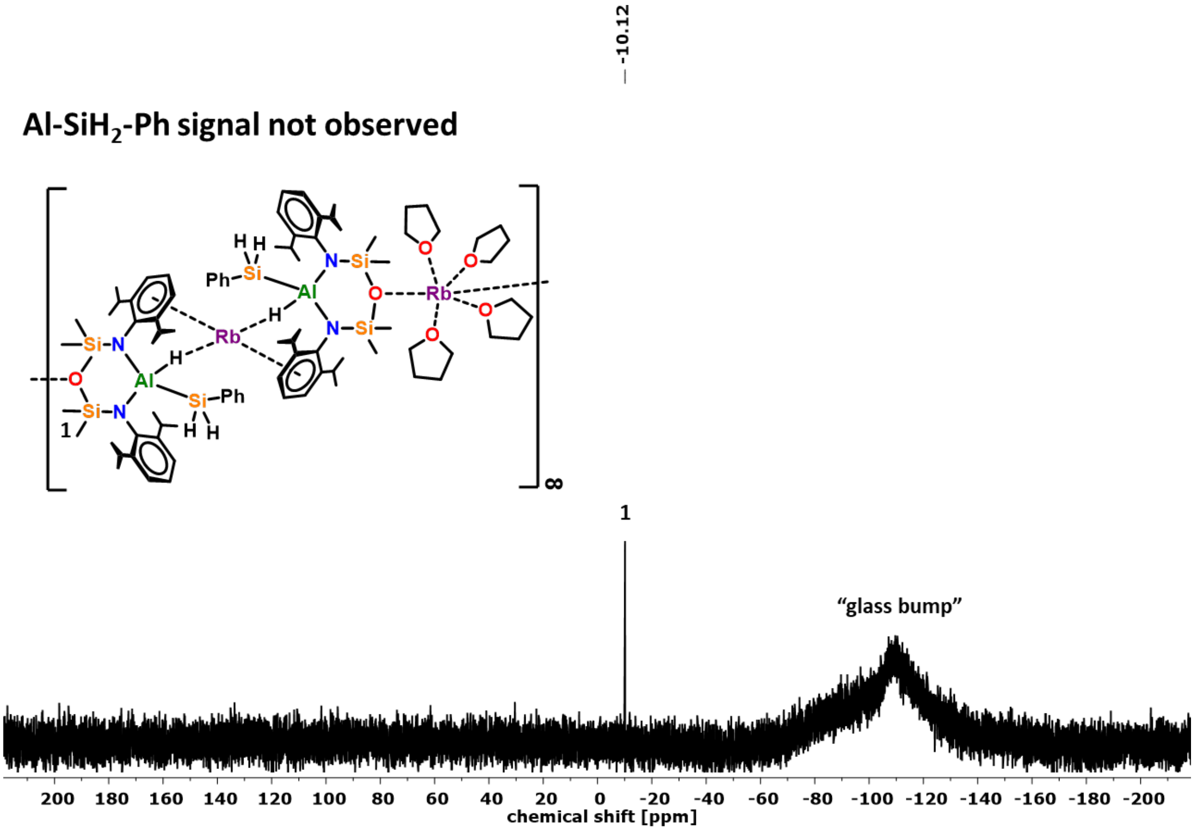

Figure S70. ^{29}Si NMR spectrum of **2** in a mixture of benzene-d_6:THF-d_8 (10:1). Residual signal for deuterated solvents marked with * and #.

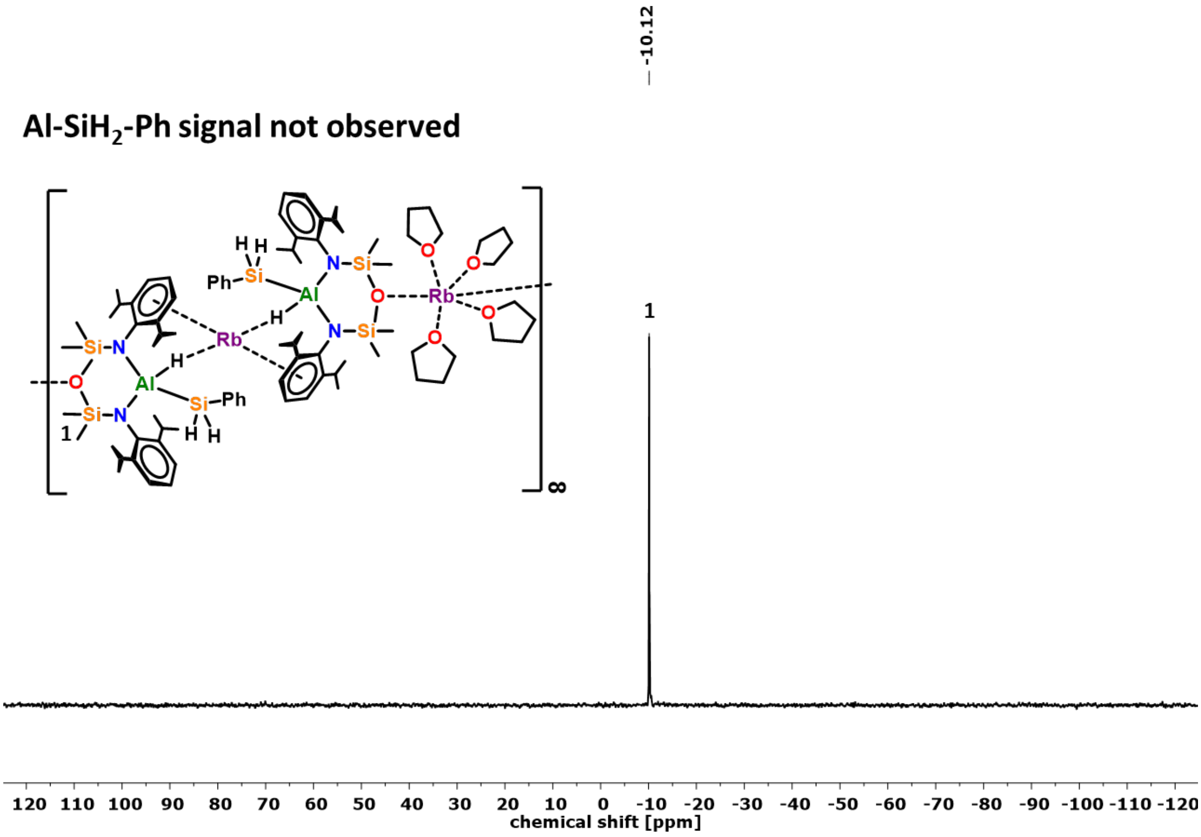

Figure S71. ^{29}Si-INEPT NMR spectrum of **2** in a mixture of benzene-d_6:THF-d_8 (10:1). Residual signal for deuterated solvents marked with * and #.

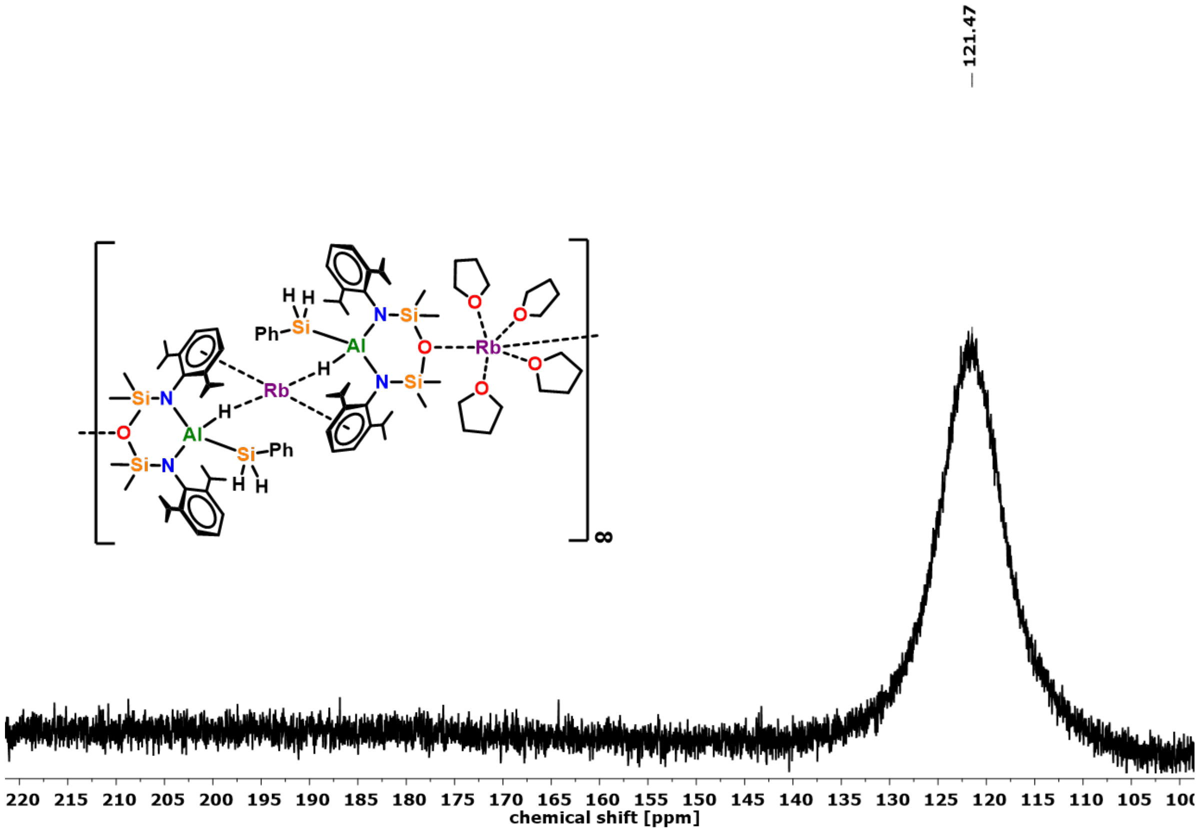

Figure S72. ^{27}Al NMR spectrum of **2** in a mixture of benzene-*d*$_6$:THF-*d*$_8$ (10:1). Residual signal for deuterated solvents marked with * and #.

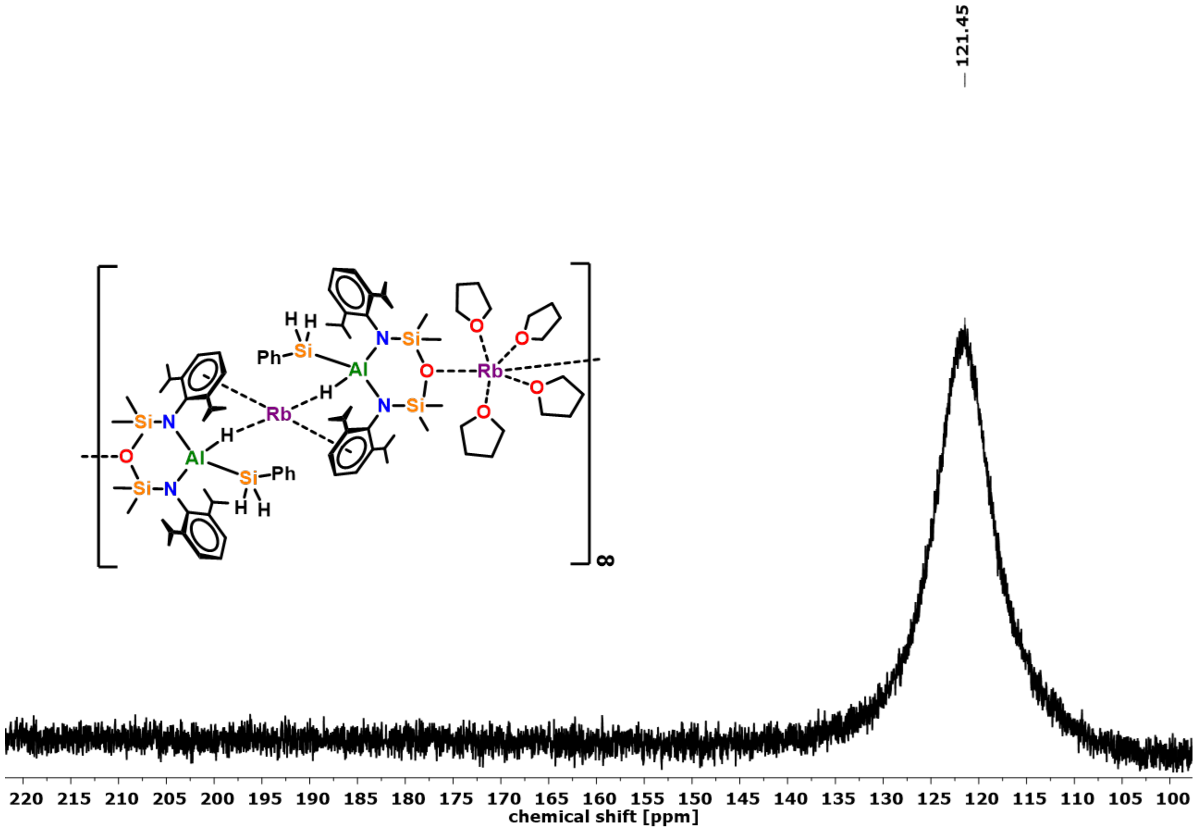

Figure S73. ^{27}Al{1H} NMR spectrum of **2** in a mixture of benzene-*d*$_6$:THF-*d*$_8$ (10:1). Residual signal for deuterated solvents marked with * and #.

4) Selected infrared spectra

Infrared vibrational spectra, shown in Figures **S74** and **S75**, were obtained by Fourier transform infrared (FT-IR) recording on solid samples, using a Bruker Tensor 27 FT-IR spectrometer.

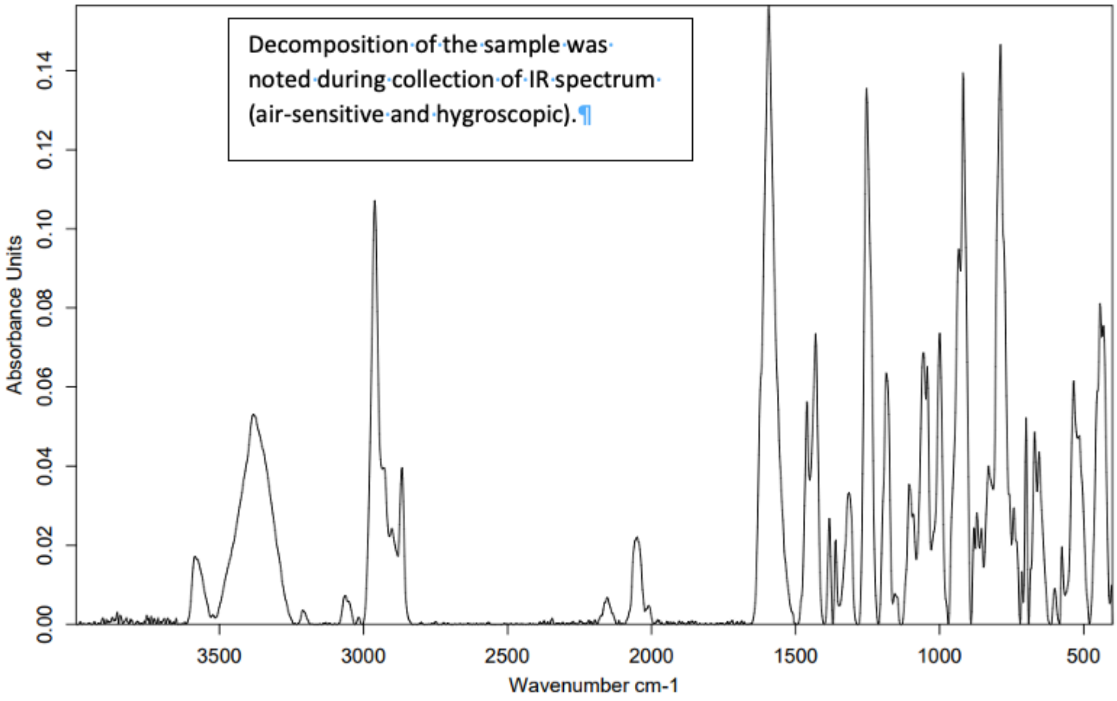

Figure S74. IR vibrational spectrum of **1-Li·(Et$_2$O)**

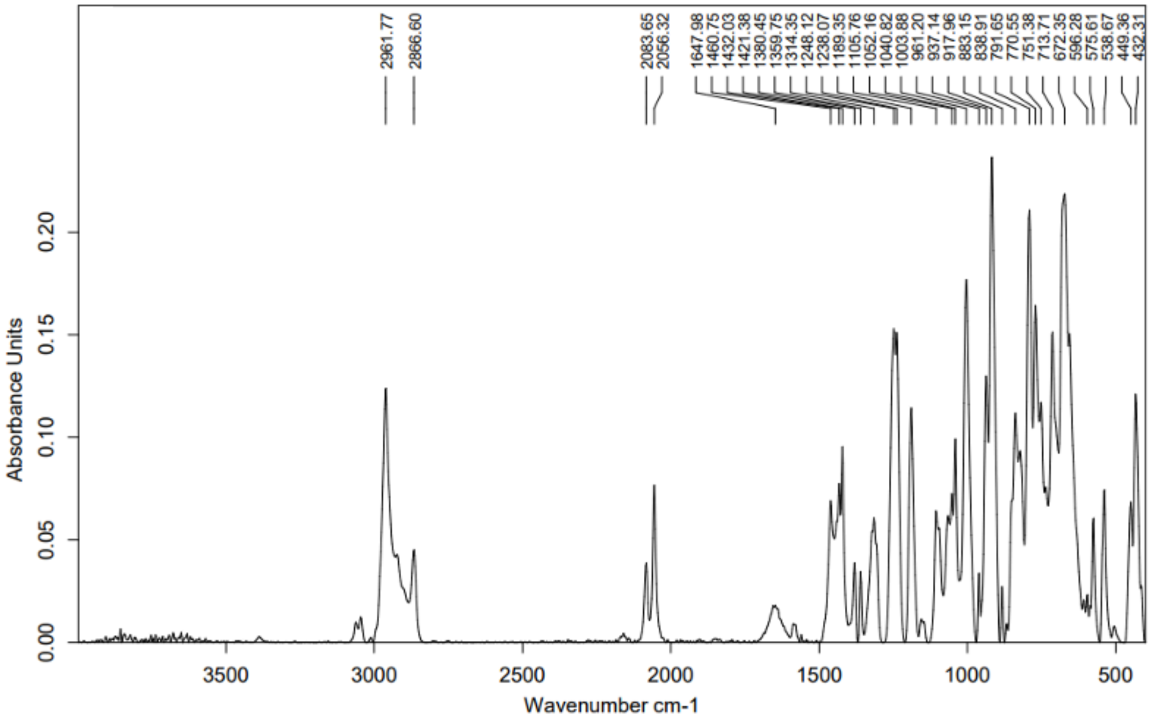

Figure S75. IR vibrational spectrum of **1-Na·(Et$_2$O)**

Infrared vibrational spectra, shown in Figures **S76** – **S78**, were obtained as Nujol mulls on NaCl plates. Mulls were prepared in the glove box using anhydrous Nujol, which was dried and stored over activated 4 Å molecular sieves under argon, and then transferred to the spectrometer in a desiccator. Spectra were recorded on a Nicolet 360 FTIR spectrometer spanning 4000-400 cm^{-1} at room temperature.

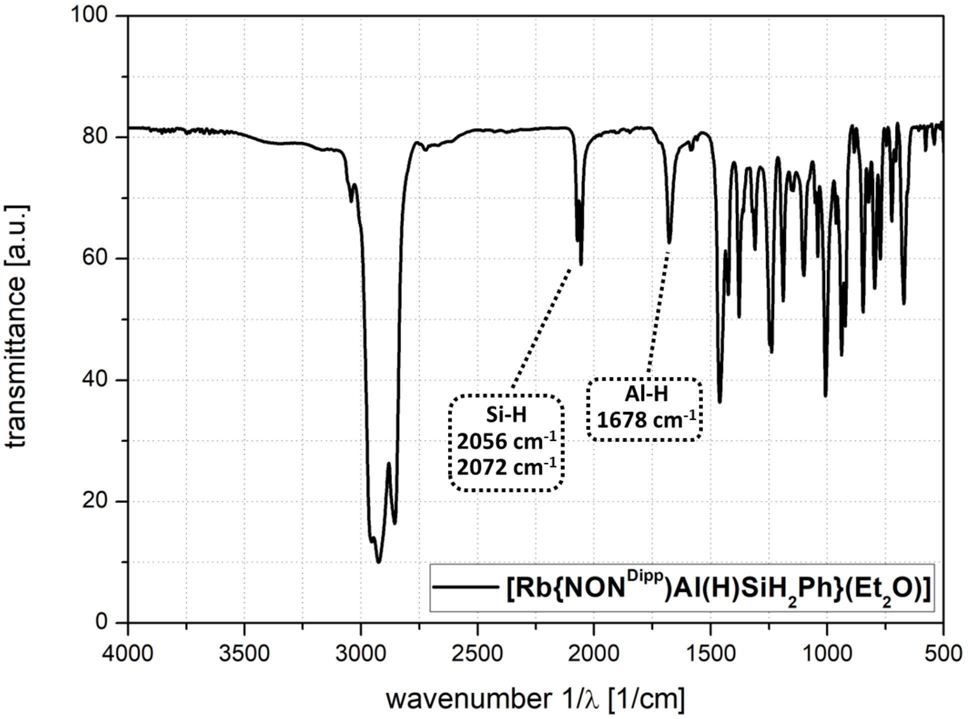

Figure S76. IR vibrational spectrum of **1-Rb·(Et$_2$O)**

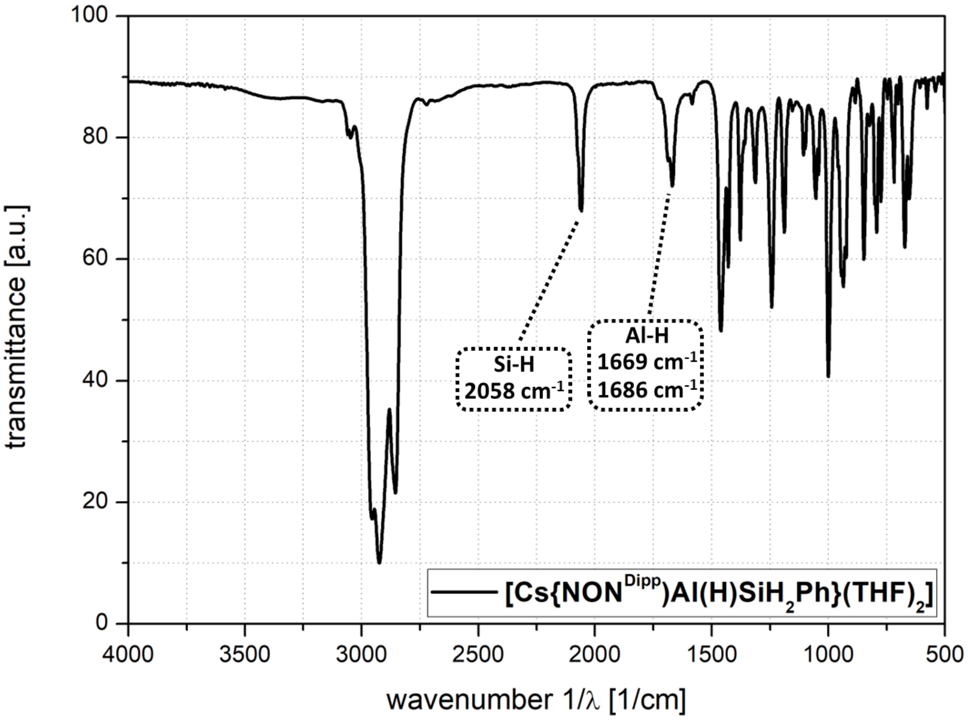

Figure S77. IR vibrational spectrum of **1-Cs·(THF)$_2$**.

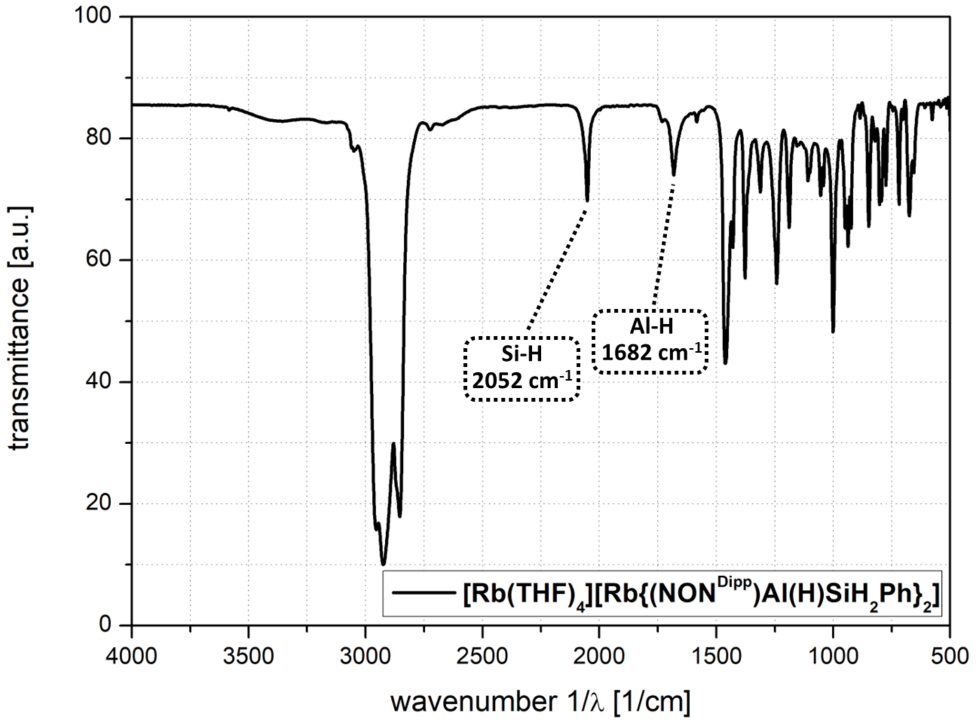

Figure S78. IR vibrational spectrum of **2**.

6) References

[S1] R. J. Schwamm, M. D. Anker, M. Lein, M. P. Coles. Reduction vs. addition: The reaction of an aluminyl anion with 1,3,5,7-cyclooctatetraene. *Angew. Chem. Int. Ed*. **2019**, *58*, 1489–1493.

[S2] M. J. Evans, M. D. Anker, C. L. McMullin, S. E. Neale, M. P. Coles. Dihydrogen Activation by Lithium- and Sodium-Aluminyls. *Angew. Chem. Int. Ed.* **2021**, *60*, 22289−22292.

[S3] K. Grubel, W. W. Brennessel, B. Q. Mercado, P. L. Holland. Alkali Metal Control over N–N Cleavage in Iron Complexes. *J. Am. Chem. Soc.* **2014**, *136*, 16807−16816.

[S4] T. X. Gentner, M. J. Evans, A. R. Kennedy, S. E. Neale, C. L. McMullin, M. P. Coles, R. E. Mulvey. Rubidium and caesium aluminyls: synthesis, structures and reactivity in C–H bond activation of benzene. *Chem. Commun.* **2022**, *58*, 1390−1393.

[S5] M. J. Evans, M. D. Anker, M. P. Coles. Oxidative Addition of Hydridic, Protic, and Nonpolar E–H Bonds (E = Si, P, N, or O) to an Aluminyl Anion. *Inorg. Chem.* **2021**, *60*, 4772-4778.

[S6] J. Cosier, A. M. Glazer. A Nitrogen-Gas-Stream Cryostat for General X-ray Diffraction Studies. *J. Appl. Cryst.* **1986**, *19*, 105–107.

[S7] R. H. Blessing. An Empirical Correction for Absorption Anisotropy. *Acta Cryst. Sect. A* **1995**, *51*, 33–38.

[S8] G. M. Sheldrick. A Short History of *SHELX*. *Acta Cryst.* **2008**, *A64*, 112–122.

[S9] G. M. Sheldrick, "SHELX-97 and SHELXL-97, Program for Crystal Structure Solution and Refinement", University of Göttingen, Göttingen, **1997**.

[S10] "CrysAlisPro Software system, version 1.171.39.46", Rigaku Corporation, Oxford UK, **2018**.

[S11] O. V. Dolomanov, L. J. Bourhis, R. J. Gildea, J. A. K. Howard, H. Puschmann. *OLEX2*: a complete structure solution, refinement and analysis program. *J. Appl. Cryst.* **2009**, *42*, 339–341.

[S12] G. M. Sheldrick. Crystal structure refinement with *SHELXL*. *Acta Cryst. Sect. C* **2015**, *71*, 3–8.

[S13] L. J. Farrugia. *WinGX* and *ORTEP for Windows*: an update. *J. Appl. Cryst.* **2012**, *45*, 849–854.
